# Karyon: a computational framework for the diagnosis of hybrids, aneuploids, and other nonstandard architectures in genome assemblies

**DOI:** 10.1093/gigascience/giac088

**Published:** 2022-10-07

**Authors:** Miguel A Naranjo-Ortiz, Manu Molina, Diego Fuentes, Verónica Mixão, Toni Gabaldón

**Affiliations:** Centre for Genomic Regulation (CRG), The Barcelona Institute of Science and Technology, Barcelona 08003, Spain; Health and Life Sciences, Universitat Pompeu Fabra (UPF), Barcelona 08003, Spain; Biology Department, Clark University, Worcester, MA 01610, USA; Naturhistoriskmuseum, University of Oslo, Oslo 0562, Norway; Centre for Genomic Regulation (CRG), The Barcelona Institute of Science and Technology, Barcelona 08003, Spain; Health and Life Sciences, Universitat Pompeu Fabra (UPF), Barcelona 08003, Spain; Life Sciences Department, Barcelona Supercomputing Centre (BSC-CNS), Barcelona 08034, Spain; Life Sciences Department, Barcelona Supercomputing Centre (BSC-CNS), Barcelona 08034, Spain; Institute for Research in Biomedicine (IRB Barcelona), The Barcelona Institute of Science and Technology, Barcelona 08028, Spain; Centre for Genomic Regulation (CRG), The Barcelona Institute of Science and Technology, Barcelona 08003, Spain; Health and Life Sciences, Universitat Pompeu Fabra (UPF), Barcelona 08003, Spain; Life Sciences Department, Barcelona Supercomputing Centre (BSC-CNS), Barcelona 08034, Spain; Institute for Research in Biomedicine (IRB Barcelona), The Barcelona Institute of Science and Technology, Barcelona 08028, Spain; Centre for Genomic Regulation (CRG), The Barcelona Institute of Science and Technology, Barcelona 08003, Spain; Health and Life Sciences, Universitat Pompeu Fabra (UPF), Barcelona 08003, Spain; Life Sciences Department, Barcelona Supercomputing Centre (BSC-CNS), Barcelona 08034, Spain; Institute for Research in Biomedicine (IRB Barcelona), The Barcelona Institute of Science and Technology, Barcelona 08028, Spain; ICREA, Pg. Lluís Companys 23, Barcelona 08010, Spain; Centro de Investigación Biomédica en Red de Enfermedades Infecciosas, Barcelona 28029, Spain

**Keywords:** genome assembly, heterozygosity, hybridization, polyploidy, aneuploidy

## Abstract

**Background:**

Recent technological developments have made genome sequencing and assembly highly accessible and widely used. However, the presence in sequenced organisms of certain genomic features such as high heterozygosity, polyploidy, aneuploidy, heterokaryosis, or extreme compositional biases can challenge current standard assembly procedures and result in highly fragmented assemblies. Hence, we hypothesized that genome databases must contain a nonnegligible fraction of low-quality assemblies that result from such type of intrinsic genomic factors.

**Findings:**

Here we present Karyon, a Python-based toolkit that uses raw sequencing data and *de novo* genome assembly to assess several parameters and generate informative plots to assist in the identification of nonchanonical genomic traits. Karyon includes automated *de novo* genome assembly and variant calling pipelines. We tested Karyon by diagnosing 35 highly fragmented publicly available assemblies from 19 different Mucorales (Fungi) species.

**Conclusions:**

Our results show that 10 (28.57%) of the assemblies presented signs of unusual genomic configurations, suggesting that these are common, at least for some lineages within the Fungi.

## Findings

• We present Karyon, a Python-based bioinformatic pipeline that integrates genome assembly and a series of structural analyses for the diagnosis of problematic genomic structures. Karyon is freely available in GitHub and as a docker container (https://github.com/Gabaldonlab/karyon).

• We applied Karyon to 35 highly fragmented, publicly available genome assemblies to identify putative undescribed deviations in genomic architecture that might have caused problems in a standard assembly process. From 35 assemblies, 10 presented features that suggested possible underlying biological factors as the likely cause of the observed assembly fragmentation. Even though our sample size is small and restricted to a single lineage (Mucoromycotina), our results suggest that the number of unreported deviations in genome architecture in Fungi is considerable. This is emphasized if we consider that most researchers that have produced low-quality assemblies are unlikely to publish their data.

## Introduction

Recent developments in high-throughput sequencing and bioinformatic tools have made the process of sequencing the genome of a new organism a routine task for many laboratories, especially those working on groups with small compact genomes (prokaryotes, fungi, many parasitic lineages). The success of a genome assembly is limited by technical aspects as well as by intrinsic properties of the sequenced genome. A successful assembly depends on the quality, design, and depth of the sequencing libraries, which must typically adapt to budget limitations. Naturally, if the sequencing methodology or the computational approaches are inappropriate, the resulting assembly will be poor (i.e., highly fragmented, incomplete, or misassembled). However, additional difficulties might arise independently of the methodology employed, due to intrinsic properties of the genome that interfere with genome assembly algorithms.

## Biological factors affecting genome assembly quality

**Figure 1:**
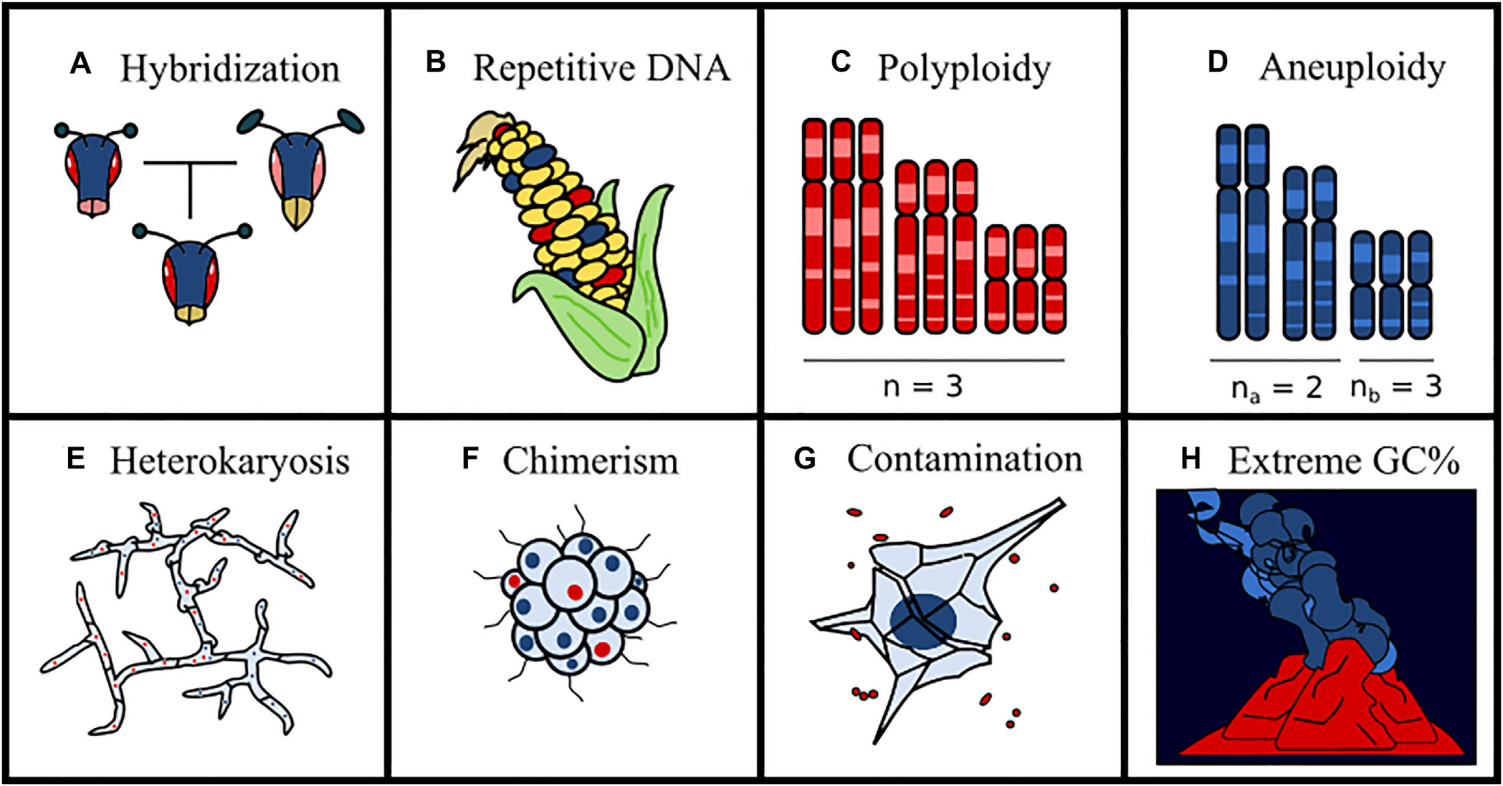
Biological factors that confound genome assembly process. Ploidy and aneuploidy increase the number of possible states per site. Extreme GC% composition affects the information that different *k-*mers have, and extreme deviations are relatively common in extremophilic organisms. Transposable elements and other forms of repetitive elements increase genome size, affect GC% locally, and reduce sequence complexity. Hybridization, heterokaryosis, and chimerism introduce 2 genotypic signals that might be quite divergent, which increases heterozygosity. Finally, contamination introduces undesired sequences with uneven composition, heterozygosity, and stoichiometry.

The main intrinsic factors that compromise the success of a genome assembly are the genome size, the sequence heterozygosity, the abundance of low-complexity regions (i.e., highly repetitive sequences), and the presence of high or uneven ploidy, contaminating sequences or extreme nucleotide compositions (Fig. 1).

Genome size impacts computational costs, as many assembly algorithms scale nonlinearly [1–3]. Heterozygosity implies the existence of allelic differences within an individual. Standard assembly algorithms have difficulty in differentiating between highly heterozygous regions and distinct but highly similar genomic regions [4, 5]. This, in turn, results in fragmented assemblies with inflated size compared to empirical measurements, as many of these regions appear duplicated [5], often in short scaffolds. This is particularly problematic in the case of individuals or a population that derived from sexual recombination between 2 or more distinct phylogenetic lineages (Fig. 1A), as the component subgenomes often develop structural rearrangements after the split of the 2 parental lineages. Similarly, repetitive or low-complexity genomic regions (Fig. 1B) are difficult to resolve without the aid of expensive experimental approaches (e.g., genetic maps, bacterial artificial chromosomes, long read sequencing techniques, or chromosome conformation capture), particularly when they span large genomic regions. Duplicated regions introduce multiple possible solutions to the process of scaffolding, increasing assembly fragmentation and computational costs [3, 4].

Similarly, ploidy deviations can greatly affect genome assembly. The first possible ploidy deviation is polyploidy (Fig. 1C), which is the presence of more than 2 chromosomes for the majority of the genome. Polyploidy is generally associated with genome heterozygosity, as it increases the number of possible states per site [6, 7]. For a diploid site, only 2 states are possible: heterozygous or homozygous, depending on whether the 2 alleles are different or equal, respectively. For a triploid, however, there are 2 possible heterozygotic states (e.g., AAB and ABB), and differentiating between them depends on relative frequencies. Allele frequency is affected by stochastic variation, specially if depth of sequencing is low. Aneuploidy (Fig. 1D) tends to cause the same problems as polyploidy in assemblies, albeit with the effect being limited only to the aneuploid regions. Because of this, genes present in chromosomes with ploidy higher than 2 will have a higher likelihood of being unannotated. Animal and plant genomics have traditionally considered aneuploidies as rare events, due to their deleterious effects on many of these organisms, especially during embryonic development [8]. This paradigm is clearly false for many fungal [9–12]) and protist [13, 14] lineages. Eukaryotic genomics has only recently started to focus on pangenomes [15–19], but aneuploidies might be an important confounding factor for these studies. For example, genes located in aneuploid regions are more likely to be missed in annotations, which can inflate estimations of presence/absence variation.

In syncytial organisms, such as filamentous fungi or slime molds, there is the possibility of coexistence of genetically different populations of nuclei within a cytoplasmic continuum, a condition known as heterokaryosis (Fig. 1E) [20–22]. Heterokaryosis is functionally similar to ploidy, although with some important differences. First, the relative proportions between heterozygous sites do not necessarily adjust to a simple fraction, as often one population is more abundant that the other. Second, since nuclei divide independently from each other, mitotic or meiotic recombination should be rare. This independence implies that any relative chromosomal rearrangements (i.e., duplications, deletions, translocations, and inversions) between the 2 nuclear populations, either pre- or postunion, would remain in nuclear populations for long periods of time. These rearrangements introduce the aforementioned complications in genome assemblies, and some of these might be difficult to differentiate from other chromosomal aberrations. A similar phenomenon is chimerism (Fig. 1F), in which the body of an organism is composed by 2 or more populations of genetically distinct cells. Certain lineages, especially colonial species, might arise by fusion of several genetically distinct individuals [23], but very little is known regarding the effect of chimerism in genome assemblies.

The presence of sequence contamination (Fig. 1G) can greatly compromise the quality of the genome assembly [24–28]. Extraneous sequences introduce noise, create chimeric contigs, and might introduce errors in *k*-mer estimations. Highly diverse contaminations (e.g., from the gut microbiota) introduce sequences with highly variable level of coverage, heterozygosity, and composition. On the other hand, highly abundant contaminants (e.g., symbiotic bacteria) are typically more homogeneous in all these parameters but might still form chimeric contigs and would indirectly reduce the depth of coverage in the main genome. Contaminations reducing the signal of the main genome are particularly problematic for single-cell sequencing projects [29, 30]. This is normally prevented by methodological means, but contaminating sequences are intrinsic for certain samples or even organisms, such as the case of symbiotic organisms (e.g., Lichens).

Finally, genomes with extreme compositions, typically very high or low GC content (GC%), can be difficult to assemble (Fig. 1H). For these genomes, the information contained by any AT positions is different from the information contained by a GC, as *k-*mers composed of the favored nucleotide pair will appear at higher frequencies. GC% has a well-documented effect on some sequencing technologies, most notably on the quality of Illumina reads [31, 32]. Fortunately, GC% is easy to measure from raw reads, and some genome assemblers include options specially adapted for these cases [33, 34]. Low GC% is typically associated with high abundance of low-complexity regions and transposable elements, but extreme GC% is also a hallmark of certain lineages, such as several groups of early diverging Fungi [35]. Despite their effects in genome analyses, GC% in eukaryotic genomes is often ignored. For example, neither NCBI nor Mycocosm report GC% in their assembly information statistics, unlike the Genome Online Database, which has a greater focus on prokaryotic sequences.

If the presence of the factors outlined above is anticipated, specific technical approaches—both experimental and computational— can be used. Contaminating DNA can be identified easily because sequencing coverage, nucleotide composition, and phylogenetic signal are usually different from the main genome, and several programs have been developed to identify contamination [24–28]. Ploidy can be estimated with cytogenetic techniques, which has been used for animals and plants since the 19th century. Unfortunately, cytogenetic techniques are time-consuming and difficult to interpret for some groups, such as the Fungi. Computational approaches exist to estimate composition and ploidy from sequencing reads [36–38]. Similarly, hybridization can be detected based on phenotypic traits (intermediate phenotypes and hybrid vigor). Again, this is not feasible for most microbial eukaryotes due to the lack of easily identifiable phenotypes. Genomes of hybrid organisms are heterozygous, and some genome assembly software has been designed to be able to handle this situation [5, 39, 40], but proper identification of hybrid lineages cannot be done without adequate population and phylogenetic analyses.

Thus, biological factors affecting genome assembly quality increase the overall costs of a project and require expertise that might not be available. Given the difficulty of performing analyses on low-quality assemblies, it is likely that published genomes are biased in favor of organisms with genomic characteristics that make them easier to work with. In contrast, genomic projects that choose organisms with nonstandard genomic architectures are more likely to suffer methodological obstacles that delay or even prevent analyses. Our inability to work around nonstandard genomic architectures distorts our perception of biological phenomena, relegating them to mere oddities.

## Results

### The Karyon toolkit

To aid in the identification of these noncanonical genomic architectures, we developed Karyon, a Python-based toolkit that assesses several parameters of sequencing data and their derived assemblies that are common indicators of different intrinsic genomic features leading to poor assemblies. Karyon comprises different modules that can be used independently or sequentially. Karyon is written in Python 3 and freely available to download as a Docker build or as a standalone project in https://github.com/Gabaldonlab/karyon.

**Figure 2:**
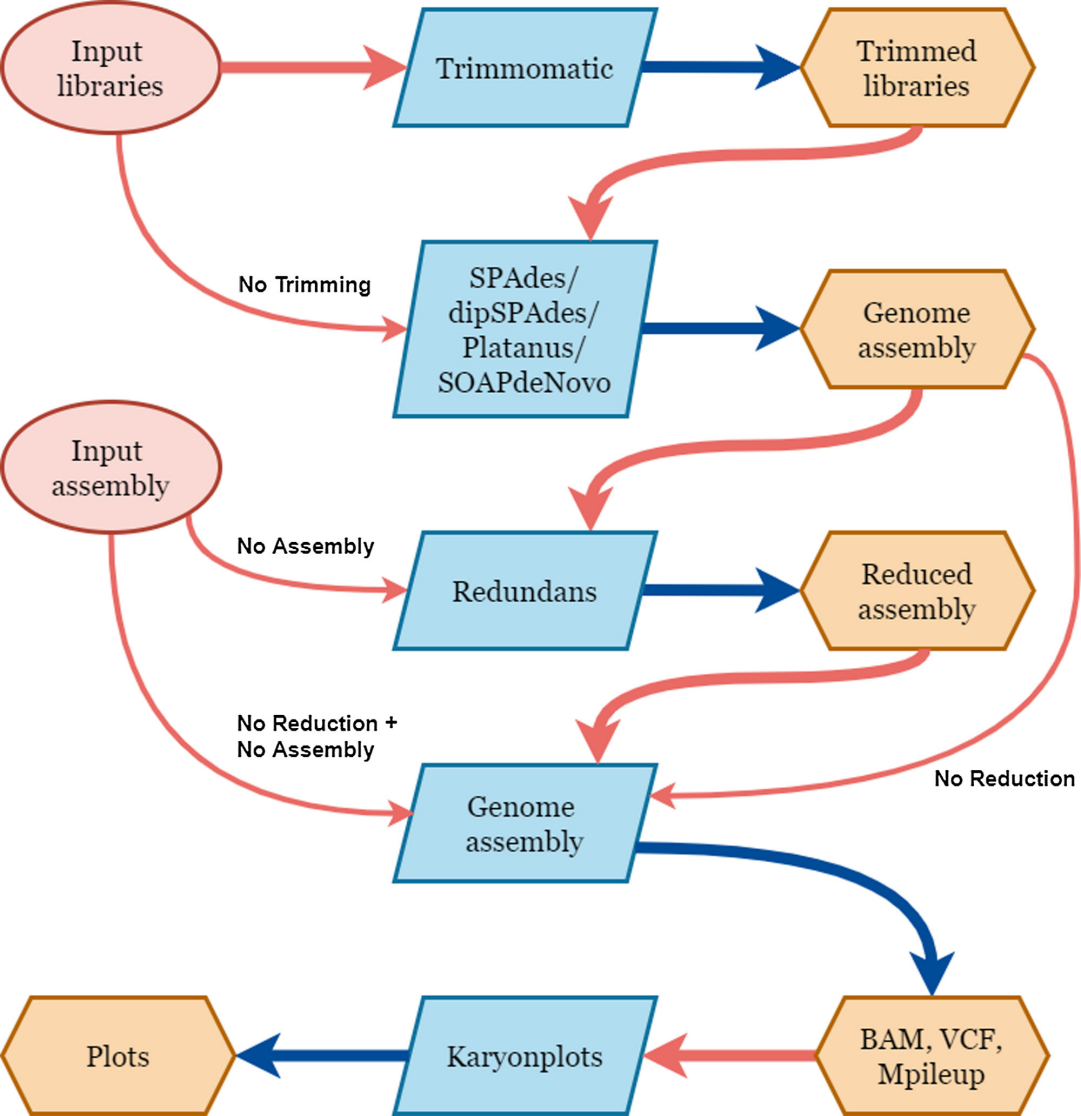
Karyon pipeline. Schematic representation of the steps and program used by Karyon. Red circles represent possible user inputs. Blue boxes represent software used for each step. Orange hexagons represent files generated by the software. Red arrows indicate input to a program, and blue arrows represent output of a program. Thicker red arrows represent the standard pipeline, while thinner red arrows represent the different options the user can select to skip some of the steps. These options appear next to the arrow.

Karyon integrates Trimmomatic [41] as an optional step to eliminate low-quality positions and adapters from sequencing reads. It then uses that input to generate a *de novo* assembly using SPAdes v3.9.0 [33], dipSPAdes v3.9.0 [40], Platanus v1.2.4 [39], or SOAPdenovo2 v2.04-r240 [42]. Karyon then uses the *de novo* assembly to generate a reduced assembly using Redundans [5]. Redundans is a pipeline that collapses assembly fragments with high similarity to create an artificial haploid genome assembly. This assembly is then used as reference to map the original sequencing reads using BWA-MEM [43] and generate a variant calling file with GATK v4.1.9.0 [44]. A battery of analyses is then performed on the sequencing libraries, the assemblies, and the maps of coverage and genetic variation to generate plots that will aid in the diagnosis of the genomic structure. Fig. 2 summarizes the pipeline.

Karyon uses the K-mer analysis toolkit (KAT) [36] to provide a *k*-mer (all possible sequences of length *k*) spectrum analysis as part of its report. From this analysis, it produces frequency histograms representing coverage versus *k*-mer counts. These plots inform on ploidy and heterozygosity of a genome. In a haploid genome, *k*-mers of enough size will appear either 1 or 0 times, with unique *k*-mers having an average coverage roughly equal to the average global depth of coverage. Deviations from these patterns suggest alternative architectures. For instance, the presence of 2 peaks in the *k*-mer plot typically indicates a genome that is totally or partially a nonhomozygous diploid. To complement these analyses and provide further information on the features of the genome, Karyon assesses scaffold length distributions, relationships between scaffold length and coverage, sliding-window analysis of coverage, and genetic variation, as well as allele frequency distributions per scaffold (Fig. 2). In addition, Karyon uses nQuire [38] to estimate the likelihood of different ploidy levels in sliding windows per scaffold. Karyon also incorporates BUSCO completeness analysis [45] with automatic taxonomic assignment. Altogether, the interpretation of these analyses can be used to detect polyploidies, aneuploidies, hybridizations, heterokaryosis, large segmental duplications, unusual DNA composition, or the presence of symbiont or contaminating sequences. Karyon generates a report file that summarizes the results of these analyses and raises some warning messages in case certain metrics are problematic, such as low BUSCO completeness, low percentage of mapped reads, or extreme GC% values.

**Figure 3:**
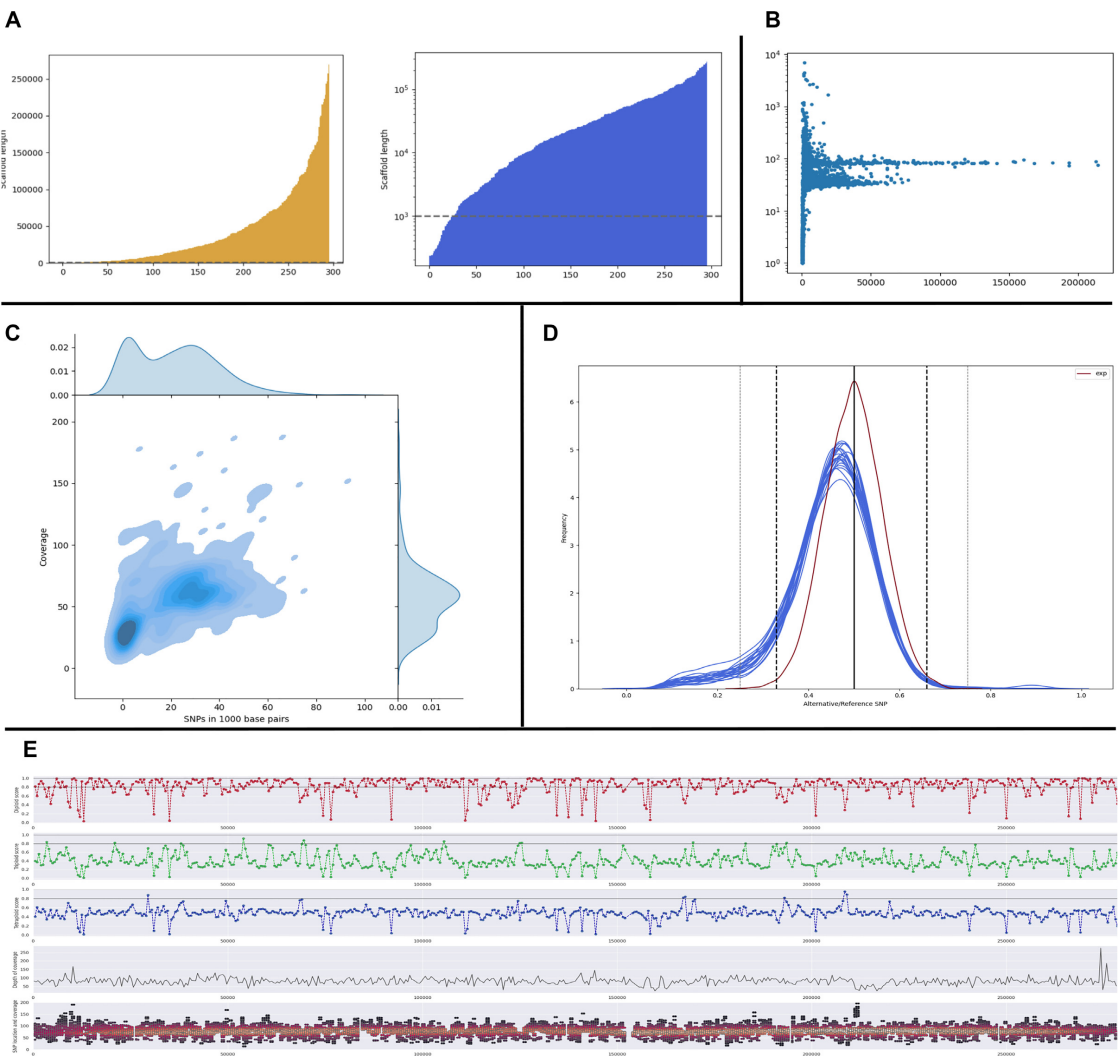
Summary of Karyon plots. (A) Scaffold length plot. (B) Scaffold length versus coverage plot. In the example, scaffolds form 2 populations with different coverage, which suggests aneuploidy behavior. (C) Variation versus coverage plot. In the example, the genome forms a clear population with low SNP density and approximately 30× of coverage and a second, more diffuse population with higher SNP density and approximately 60× coverage. This behavior suggests a mix of haploid and diploid regions. (D) Fair coin analysis. The red line represents a simulated distribution assuming perfect 50% distribution of reference and alternative SNPs. Each blue line represents the empirical distribution of reference versus alternative SNPs per scaffold, which in this case all follow a diploid distribution. (E) Per-scaffold nQuire plot. The plot represents nQuire-generated normalized values across sliding windows for a single scaffold. Most windows have a high diploid score, which suggests that this particular scaffold is diploid.

Karyon generates a series of original plots that aim to provide valuable information regarding the architecture of the problem assembly:

1. **Scaffold length plots** (Fig. 3A). These plots represent the distribution of scaffold length through a barplot, where each value represents a single scaffold versus its length, with all scaffolds sorted from shortest to longest. Karyon generates the results in linear and logarithmic distribution. Very short scaffolds (shorter than 1 Kbp) might introduce noise in the analyses and might be interesting to just filter them out.
2. **Scaffold versus coverage** (Fig. 3B). Karyon generates a scatterplot representing the average coverage versus length for each scaffold in the assembly. Quite often, short scaffolds have different coverage from most of the genome, which might be indicative of contamination or repetitive regions.
3. **Variation versus coverage plot** (Fig. 3C). This plot allows the user to observe overall patterns across the whole genome. It uses a kernel density estimation over a cloud of dots. Each dot represents the number of single-nucleotide polymorphisms (SNPs) (x-axis) versus the average coverage (y-axis) in a window of the genome, typically 1 Kb. Presence of more than 1 population of dots is indicative of genomes with dual behavior, such as aneuploidies or loss of heterozygosity.
4. **Fair coin plot** (Fig. 3D). This plot represents the proportion of alternative versus reference SNPs for the whole genome and for each individual scaffold. Vertical lines indicate expected frequencies of 0.5, 0.33, and 0.25, corresponding to ideal diploids, triploids, and tetraploids, respectively. An expected frequency is drawn, which is based on a per-site simulation of proportions assuming ideal 0.5 relative frequencies and random sampling equal to the coverage of the site. The plot is generated for the whole genome, as well as per scaffold.
5. **nQuire per scaffold plot** (Fig. 3E). Karyon will run nQuire across sliding windows of defined length (by default 1 Kbp) across different scaffolds. The plot for each scaffold contains 5 subplots. The first 3 represent the nQuire score for diploid, triploid, and tetraploid for that particular window. This allows the user to visualize patterns of aneuploidy per scaffold, especially with regards to diploid and triploid regions. The fourth and fifth subplots represent the location and coverage of SNPs across the scaffold. A color code is assigned to represent the density. Since nQuire requires information of SNPs, homozygous regions cannot be assessed and will appear as missing data.

Each of the steps is optional and can be controlled with flags in the main script. Additionally, the script uses a configuration file, which allows to define the options of each dependency program. This configuration file is automatically created during the installation and can be modified with any text editor. We encourage the user to make a copy of the original configuration file for future modification. Installation is fully automated, requiring no user input during the process.

### Genomic survey in the Mucorales (Fungi)

To showcase the use of Karyon, we undertook an analysis of deposited fungal genomes in the order Mucorales. Fungi are in a particularly privileged position to assess the impact of noncanonical genomic architectures in genome assemblies. Fungi generally have small and compact genomes and can be often cultured under axenic conditions. As a result, the amount of sequenced fungal genomes is now in the order of thousands, including multiple strains for many species. Even more, comprehensive efforts to obtain a balanced coverage of the existing fungal diversity are ongoing, such as the 1,000 fungal genomes [46] and the 1,000 yeast genomes initiatives [47–50]. Thus, fungi provide an excellent system to study the incidence of different genomic accidents in evolution [10, 51, 52]. Despite this, the quality of fungal genomes is often suboptimal, and databases are rife with highly fragmented assemblies. Genomic factors such as those discussed above might complicate genome assembly and be responsible for this observed fragmentation, at least partially. Considering this, we hypothesized that genome databases must contain a fraction of low-quality assemblies from fungal organisms that are caused by intrinsic genomic factors. If that is true, reanalysis of the raw data should lead us to describe novel genomic accidents and obtain a minimum estimate of their relative abundance.

We thus applied Karyon to a set of 35 publicly deposited genomes from the fungal order Mucorales. Our results suggest that nonstandard genomic organizations are not rare and that future studies on other groups are likely to uncover many new cases. We selected the order Mucorales because this group comprises several described examples of whole-genome duplication, both ancient and recent [53, 54]. Many sequenced members of the clade come from clinical samples, an environment that is known to promote the emergence of different genomic accidents [52, 55, 56]. Additionally, several represented species included 2 or more sequenced isolates, allowing to get a glimpse at their intraspecific diversity. We obtained 35 genome assemblies from 19 different Mucorales species deposited in GenBank between 1 January 2005 and 31 December 2015 (Table 1). For 4 of the species, dipSPADes was unable to generate an assembly.

**Table 1:** NCBI Assembly statistics for the analyzed strains. Strains with darker background possessed some property that was affecting assembly quality and was diagnosed using Karyon. Fragmentation in all remaining strains is attributed to low sequencing depth.

Species	NCBI genome size (Mbp)	NCBI number of scaffolds	GenBank accession	Genome size after Karyon (Mbp)	Number of scaffolds after Karyon	Diagnosis	Reference
*Rhizopus microsporus* ATCC 62 417	49.6	1,386	GCA_900000135.1	40.1	5,521	Contamination	[57]
*Rhizopus microsporus* CBS_344.29	49.2	1,554	GCA_000825725.1	32.1	3,037	Contamination	[57]
*Rhizopus microsporus* B9738	75.1	5,266	GCA_000697275.1	71.6	12,789	Misidentification	[58]
*Rhizopus microsporus* var. *rhizopodiformis* B7455	48.7	4,658	GCA_000738565.1	21.8	2,176	Contamination	[58]
*Rhizopus delemar* type I NRRL 21789	42.0	3,921	GCA_000697155.1	33.4	4,824	Unknown	[58]
*Rhizopus delemar* type II NRRL 21446	38.9	1,156	GCA_000738605.1	33.7	5,071	Unknown	[58]
*Rhizopus delemar* type II NRRL 21447	38.7	1,177	GCA_000738595.1	28.9	6,683	Unknown	[58]
*Rhizopus delemar* type II NRRL 21477	40.8	1,808	GCA_000738585.1	None	None	Unknown	[58]
*Rhizopus oryzae* 99–892	39.1	1,168	GCA_000697725.1	29.6	1,875	Low GC% (below 35%)	[58]
*Rhizopus oryzae* HUMC02	40.3	2,313	GCA_000697605.1	None	None	Unknown	[58]
*Rhizopus oryzae* B7407	43.3	4,683	GCA_000696915.1	34.7	3,720	Low GC% (below 35%)	[58]
*Rhizopus oryzae* type I NRRL 13440	43.4	5,022	GCA_000697075.1	None	None	Unknown	[58]
*Rhizopus oryzae* type I NRRL 18148	47.5	14,653	GCA_000697095.1	None	None	Unknown	[58]
*Rhizopus oryzae* type I NRRL 21396	42.8	4,445	GCA_000697115.1	34.2	4,115	Unknown	[58]
*Rhizopus oryzae* 99–133	41.5	4,317	GCA_000697135.1	27.2	1,332	Unknown	[58]
*Rhizopus oryzae* 97–1192	42.9	4,566	GCA_000697195.1	None	None	Unknown	[58]
*Rhizopus stolonifer* B9770	38	5,567	GCA_000697035.1	30.1	6,406	Unknown	[58]
*Mucor circinelloides* B8987	36.7	2,210	GCA_000696935.1	29.9	4,864	Unknown	[58]
*Mucor indicus* B7402	39.8	3,117	GCA_000697295.1	32.1	691	Unknown	[58]
*Mucor racemosus* B9645	65.5	6,360	GCA_000697255.1	46.0	4,444	Hemidiploid, low GC% (below 35%)	[58]
*Mucor velutinosus* B5328	35.9	2,411	GCA_000696895.1	28.2	2,743	Unknown	[58]
*Lichtheimia corymbifera* 008–049	36.6	1,629	GCA_000697175.1	42.8	3,575	Unknown	[58]
*Lichtheimia corymbifera* B2541	36.6	1,176	GCA_000697475.1	13.2	3,575	Unknown	[58]
*Lichtheimia ramosa* B5399	45.6	3,968	GCA_000738555.1	26.6	861	Aneuploid, hybrid	[58]
*Saksenaea oblongisporus* B3353	40.8	1,702	GCA_000697495.1	29.7	622	Unknown	[58]
*Saksenaea vasiformis* B4078	42.5	2,417	GCA_000697055.1	32.7	1,506	Unknown	[58]
*Cokeromyces recurvatus* B5483	29.3	2,637	GCA_000697235.1	26.6	5,213	Low GC% (below 35%)	[58]
*Syncephalastrum monosporum* B8922	29.6	1,284	GCA_000697355.1	24.1	5,271	Unknown	[58]
*Syncephalastrum racemosum* B6101	29.6	1,035	GCA_000696955.1	23.3	311	Unknown	[58]
*Cunninghamella elegans* B9769	31.7	1,380	GCA_000697015.1	30.8	5,465	Low GC% (below 35%)	[58]
*Apophysomyces elegans* B7760	38.5	1,528	GCA_000696995.1	29.3	1,293	Unknown	[58]
*Apophysomyces trapeziformis* B9324	35.8	1,400	GCA_000696975.1	30.1	898	Unknown	[58]
*Thermomucor indicae-seudaticae* HACC 243	29.6	1,958	GCA_000787465.1	25.7	4,118	Unknown	Busk et al., unpublished. Genome submitted in 2014.
*Parasitella parasitica* CBS 412.66 isolate NGI315 ade- mutant	44.9	15,637	GCA_000938895.1	23.5	3,295	Unknown	[59]

Karyon was run using the complete default pipeline. Most of the analyzed genomes (27, 79.4%) presented very low levels of heterozygosity and a relatively homogeneous coverage across the genome, suggesting that those strains are haploid or, if presenting higher ploidy, extremely homozygous. Fragmentation in these cases might be caused by insufficient coverage, presence of repetitive regions, or some other methodological constraints. However, our pipeline uncovered cases that produced anomalous results in the different Karyon tests. Many zygomycetous fungi exhibit low or very low GC%. In our dataset, 5 species showed GC% below our threshold of 35%, with several others approximating that value. Additionally, some of the analyzed genomes show signs of ploidy anomalies or contamination. Below we describe these cases and propose a plausible scenario to explain each of the obtained results based on the data obtained from the Karyon pipeline.

### *Rhizopus microsporus* species complex

**Figure 4:**
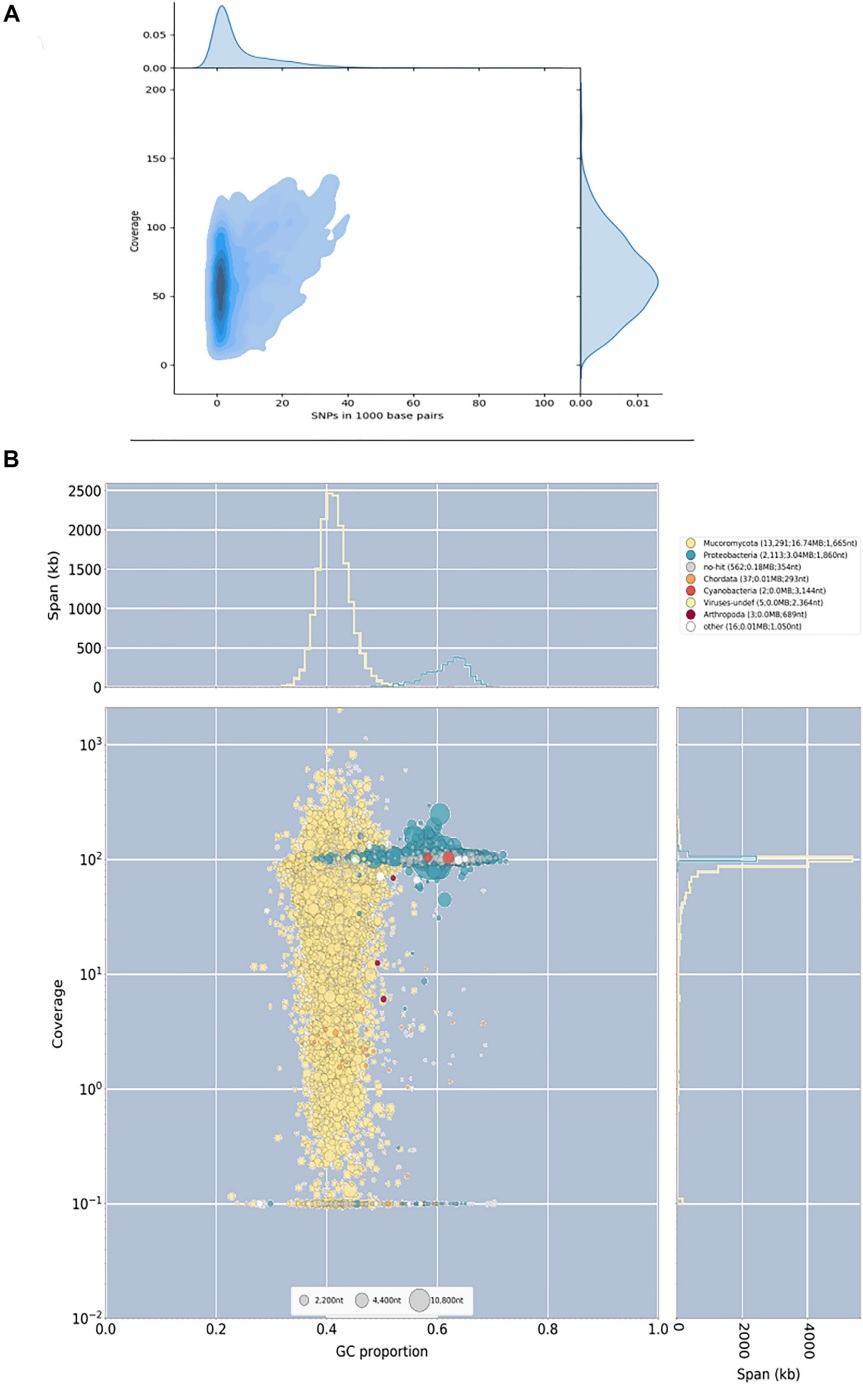
Analysis of *Rhizopus microsporus* ATCC62417. (A) Variation versus coverage plot reveals the existence of a highly variable portion of the genome that presents variable heterozygosity levels. (B) BlobTools analyses suggest that the genome presents a considerable portion of contaminating sequences. Coverage of the sequences assigned to bacteria is very low when the analyses are performed with other libraries (data not shown), which proves that the conflicting signal observed in this sample has its origin in a contaminated sequencing library. Results for *R. microsporus* CBS344.5 and var. *rhizopodiformis* B7455 show similar patterns of contamination (data not shown).

At the time of this study, the NCBI database had deposited sequences for 8 *Rhizopus microsporus* strains. Interestingly, 3 of them presented a genome size estimated around 25 Mbp, 4 had a genome size close to 50 Mbp, and 1 presented a genome size of 75 Mbp. Only the 3 strains with a genome size of 25 Mbp had sufficiently good assemblies considering they were based on short reads, with a scaffold number below 1,000, and thus were not selected for further analyses. Additionally, the raw libraries for one of the strains presenting a 50-Mbp genome assembly size (*R. microsporus* var. *chinensis* CCTCC M201021) were not publicly available and thus could not be part of the survey. For the remaining 3 strains with genome size close to 50 Mbp (ATCC62417, CBS344.29, and var *rhizopodiformis* B7455), our *de novo* assembly pipeline recovered a genome size of approximately 40 Mb, which is smaller than the assemblies deposited in NCBI (Table 1). The heterozygosity distribution in these assemblies shows that most of the genome presented a relatively uniform behavior with low heterozygosity. In all 3 cases, though, a considerable proportion of the genome appears with a highly variable coverage and increased heterozygosity (Fig. 4). For these 3 strains, BlobTools [25] shows widespread bacterial contamination (Fig. 4B), and thus we conclude that contamination might be responsible for the observed assembly fragmentation.

**Figure 5:**
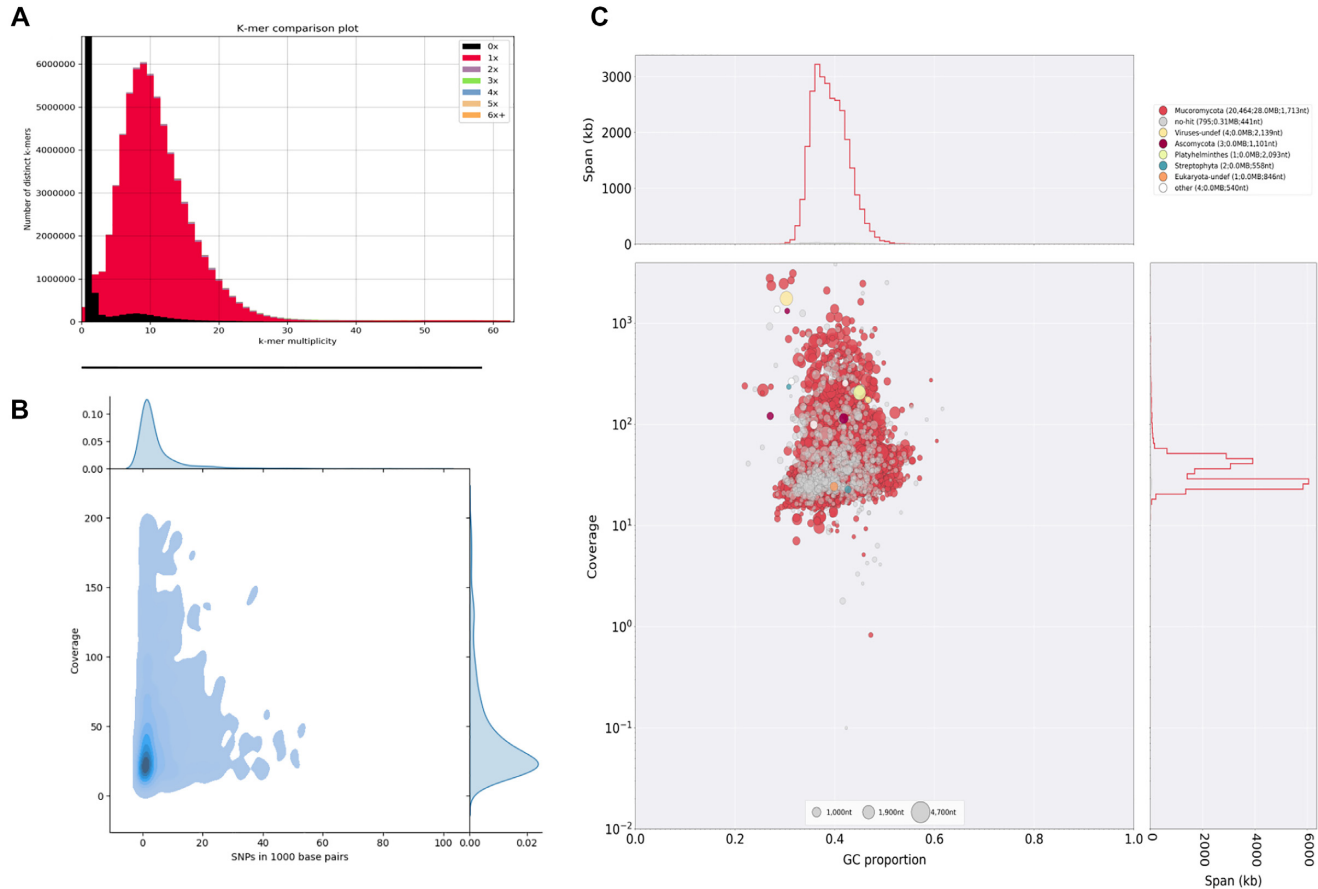
Analysis of *Rhizopus microsporus* B9738. (A) KAT *k*-mer plot shows very low genome compaction (black area), suggestive of a haploid genome. (B) Variation versus coverage plot reveals a single main behavior for the genome with regards to its SNP density and coverage. (C) BlobTools analysis shows no sign of widespread contamination that might be inflating the genome.

**Figure 6:**
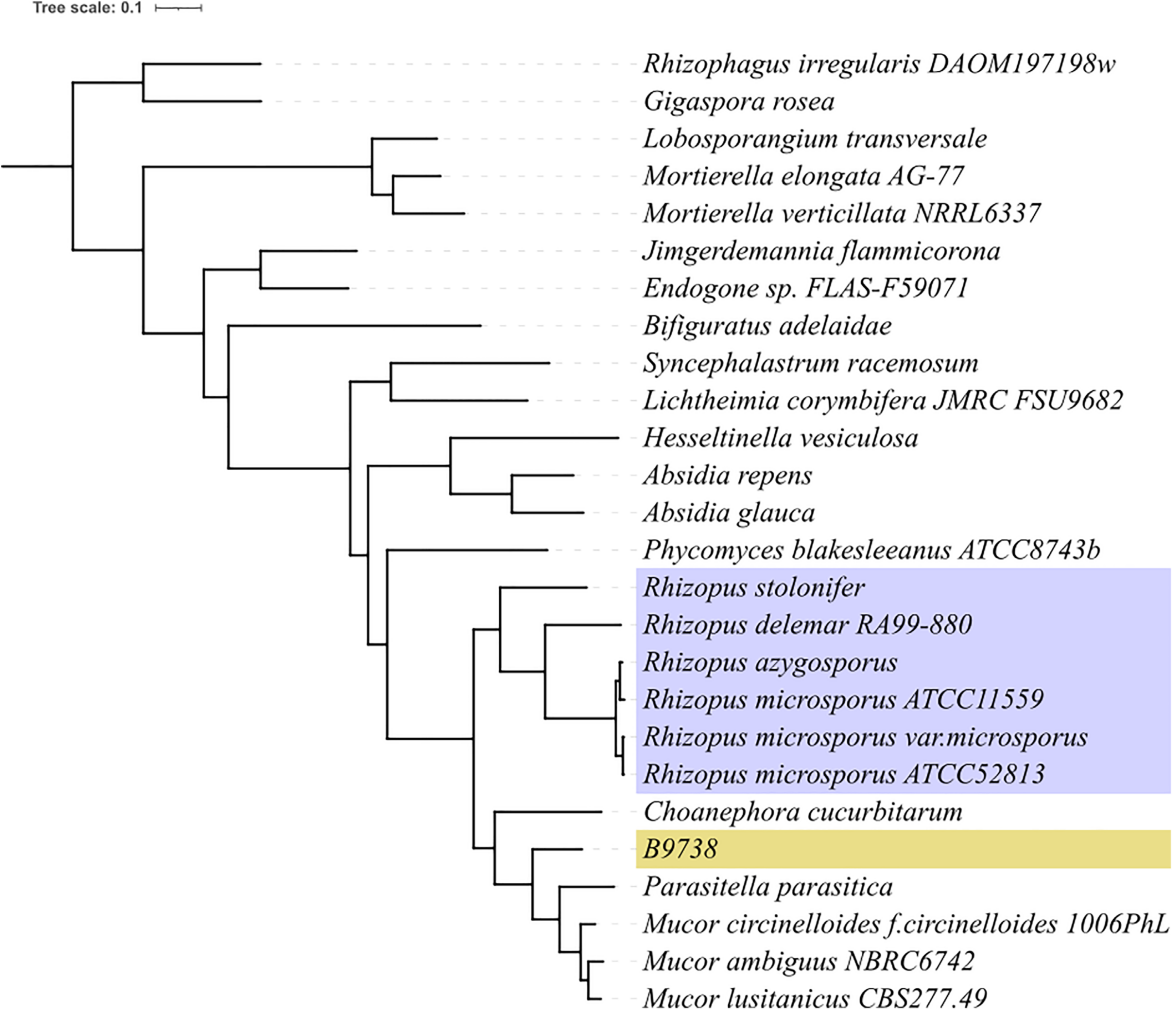
Phylogenetic tree of *Rhizopus microsporus* B9738. Phylogenetic tree inferred from OrthoFinder. The *Rhizopus microsporus* species complex is marked in blue. The problematic strain, B9738, is marked in yellow.

The remaining strain, B9738, showed a surprisingly large genome size in both the assembly deposited in NCBI (75 Mbp) and the one reconstructed here (71 Mbp). The genome of *R. microsporus* B9738 presents an extremely low level of heterozygosity and a very homogeneous coverage. The *k*-mer spectrum also shows just 1 very clear peak. All in all, all this suggests that B9738 is haploid (or a highly homozygous diploid), despite presenting a 3-fold increase in genome size as compared to other strains of the same species (Fig. 5). Augustus gene prediction returned a total of 21,300 gene models, which is an unusually large number for a filamentous fungus. As a reference, the 7 genomes in the Rhizopodaceae, to which *Rhizopus* belongs, available in Mycocosm range from 25 to 46 Mbp and from 10,781 to 17,676 annotated genes. Contamination analysis does not suggest the presence of widespread contamination that could explain such an overinflated genome (Fig. 5). For this reason, we suggest that B9738 might be a misidentified strain that does not belong to the *R. microsporus* species complex. Indeed, phylogenomic analyses recover B9738 as sister to a clade containing *Mucor* and *Parasitella*, rather than allied with the rest of the *R. microsporus* species clade (Fig. 6), thus supporting a misidentification. It is noteworthy that no sequenced species of either *Mucor* or *Parasitella* have genomes above 49 Mbp or with more than 15,000 genes, at least from the available genomes in Mycocosm.

### *Mucor racemosus* B9645

**Figure 7:**
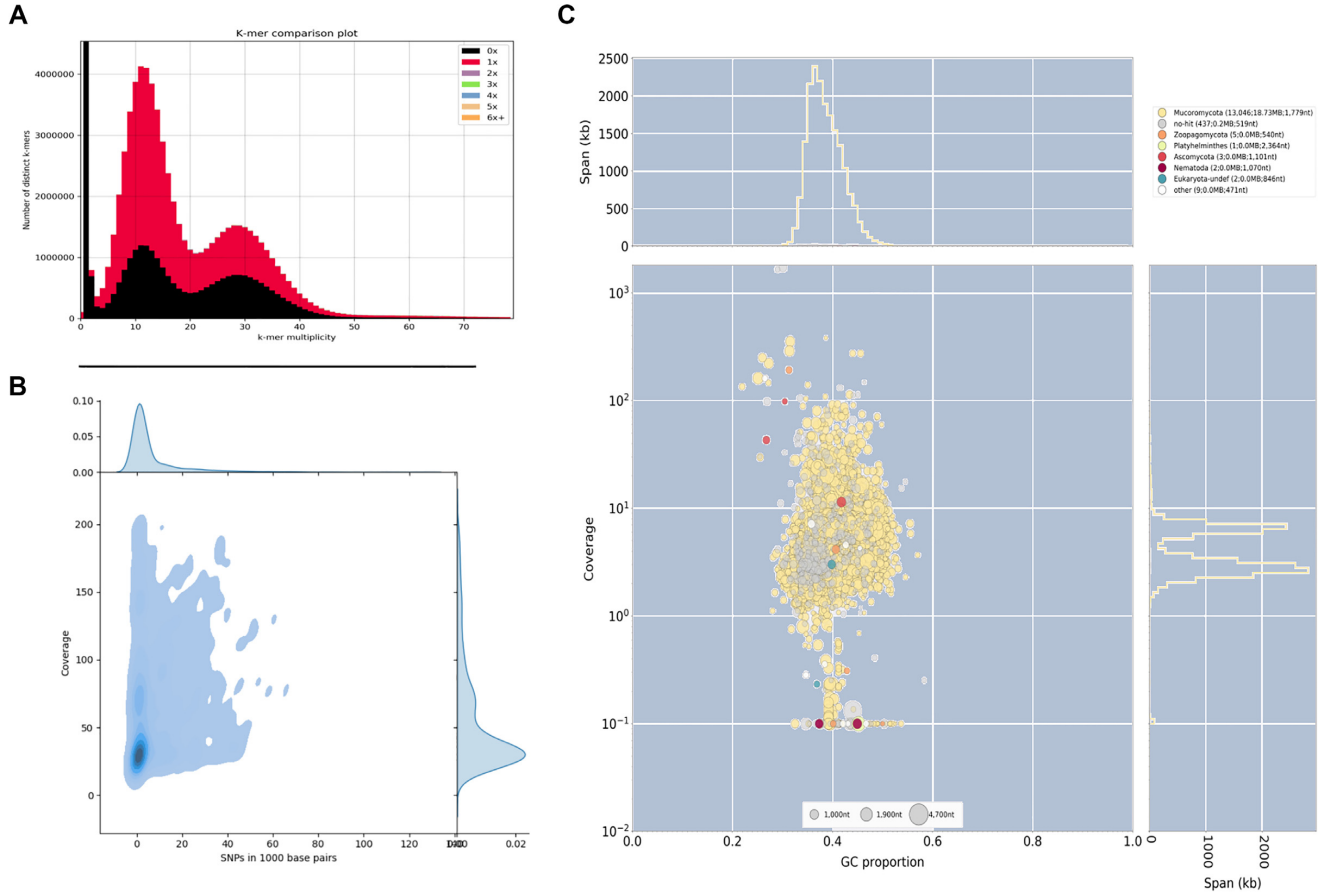
Analysis of *Mucor racemosus* B9645. (A) KAT *k*-mer plot shows 2 peaks of coverage considerably affected by genome reduction (black area), suggestive of a highly heterozygous diploid genome. (B) Variation versus coverage plot reveals a bimodal behavior for the genome with regards to its coverage, but both peaks appear with very low SNP density. (C) BlobTools analysis shows no sign of widespread contamination that might be inflating the genome.

Analyses on *Mucor racemosus* B9645 depicted a genome with a dual behavior. The distribution of heterozygosity and coverage showed 2 peaks with very low heterozygosity but with different coverage (Fig. 7B). This was further confirmed by the *k*-mer spectrum analysis, which revealed 2 clear peaks (Fig. 7A). The genome available in NCBI is 65.5 Mbp long, noticeably larger than the 45.9 Mbp we recovered in our analyses (Table 1). The reduction step of Redundans cannot explain this difference, as the assembly size prior to this step is already 46.8 Mbp, very close to the final result. Our analyses suggest that contaminating sequences are very minor and do not explain the observed pattern (Fig. 7). We hypothesize that *M. racemosus* B9645 is a hemidiploid, which presents a portion of its genome in a haploid state and the other portion in a highly homozygous diploid state. Due to the low heterozygosity exhibited by this strain, the observed genome architecture might have arisen by either autopolyploidization followed by chromosome loss or by chromosomal duplications. Additionally, GC% for this species was only 32.6%.

### *Lichtheimia ramosa* B5399

**Figure 8:**
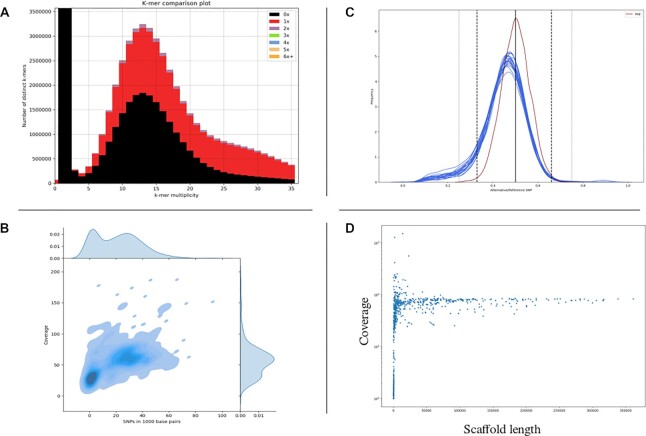
Analysis of *Lichtheimia ramosa* B5399. (A) KAT *k*-mer plot shows 1 peak with considerable genome compaction (black area) suggestive of a diploid genome. (B) Variation versus coverage plot reveals a unimodal behavior for the genome with regards to its coverage, presenting a widespread heterozygosity of approximately 3% (maximum density around 30 SNP/Kbp). (C) Alternative allele frequency shows that all scaffolds present a behavior very similar to the ideal diploid. (D) Scaffold length plot shows that, except for a group of very low-coverage scaffolds, the entire genome presents a uniform coverage.

The Karyon assembly for this genome was only 26.6 Mbp, much smaller than the NCBI assembly (45.6 Mpb long, Table 1). Unlike other genomes, our assembly presented a considerable improved quality, going from 3,968 scaffolds and N50 of 33,650 in the NCBI assembly to 861 scaffolds and N50 of 133,635 in our own assembly. *L. ramosa* presents a heterozygosity level around 3% in its diploid peak (Fig. 8). All considered, we propose that *L. ramosa* B5399 is a mix of haploid and diploid with high heterozygosity, likely resulting from mating between 2 distantly related strains followed by genomic aneuploidization.

## Methods

### Sequencing data

We downloaded raw data from libraries deposited at the Short Read Archive (SRA)⁠ of those species in the Mucorales with a highly fragmented assembly (>1,000 scaffolds), which included at least 1 paired-end Illumina library larger than 1 Gb after quality filtering (Table 1), to ensure at least a decent coverage. Since most of our genomes have typical assembly sizes around 40 Mbp, this measure ensures a bare minimum average coverage of 20. All available sequencing libraries were used for all the analyses.

### *De novo* gene annotation

We used Augustus v3.1.0. [60] to obtain a *de novo* gene prediction using the *Rhizopus oryzae* generalized hidden Markov model included in the default installation of Augustus.

### Contamination detection

For each of the conflictive assemblies, we generated an Augustus prediction. Then, we used Blastp [61] to query the whole proteome against Uniref100 [62]. Since the genomes come from public databases, their own proteins should appear as hits, and thus we retrieved the 10 best hits. We have used these hits to assign a taxonomic profile. Additionally, we have used the predicted Augustus CDS to map sequencing reads with GATK. With both the taxonomic profile and the variant calling file, we have run BlobTools [25] to identify the presence of widespread contamination in the sequencing libraries.

### Phylogenomic analyses

In order to identify the phylogenetic position of *R. microsporus* B9738, we used the Augustus gene prediction and the proteome of 24 other zygomycetes to run OrthoFinder v.2.3.3 [63] with the flags -S blast and -m msa.

## Discussion

As genome sequencing has moved away from model organisms, it has become apparent that many possible genomic architectures are possible, and many do exist in a wide range of organisms. Most of these genomic accidents are difficult to identify from sequencing data alone. As far as we know, Karyon is the first software developed with the intention of performing reference-free analyses for the presence of a wide array of genomic factors affecting the quality of *de novo* genome assembly. We have designed this software to be easy to install and use, with the possibility of installation from both GitHub and Docker.

Despite the success in the implemented strategy, we consider our software has several limitations. Karyon requires an assembly step and variant calling protocol, for which some default options are included. However, the included programs might not suit every need. For example, extremely large genomes might require alternative assemblers that are not included in our pipeline, or some users might prefer a different set of programs for the variant calling protocol. For those cases, Karyon can still be used as independent steps (Fig. 2). Karyon is designed to work without any preexisting data, which limits the information it can predict. Comparing different genome assemblies, especially if at least one of them has good quality, can help detect many of these alternative genomic architectures and some others that are outside the capabilities of Karyon. If other reference genomes are available, tools like QUAST [64] can generate similar analyses to Karyon with higher accuracy and speed.

Despite the increasing use of long-read technologies for assembly purposes, a large amount of genome assemblies available in public databases have been generated exclusively from short reads. As of October 2021, NCBI SRA contains 173,087 DNA libraries for Fungi, of which 157,144 are Illumina short reads, and only 6,163 are long reads (4,700 PacBio and 1,463 Nanopore). At this moment, the pipeline assumes the use of at least 1 Illumina paired-end sequencing library. Because of this, we recommend the use of other genome assemblers if other sequencing technologies (i.e., Nanopore or PacBio long reads) are to be used, and the same goes for variant calling protocols.

We provided a practical example of the usage of Karyon on a publicly available set of fungal genomes from the order Mucorales. While most analyzed assemblies show no sign of any of the considered biological conditions, we were able to effectively find underlying nonstandard genomic architectures that had been previously unnoticed in these assemblies. These results suggest that many authors do not take into consideration these kinds of genomic accidents, which in turn greatly hampers the results that might be obtained from them.

How common are these nonstandard genomic architectures? Our results suggest that they might be quite abundant, although so far they are restricted to a limited selection of species within a narrow clade of Fungi. As such, these genomic anomalies might, or might not, be common in other lineages. However, we consider that there are 3 important arguments in favor for considering our dataset an underestimation of the abundance of unorthodox fungal genomes, even within the limited taxonomic range we have selected. The first one is the fact that fungal biomass used for DNA extraction and subsequent sequencing typically comes from cultures. This implies an important ecological step in which the fungus grows at optimal speed and in the absence of most stressors. Aneuploidies, polyploidies, and other similar genomic rearrangements are common in the presence of stressors [9, 10, 52, 65] but seem to be outcompeted by euploid cells under optimal growth conditions [24, 66, 67]. Hence, isolates growing in rich medium will be selected to lose most chromosomal aberrations they might present. Analogously, many of these chromosomal aberrations might exist in nature but are unable to grow on optimal medium. The advance of environmental sequencing and single-cell-based technologies might cast some light in this matter in coming years. Supporting this argument, Ahrendt et al. [68] sequenced several environmental isolates of zoosporic and zygomycetous microfungi using these techniques and found several aneuploids and polyploids. The frequency of unconventional genomic architectures is very likely lineage dependent. While some of these are well known, such as the dikaryotic phase in Agaricomycetes or the macro- and micronuclei of ciliates, strange genomic architectures might be common in more obscure lineages. This not only represents a yet-to-know facet of the biology of these organisms but could also potentially complicate their study. The third factor to consider is purely human. The datasets we have analyzed were uploaded by researchers who considered they were good enough to be uploaded to a public repository. Thus, it is to be expected that many more low-quality assemblies would have never been never deposited and sit forgotten in the disks of laboratory computers, if not discarded completely.

Even if we consider these possible biases as negligible, our results recover a significant fraction of publicly available genomes with unorthodox genomic configurations. These have been correlated in many fungal groups with adaptation to novel environments [69–71], resistance to antifungals [72, 73], pathogenic capabilities toward both animals [55, 74–76] and plants [77, 78], and adaptation to industrial settings [47, 79–83]. Beyond that, contamination in sequencing libraries is a problem that can affect any assembly project and might mislead downstream inferences if left unaddressed. Validation of published results goes far beyond the interest of discovering overlooked findings. Comparative genomic studies are limited in their scope and reliability by the quality of assembly and annotation of the genomes, factors that can be greatly compromised by these biological factors. Comparative studies commonly require the use of flagship genomes that represent a given taxon. Often, this generates a chronology of comparisons versus the reference that shapes the perspective on the group. As such, artifacts and errors in strategic genome assemblies, such as reference strains or strains in groups with few represented species, might have a domino effect, impacting future studies. Long-read sequencing technologies, which are increasingly being used for genome assembly projects, hold the promise of providing much more information that could be used to resolve many of these unorthodox genomic architectures. However, these approaches require novel computational approaches to fully employ their potential.

## Availability of supporting source code and requirements

Project name: Karyon

Project homepage: https://github.com/Gabaldonlab/karyon

Operating system(s): e.g., Linux, any with the Docker image

Programming language: Python, Bash

Other requirements: Check the installation

License: GNU GPL v3

RRID: SCR_022544

biotools ID: karyon

## Data Availability

All data used for this study were downloaded from NCBI SRA. Table 1 contains accession numbers for all the data. An archival copy of the code and test data is available via the *GigaScience* database GigaDB [84].

## Abbreviations

BUSCO: Benchmarking Universal Single-Copy Orthologs; NCBI: The National Center for Biotechnology Information; SNP: single-nucleotide polymorphism; SRA: Short Read Archive.

## Competing Interests

The authors state that they have no conflicts of interests.

## Funding

Supported by the Spanish Ministry of Science and Innovation (grant PGC2018-099921-B-I00), cofounded by the European Regional Development Fund (ERDF); the Catalan Research Agency (AGAUR), SGR423; the European Union's Horizon 2020 research and innovation program (ERC-2016–724173); the Gordon and Betty Moore Foundation (grant GBMF9742); and the Instituto de Salud Carlos III (IMPACT grant IMP/00019 and CIBERINFEC CB21/13/00061-ISCIII-SGEFI/ERDF).

## Supplementary Material

giac088_GIGA-D-21-00155_Original_Submission

giac088_GIGA-D-21-00155_Revision_1

giac088_GIGA-D-21-00155_Revision_2

giac088_GIGA-D-21-00155_Revision_3

giac088_GIGA-D-21-00155_Revision_4

giac088_Response_to_Reviewer_Comments_Original_Submission

giac088_Response_to_Reviewer_Comments_Revision_1

giac088_Response_to_Reviewer_Comments_Revision_2

giac088_Response_to_Reviewer_Comments_Revision_3

giac088_Reviewer_1_Report_Original_Submission Zhong Wang -- 6/29/2021 Reviewed

giac088_Reviewer_1_Report_Revision_1 Zhong Wang -- 12/2/2021 Reviewed

giac088_Reviewer_2_Report_Original_Submission Michael F. Seidl -- 7/4/2021 Reviewed

giac088_Reviewer_2_Report_Revision_1 Michael F. Seidl -- 11/29/2021 Reviewed

giac088_Reviewer_3_Report_Original_Submission Kamil S. Jaron -- 7/10/2021 Reviewed

giac088_Reviewer_3_Report_Revision_1 Kamil S. Jaron -- 1/10/202 Reviewed

giac088_Reviewer_3_Report_Revision_2 Kamil S. Jaron -- 5/17/2022 Reviewed

## References

[1] Simpson JT, Pop M. The theory and practice of genome sequence assembly. Annu Rev Genomics Hum Genet. 2015;16(1):153–72. 25939056 10.1146/annurev-genom-090314-050032

[2] Wajid B, Serpedin E. Review of general algorithmic features for genome assemblers for next generation sequencers. Genomics Proteomics Bioinformatics. 2012;10(2):58–73. 22768980 10.1016/j.gpb.2012.05.006 PMC5054208

[3] Wajid B, Sohail MU, Ekti AR, et al. The A, C, G, and T of genome assembly. Biomed Res Int. 2016;2016:1. 10.1155/2016/6329217 PMC4877455 27247941

[4] Hirsch CN, Robin Buell C. Tapping the promise of genomics in species with complex, nonmodel genomes. Annu Rev Plant Biol. 2013;64(1):89–110. 23451780 10.1146/annurev-arplant-050312-120237

[5] Pryszcz LP, Gabaldón T. Redundans: An assembly pipeline for highly heterozygous genomes. Nucleic Acids Res. 2016;8(44):1–16. 10.1093/nar/gkw294 PMC4937319 27131372

[6] Aguiar D, Istrail S. Haplotype assembly in polyploid genomes and identical by descent shared tracts. Bioinformatics. 2013;29(13):i352–60. 23813004 10.1093/bioinformatics/btt213 PMC3694639

[7] Bonizzoni P, Dondi R, Klau GW, et al. On the minimum error correction problem for haplotype assembly in diploid and polyploid genomes. J Comput Biol. 2016;23(9):718–36. 27280382 10.1089/cmb.2015.0220

[8] Torres EM, Williams BR, Amon A. Aneuploidy: cells losing their balance. Genetics. 2008;179(2):737–46. 18558649 10.1534/genetics.108.090878 PMC2429870

[9] Anderson CA, Roberts S, Zhang H, et al. Ploidy variation in multinucleate cells changes under stress. Mol Biol Cell. 2015;26(6):1129–40. 25631818 10.1091/mbc.E14-09-1375 PMC4357512

[10] Berman J, Blutraich Wertheimer N, Stone N. Ploidy dynamics and evolvability in fungi. Phil Trans R Soc London B Biol Sci. 2016;371(20150461):1–11. 10.1098/rstb.2015.0461 PMC5095540 28080987

[11] Mehrabi R, Mirzadi Gohari A, Kema GHJ. Karyotype variability in plant-pathogenic fungi. Annu Rev Phytopathol. 2017;55(1):483–503. 28777924 10.1146/annurev-phyto-080615-095928

[12] Kumaran R, Yang SY, Leu JY. Characterization of chromosome stability in diploid, polyploid and hybrid yeast cells. PLoS One. 2013;8(7):e68094. 23874507 10.1371/journal.pone.0068094 PMC3707968

[13] Mannaert A, Downing T, Imamura H, et al. Adaptive mechanisms in pathogens: universal aneuploidy in Leishmania. Trends Parasitol. 2012;28(9):370–6. 22789456 10.1016/j.pt.2012.06.003

[14] Tůmová P, Uzlíková M, Jurczyk T, et al. Constitutive aneuploidy and genomic instability in the single-celled eukaryote *Giardia intestinalis*. MicrobiologyOpen. 2016;5(4):560–74. 27004936 10.1002/mbo3.351 PMC4985590

[15] Gerdol M, Moreira R, Cruz F, et al. Massive gene presence-absence variation shapes an open pan-genome in the Mediterranean mussel. Genome Biol. 2020;21(1):275. 33168033 10.1186/s13059-020-02180-3 PMC7653742

[16] Golicz AA, Batley J, Edwards D. Towards plant pangenomics. Plant Biotechnol J. 2016;14(4):1099–105. 26593040 10.1111/pbi.12499 PMC11388911

[17] McCarthy CGP, Fitzpatrick DA. Pan-genome analyses of model fungal species. Microbial Genomics. 2019;5(2):1–23. 10.1099/mgen.0.000243 PMC6421352 30714895

[18] Naranjo-Ortiz MA, Gabaldón T. Fungal evolution: cellular, genomic and metabolic complexity. Biol Rev. 2020;95(5):1198–232.. April, brv.12605. 32301582 10.1111/brv.12605 PMC7539958

[19] Sibbald SJ, Eme L, Archibald JM, et al. Lateral gene transfer mechanisms and pan-genomes in eukaryotes. Trends Parasitol. 2020;36(11):927–41. 32828660 10.1016/j.pt.2020.07.014

[20] James TY, Stenlid J, Olson Å, et al. Evolutionary significance of imbalanced nuclear ratios within heterokaryons of the Basidiomycete fungus *Heterobasidion parviporum*. Evolution. 2008;62(9):2279–96. 18637961 10.1111/j.1558-5646.2008.00462.x

[21] Maheshwari R . Nuclear behavior in fungal hyphae. FEMS Microbiol Lett. 2005;249(1):7–14. 16002240 10.1016/j.femsle.2005.06.031

[22] Strom NB, Bushley KE. Two genomes are better than one: history, genetics, and biotechnological applications of fungal heterokaryons. Fungal Biol Biotechnol. 2016;3(4):1–14. 28955463 10.1186/s40694-016-0022-x PMC5611628

[23] Blanquer A, Uriz M-J. ‘Living together apart’: the hidden genetic diversity of sponge populations. Mol Biol Evol. 2011;28(9):2435–8. 21498599 10.1093/molbev/msr096

[24] Kumar S, Jones M, Koutsovoulos G, et al. Blobology: exploring raw genome data for contaminants, symbionts and parasites using taxon-annotated GC-coverage plots. Front Genet. 2013;4:237. 24348509 10.3389/fgene.2013.00237 PMC3843372

[25] Laetsch DR, Blaxter ML. BlobTools: interrogation of genome assemblies. F1000Research. 2017;6:1287.

[26] Lu J, Salzberg SL. Removing contaminants from databases of draft genomes. PLoS Comput Biol. 2018;14(6):e1006277. 29939994 10.1371/journal.pcbi.1006277 PMC6034898

[27] Schmieder R, Edwards R. Fast identification and removal of sequence contamination from genomic and metagenomic datasets. PLoS One. 2011;6(3):e17288. 21408061 10.1371/journal.pone.0017288 PMC3052304

[28] Trivedi UH, Cézard T, Bridgett S, et al. Quality control of next-generation sequencing data without a reference. Front Genet. 2014;5:111. 24834071 10.3389/fgene.2014.00111 PMC4018527

[29] Gawad C, Koh W, Quake SR. Single-cell genome sequencing: current state of the science. Nat Rev Genet. 2016;17(3):175–88. 26806412 10.1038/nrg.2015.16

[30] Huang L, Ma F, Chapman A, et al. Single-cell whole-genome amplification and sequencing: methodology and applications. Annu Rev Genomics Hum Genet. 2015;16:79–102. 26077818 10.1146/annurev-genom-090413-025352

[31] Benjamini Y, Speed TP. Summarizing and correcting the GC content bias in high-throughput sequencing. Nucleic Acids Res. 2012;40(10):1–14. 22323520 10.1093/nar/gks001 PMC3378858

[32] Ross MG, Russ C, Costello M, et al. Characterizing and measuring bias in sequence data. Genome Biol. 2013;14(5):R51. 23718773 10.1186/gb-2013-14-5-r51 PMC4053816

[33] Bankevich A, Nurk S, Antipov D, et al. SPAdes: a new genome assembly algorithm and its applications to single-cell sequencing. J Comput Biol. 2012;19(5):455–77. 22506599 10.1089/cmb.2012.0021 PMC3342519

[34] Scott D, Ely B. Comparison of genome sequencing technology and assembly methods for the analysis of a GC-rich bacterial genome. Curr Microbiol. 2014;70(3):338–44. 25377284 10.1007/s00284-014-0721-6 PMC4318750

[35] Naranjo-Ortiz MA, Gabaldón T. Fungal evolution: diversity, taxonomy and phylogeny of the fungi. Biol Rev. 2019;94(6):2101–37. 31659870 10.1111/brv.12550 PMC6899921

[36] Mapleson D, Accinelli GG, Kettleborough G, et al. KAT: a k-mer analysis toolkit to quality control NGS datasets and genome assemblies. Bioinformatics. 2016;33(4):574–76. 10.1093/bioinformatics/btw663 PMC5408915 27797770

[37] Margarido GRA, Heckerman D, Myers EW, et al. ConPADE: genome assembly ploidy estimation from next-generation sequencing data. PLoS Comput Biol. 2015;11(4):e1004229. 25880203 10.1371/journal.pcbi.1004229 PMC4400156

[38] Weiß CL, Pais M, Cano LM, et al. nQuire: a statistical framework for ploidy estimation using next generation sequencing. BMC Bioinf. 2018;19(1):122. 10.1186/s12859-018-2128-z PMC5885312 29618319

[39] Kajitani R, Toshimoto K, Noguchi H, et al. Efficient de novo assembly of highly heterozygous genomes from whole-genome Shotgun short reads. Genome Res. 2014;24(8):1384–95. 24755901 10.1101/gr.170720.113 PMC4120091

[40] Safonova Y, Bankevich A, Pevzner PA. DipSPAdes: assembler for highly polymorphic diploid genomes. J Comput Biol. 2015;22(6):528–45. 25734602 10.1089/cmb.2014.0153 PMC4449708

[41] Bolger AM, Lohse M, Usadel B. Trimmomatic: a flexible trimmer for Illumina sequence data. Bioinformatics. 2014;30(15):2114–20. 24695404 10.1093/bioinformatics/btu170 PMC4103590

[42] Luo R, Liu B, Xie Y, et al. SOAPdenovo2: an empirically improved memory-efficient short-read de novo assembler. GigaScience. 2012;1(1):18. 23587118 10.1186/2047-217X-1-18 PMC3626529

[43] Li H . Aligning sequence reads, clone sequences and assembly contigs with BWA-MEM. ArXiv preprint ArXiv. 2013.

[44] McKenna A, Hanna M, Banks E, et al. The genome analysis toolkit: a MapReduce framework for analyzing next-generation DNA Sequencing data. Genome Res. 2010;20(9):1297–303. 20644199 10.1101/gr.107524.110 PMC2928508

[45] Simâo FA, Waterhouse RM, Ioannidis P, et al. BUSCO: assessing genome assembly and annotation completeness with single-copy orthologs. Bioinformatics. 2015;31(19):3210–2. 26059717 10.1093/bioinformatics/btv351

[46] Grigoriev IV, Nikitin R, Haridas S, et al. MycoCosm portal: gearing up for 1000 fungal genomes. Nucleic Acids Res. 2014;42(D1):D699–704. 24297253 10.1093/nar/gkt1183 PMC3965089

[47] Peter J, De Chiara M, Friedrich A, et al. Genome evolution across 1,011 Saccharomyces cerevisiae isolates. Nature. 2018;556(7701):339–44. 29643504 10.1038/s41586-018-0030-5 PMC6784862

[48] Strope PK, Skelly DA, Kozmin SG, et al. The 100-genomes strains, an *S. cerevisiae* resource that illuminates its natural phenotypic and genotypic variation and emergence as an opportunistic pathogen. Genome Res. 2015;25(5):762–74. 25840857 10.1101/gr.185538.114 PMC4417123

[49] Wilkening S, Tekkedil MM, Lin G, et al. Genotyping 1000 yeast strains by next-generation sequencing. BMC Genomics. 2013;14(1):1–10. 23394869 10.1186/1471-2164-14-90 PMC3575377

[50] Zhu YO, Sherlock G, Petrov DA. Whole genome analysis of 132 clinical Saccharomyces cerevisiae strains reveals extensive ploidy variation. G3 (Bethesda). 2016;6(8):2421–34. 27317778 10.1534/g3.116.029397 PMC4978896

[51] Gerstein AC, Berman J. Shift and adapt: the costs and benefits of karyotype variations. Curr Opin Microbiol. 2015;26:130–6. 26321163 10.1016/j.mib.2015.06.010 PMC4577464

[52] Todd RT, Forche A, Selmecki A. Ploidy variation in fungi: polyploidy, aneuploidy, and genome evolution. Microbiol Spectrum, 2017;5(4):599–618. 10.1128/microbiolspec.funk-0051-2016 PMC5656283 28752816

[53] Corrochano LM, Kuo A, Marcet-Houben M, et al. Expansion of signal transduction pathways in fungi by extensive genome duplication. Curr Biol. 2016;26(12):1577–84. 27238284 10.1016/j.cub.2016.04.038 PMC5089372

[54] Ma L-J, Ibrahim AS, Skory C, et al. Genomic analysis of the basal lineage fungus *Rhizopus oryzae* reveals a whole-genome duplication. PLos Genet. 2009;5(7):1–11. 10.1371/journal.pgen.1000549 PMC2699053 19578406

[55] Mixão V, Gabaldón T. Yeast interspecies hybrids hybridization and emergence of virulence in opportunistic human yeast pathogens. Yeast. 2018;35(1):5–20. 28681409 10.1002/yea.3242 PMC5813172

[56] Schoenfelder KP, Fox DT. The expanding implications of polyploidy. J Cell Biol. 2015;209(4):485–91. 26008741 10.1083/jcb.201502016 PMC4442802

[57] Horn F, Üzüm Z, Möbius N, et al. Draft genome sequences of symbiotic and nonsymbiotic Rhizopus microsporus strains CBS 344.29 and ATCC 62417. Genome Announc. 2015;3(1):1–2. 10.1128/genomeA.01370-14 PMC4319578 25614557

[58] Chibucos MC, Soliman S, Gebremariam T, et al. An integrated genomic and transcriptomic survey of mucormycosis-causing fungi. Nat Commun. 2016;7:12218. 27447865 10.1038/ncomms12218 PMC4961843

[59] Burmester A, Karimi S, Wetzel J, et al. Complementation of a stable Met2-1 mutant of the zygomycete Absidia glauca by the corresponding wild-type allele of the mycoparasite Parasitella parasitica, transferred during infection. Microbiology. 2013;159(Pt 8):1639–48. 23704789 10.1099/mic.0.066910-0

[60] Brudno M, Chapman M, Göttgens B, et al. Gene prediction in eukaryotes with a generalized hidden Markov model that uses hints from external sources. BMC Bioinf. 2003;4(1):66. 10.1186/1471-2105-7-62 PMC1409804 16469098

[61] Altschul SF, Gish W, Miller W, et al. BLAST. J Mol Biol. 1990;215(3):403–10. 2231712 10.1016/S0022-2836(05)80360-2

[62] Consortium TU . Activities at the Universal Protein Resource (UniProt). Nucleic Acids Res. 2014;42(Database issue):D191–8. 24253303 10.1093/nar/gkt1140 PMC3965022

[63] Emms DM, Kelly S. OrthoFinder2: phylogenetic orthology inference for comparative genomics. Genome Biol. 2019;20(238):1–14. 31727128 10.1186/s13059-019-1832-y PMC6857279

[64] Gurevich A, Saveliev V, Vyahhi N, et al. QUAST: Quality Assessment Tool for Genome Assemblies. Bioinformatics. 2013;29(8):1072–5. 23422339 10.1093/bioinformatics/btt086 PMC3624806

[65] Berman J . Ploidy plasticity: a rapid and reversible strategy for adaptation to stress. FEMS Yeast Res. 2016;16(3):fow020. 26945893 10.1093/femsyr/fow020

[66] Scott AL, Richmond PA, Dowell RD, et al. The influence of polyploidy on the evolution of yeast grown in a sub-optimal carbon source. Mol Biol Evol. 2017;34(10):2690–703. 28957510 10.1093/molbev/msx205 PMC5850772

[67] Zörgö E, Chwialkowska K, Gjuvsland AB, et al. Ancient evolutionary trade-offs between yeast ploidy states. PLoS Genet. 2013;9(3):e1003388. 23555297 10.1371/journal.pgen.1003388 PMC3605057

[68] Ahrendt SR, Alisha Quandt C, Ciobanu D, et al. Leveraging single-cell genomics to expand the fungal tree of life. Nat Microbiol. 2018;3:1417–28. 30297742 10.1038/s41564-018-0261-0 PMC6784888

[69] Kravets A, Yang F, Bethlendy G, et al. Adaptation of *Candida albicans* to growth on sorbose via monosomy of chromosome 5 accompanied by duplication of another chromosome carrying a gene responsible for sorbose utilization. FEMS Yeast Res. 2014;14(5):708–13. 24702787 10.1111/1567-1364.12155 PMC4126865

[70] Lenassi M, Gostinčar C, Jackman S, et al. Whole genome duplication and enrichment of metal cation transporters revealed by de novo genome sequencing of extremely halotolerant black yeast *Hortaea werneckii*. PLoS One. 2013;8(8):1–18. 10.1371/journal.pone.0071328 PMC3744574 23977017

[71] Sinha S, Flibotte S, Niera M, et al. Insight into the recent genome duplication of the halophilic yeast *Hortaea werneckii*: combining an improved genome with gene expression and chromatin structure. G3 (Bethesda). 2017;7(7):2015–22. 28500048 10.1534/g3.117.040691 PMC5499112

[72] Anderson MZ, Saha A, Haseeb A, et al. A chromosome 4 trisomy contributes to increased fluconazole resistance in a clinical isolate of *Candida albicans*. Microbiology. 2017;163(6):856–65. 28640746 10.1099/mic.0.000478 PMC5737213

[73] Harrison BD, Hashemi J, Bibi M, et al. A tetraploid intermediate precedes aneuploid formation in yeasts exposed to fluconazole. PLoS Biol. 2014;12(3):1–18. 10.1371/journal.pbio.1001815 PMC3958355 24642609

[74] Gerstein AC, Fu MS, Mukaremera L, et al. Polyploid Titan cells produce haploid and aneuploid progeny to promote stress adaptation. MBio. 2015;6(5):1–14. 10.1128/mBio.01340-15 PMC4620463 26463162

[75] Li W, Averette AF, Desnos-Ollivier M, et al. Genetic diversity and genomic plasticity of *Cryptococcus neoformans* AD hybrid strains. G3 (Bethesda). 2012;2(1):83–97. 22384385 10.1534/g3.111.001255 PMC3276195

[76] Morrow Ca, Fraser Ja. Ploidy variation as an adaptive mechanism in human pathogenic fungi. Semin Cell Dev Biol. 2013;24(4):339–46. 23380396 10.1016/j.semcdb.2013.01.008

[77] Depotter JRl, Seidl MF, Wood TA, et al. Interspecific hybridization impacts host range and pathogenicity of filamentous microbes. Curr Opin Microbiol. 2016;32:7–13. 27116367 10.1016/j.mib.2016.04.005

[78] Garbelotto M, Gonthier P, Linzer R, et al. A shift in nuclear state as the result of natural interspecific hybridization between two North American taxa of the basidiomycete complex heterobasidion. Fungal Genet Biol. 2004;41(11):1046–51. 15465393 10.1016/j.fgb.2004.08.003

[79] Avramova M, Cibrario A, Peltier E, et al. *Brettanomyces bruxellensis* population survey reveals a diploid-triploid complex structured according to substrate of isolation and geographical distribution. Sci Rep. 2018;8(1):1–13. 29515178 10.1038/s41598-018-22580-7 PMC5841430

[80] Borneman AR, Zeppel R, Chambers PJ, et al. Insights into the Dekkera bruxellensis genomic landscape: comparative genomics reveals variations in ploidy and nutrient utilisation potential amongst wine isolates. PLoS Genet. 2014;10(2):e1004161. 24550744 10.1371/journal.pgen.1004161 PMC3923673

[81] James Sa, Bond CJ, Stratford M, et al. Molecular evidence for the existence of natural hybrids in the genus *Zygosaccharomyces*. FEMS Yeast Res. 2005;5(8):747–55. 15851103 10.1016/j.femsyr.2005.02.004

[82] Louis VL, Despons L, Friedrich A, et al. *Pichia sorbitophila*, an interspecies yeast hybrid, reveals early steps of genome resolution after polyploidization. G3 (Bethesda). 2012;2(2):299–311. 22384408 10.1534/g3.111.000745 PMC3284337

[83] Walther A, Hesselbart A, Wendland J. Genome sequence of Saccharomyces carlsbergensis, the world's first pure culture lager yeast. G3 (Bethesda). 2014;4(5):1–11. 24578374 10.1534/g3.113.010090 PMC4025477

[84] Naranjo-Ortíz MA, Molina M, Fuentes D, et al. Supporting data for “Karyon: a computational framework for the diagnosis of hybrids, aneuploids, and other non-standard architectures in genome assemblies.”. GigaScience Database. 2022. 10.5524/102242. PMC9540331 36205401

