# Supplementary material for: Karyon: a computational framework for the diagnosis of hybrids, aneuploids, and other nonstandard architectures in genome assemblies
Source: Gigascience. 2022 Oct 7;11:giac088. doi: 10.1093/gigascience/giac088 (PMC9540331; doi:10.1093/gigascience/giac088)
Supplement: giac088_GIGA-D-21-00155_Original_Submission [file giac088_giga-d-21-00155_original_submission.pdf]

GigaScience

Karyon: a computational framework for the diagnosis of hybrids, aneuploids, and other non-standard architectures in genome assemblies.

--Manuscript Draft--

**Manuscript Number:**	GIGA-D-21-00155
**Full Title:**	Karyon: a computational framework for the diagnosis of hybrids, aneuploids, and other non-standard architectures in genome assemblies.
**Article Type:**	Technical Note
**Funding Information:**	H2020 European Research Council (724173) — Dr Toni Gabaldon
**Abstract:**	Recent technological developments have made genome sequencing and assembly accessible to many groups. However, the presence in sequenced organisms of certain genomic features such as high heterozygosity, polyploidy, aneuploidy, or heterokaryosis can challenge current standard assembly procedures and result in highly fragmented assemblies. Hence, we hypothesized that genome databases must contain a non-negligible fraction of low-quality assemblies that result from such type of intrinsic genomic factors. Here we present Karyon, a Python-based toolkit that uses raw sequencing data and de novo genome assembly to assess several parameters and generate informative plots to assist in the identification of non-chanonical genomic traits. Karyon includes automated de novo genome assembly and variant calling pipelines. We tested Karyon by diagnosing 35 highly fragmented publicly available assemblies from 19 different Mucorales (Fungi) species. Our results show that 6 (17%) of the assemblies presented signs of unusual genomic configurations, suggesting that these are common, at least within the Fungi.
**Corresponding Author:**	Toni Gabaldon<br>IRB Barcelona: Institut de Recerca Biomedica<br>SPAIN
**Corresponding Author Secondary Information:**	
**Corresponding Author's Institution:**	IRB Barcelona: Institut de Recerca Biomedica
**Corresponding Author's Secondary Institution:**	
**First Author:**	Miguel A. Naranjo-Ortiz
**First Author Secondary Information:**	
**Order of Authors:**	Miguel A. Naranjo-Ortiz
	Manu Molina
	Verónica Mixão
	Toni Gabaldon
**Order of Authors Secondary Information:**	
**Additional Information:**	
**Question**	**Response**
Are you submitting this manuscript to a special series or article collection?	No
**Experimental design and statistics**<br><br>Full details of the experimental design and statistical methods used should be given	Yes

in the Methods section, as detailed in our Minimum Standards Reporting Checklist. Information essential to interpreting the data presented should be made available in the figure legends.<br><br>Have you included all the information requested in your manuscript?	
**Resources**<br><br>A description of all resources used, including antibodies, cell lines, animals and software tools, with enough information to allow them to be uniquely identified, should be included in the Methods section. Authors are strongly encouraged to cite Research Resource Identifiers (RRIDs) for antibodies, model organisms and tools, where possible.<br><br>Have you included the information requested as detailed in our Minimum Standards Reporting Checklist?	Yes
**Availability of data and materials**<br><br>All datasets and code on which the conclusions of the paper rely must be either included in your submission or deposited in publicly available repositories (where available and ethically appropriate), referencing such data using a unique identifier in the references and in the “Availability of Data and Materials” section of your manuscript.<br><br>Have you have met the above requirement as detailed in our Minimum Standards Reporting Checklist?	Yes

Manuscript Click here to access/download;Manuscript;Karyon_final.docx

Karyon: a computational framework for the diagnosis of hybrids, aneuploids, and other non-standard architectures in genome assemblies.

Miguel A. Naranjo-Ortiz[1,2,3], Manu Molina[1,2,4,5], Verónica Mixão[1,2,4,5], Toni Gabaldón[1,2,4,5,6*]

1) Centre for Genomic Regulation (CRG), The Barcelona Institute of Science and Technology, Dr. Aiguader 88, Barcelona 08003, Spain

2) Universitat Pompeu Fabra (UPF). 08003 Barcelona, Spain.

3) Clark University. 01610 Worcester, Massachusetts, United States of America.

4) Barcelona Supercomputing Centre (BSC-CNS). Jordi Girona, 29. 08034. Barcelona, Spain.

5) Institute for Research in Biomedicine (IRB Barcelona), The Barcelona Institute of Science and Technology, Baldiri Reixac, 10, 08028 Barcelona, Spain

6) ICREA, Pg. Lluís Companys 23, 08010 Barcelona, Spain.

*** author for correspondence: toni.gabaldon.bcn@gmail.com**

Abstract

Recent technological developments have made genome sequencing and assembly accessible to many groups. However, the presence in sequenced organisms of certain genomic features such as high heterozygosity, polyploidy, aneuploidy, or heterokaryosis can challenge current standard assembly procedures and result in highly fragmented assemblies. Hence, we hypothesized that genome databases must contain a non-negligible fraction of low-quality assemblies that result from such type of intrinsic genomic factors. Here we present Karyon, a Python-based toolkit that uses raw sequencing data and *de novo* genome assembly to assess several parameters and generate informative plots to assist in the identification of non-chanonical genomic traits. Karyon includes automated *de novo* genome assembly and variant calling pipelines. We tested Karyon by diagnosing 35 highly fragmented publicly available assemblies from 19 different Mucorales (Fungi) species. Our results show that 6 (17%) of the assemblies presented signs of unusual genomic configurations, suggesting that these are common, at least within the Fungi.

Keywords

Genome assembly, Heterozygosity, Hybridization, Polyploidy, Aneuploidy

Findings

- We present Karyon, a python-based bioinformatic pipeline that integrates genome assembly and a series of structural analyses for the diagnosis of problematic genomic

structures. Karyon is freely available in github and as a docker container (https://github.com/Gabaldonlab/karyon).

- We applied Karyon to 35 highly fragmented, publicly available genome assemblies to identify putative undescribed deviations in genomic architecture that might have caused problems in a standard assembly process. From 35 assemblies, six presented features that suggested possible underlying biological factors as the likely cause of the observed assembly fragmentation. Even though our sample size is small and restricted to a single lineage (Mucoromycotina), our results suggest that the number of unreported deviations in genome architecture in Fungi is considerable. This is emphasized if we consider that most researchers that have produced low quality assemblies are unlikely to publish their data.

Introduction

Recent developments in high-throughput sequencing and bioinformatic tools have made the process of sequencing the genome of a new organism a routine task for many laboratories. Genome assemblies provide an invaluable resource to understand the biology of an organism at different levels, from the molecular pathways that govern relevant phenotypes to the population structure of its species. The success of a genome assembly is limited by technical aspects as well as by intrinsic properties of the sequenced genome (Gabaldón and Alioto 2016) . A successful assembly depends on the quality, design, and depth of the sequencing libraries which must typically adapt to budget limitations. Naturally, if the sequencing methodology or the computational approaches are inappropriate, the results will be poor. However, additional

difficulties might arise independently of the methodology employed, due to intrinsic properties of the genome that interfere with genome assembly algorithms.

Biological factors affecting genome assemby quality

Whole-genome sequencing can be performed using short- or long-read sequencing technologies. The relatively higher throughput, lower error rate and cost still makes short read sequencing the most commonly used approach. Despite the increasing use of long-read technologies for assembly purposes, a large amount of genome assemblies available in public databases have been generated from short reads. Short-read assembly is usually performed using De Bruijn-based assemblers, which are mainly influenced by the number of different *k*-mers (all possible sequences of length *k*) present in the libraries. For this reason, the main intrinsic factors that compromise the success of a genome assembly are the genome size, the sequence heterozygosity, the abundance of low complexity regions (i.e., highly repetitive sequences), as well as the presence of high or uneven ploidy, contaminating sequences or extreme nucleotide compositions (Figure 1).

Genome size impacts computational costs, as many assembly algorithms scale non-linearly (Wajid and Serpedin 2012; Simpson and Pop 2015; Wajid et al. 2016) . Heterozygosity implies the existence of allelic differences within an individual, which can be single nucleotide polymorphisms, insertion-deletion differences, copy number variations or larger genomic rearrangements. Standard assembly algorithms have difficulty to differentiate between highly heterozygous regions and distinct but highly similar genomic regions (Hirsch and Robin Buell 2013; Leszek P Pryszcz and Gabaldón 2016) . This in turn results in fragmented assemblies

with inflated size compared to empirical measurements, as many of these regions appear duplicated (Leszek P Pryszcz and Gabaldón 2016) , often in short scaffolds. Similarly, repetitive or low complexity genomic regions are difficult to resolve without the aid of expensive experimental approaches, particularly when they span large genomic regions. Duplicated regions introduce multiple possible solutions to the process of scaffolding, increasing assembly fragmentation and computational costs (Hirsch and Robin Buell 2013; Wajid et al. 2016) .

Similarly, ploidy deviations can greatly affect genome assembly. The first possible ploidy deviation is polyploidy, which is the presence of more than two chromosomes for the majority of the genome. Polyploidy is generally associated to genome heterozygosity, as it increases the amount of possible states per site (Aguiar and Istrail 2013; Bonizzoni et al. 2016) . For a diploid site only two states are possible: heterozygous or homozygous, depending on whether the two alleles are different or equal, respectively. One can differentiate between the two possibilities by detecting frequencies of alternative states higher than expected from sequencing errors, which can be determined with statistical methods. For a triploid, however, there are two possible heterozygotic states, and differentiating between them depends on relative frequencies, which in turn might be affected by stochastic variations in coverage. For a diploid site, the null hypothesis is that the frequency of the alternative site is different from 0. A putative triploid site faces the same hypothesis, but it also needs to test whether it fits better a frequency of 0.5 than a frequency of 0.33 or 0.66 (Weiß et al. 2017) . Increasing sequencing depth reduces the effect of stochastic noise, but also increases experimental and computational costs, and the problem is even greater for higher ploidy levels.

Another deviation from the traditional eukaryotic karyotypic organization is aneuploidy, the presence of uneven numbers of chromosomes or large chromosomic regions (Torres, Williams, and Amon 2008; Gerstein and Berman 2015) that do not affect most of the genome. Aneuploidy tends to cause the same problems as polyploidy in assemblies, albeit with the effect being limited only to the aneuploid regions. Because of this, aneuploid regions will tend to appear highly fragmented and might remain undetected if the project is willing to accept a certain level of fragmentation. Genes present in these chromosomes will have a higher likelihood of being unannotated. Animal and plant genomics have traditionally considered aneuploidies as rare events, due to their deleterious effects on many of these organisms, specially during embryonic development. This paradigm is clearly false for many fungal (C. A. Anderson et al. 2015; Berman, Wertheimer, and Stone 2016; Mehrabi, Mirzadi Gohari, and Kema 2017) and protist (Mannaert et al. 2012; Tůmová et al. 2016) lineages, but the lack of traditional cytogenetic studies for many of these organisms makes difficult to have a clear global picture. Eukaryotic genomics has only recently started to focus on pangenomes (Golicz, Batley, and Edwards 2016; McCarthy and Fitzpatrick 2019; Sibbald et al. 2020; Naranjo- Ortiz and Gabaldón 2020; Gerdol et al. 2020) , but aneuploidies might be an important confounding factor for these studies.

In syncitial organisms, such as filamentous fungi or slime moulds, there is the possibility of coexistence of genetically different populations of nulei within a cytoplasm, a condition known as heterokaryosis (Maheshwari 2005; James et al. 2008; Strom and Bushley 2016) . Heterokaryosis is functionally similar to ploidy, although with some important differences. First, the relative proportions between heterozygous sites do not necessarily adjust to a simple fraction.

In other words, if two populations of nuclei coexist in a cytoplasm, the relative proportion of each type of nucleus is not necessarily equal. Second, since nuclei divide independently from each other, mitotic or meiotic recombination should be rare. This independency implies that any relative chromosomal rearrangements (i.e., duplications, deletions, translocations and inversions) between the two nuclear populations, either pre or post union, would remain in nuclear populations for long periods of time. These rearrangements introduce the aforementioned complications in genome assemblies, and some of these might be difficult to differentiate from other chromosomal aberrations. A similar phenomenon is chimerism, in which the body of an organism is composed by two or more populations of genetically distinct cells. However, in most cases chimeras arise from fusions of two or more embryos and as such the expected effect on heterozygosity is low. Certain lineages, specially colonial species, might arise by fusion of several genetically distinct individuals (Blanquer and Uriz 2011), but very little is known regarding the effect of chimerism in genome assemblies.

The presence of sequence contamination can greatly compromise the quality of the genome assembly (Schmieder and Edwards 2011; Kumar et al. 2013; Trivedi et al. 2014; Laetsch and Blaxter 2017; Lu and Salzberg 2018) . Extraneous sequences introduce noise, create chimeric contigs and might introduce errors in *k*-mer estimations. Highly diverse contaminations (e.g. From the gut microbiota) introduce sequences with highly variable level of coverage, heterozygosity and composition; while highly abundant contaminants (e.g. Symbiotic bacteria) are typically more homogeneous in all these parameters, but might still form chimeric contigs and would indirectly reduce the depth of coverage in the main genome. Contaminations reducing the signal of the main genome are particularly problematic for single cell sequencing projects

(Huang et al. 2015; Gawad, Koh, and Quake 2016). This is normally prevented by methodological means, but contaminating sequences are intrinsic for certain samples or even organisms, such as the case of symbiotic organisms (e.g. Lichens).

Finally, genomes with extreme compositions, typically very high or low GC content (GC%), can be difficult to assemble. For these genomes, the information contained by AT positions is different than the information contained by GC, as *k*-mers composed of the favoured nucleotide pair will appear at higher frequencies. GC% has a well-documented effect on some sequencing technologies, most notably on the quality of Illumina reads (Benjamini and Speed 2012; Ross et al. 2013) . Fortunately, GC% is easy to measure from raw reads, and some genome assemblers include options specially adapted for these cases (Bankevich et al. 2012; D. Scott and Ely 2014) . Low GC% is typically associated to high abundance of low complexity regions and transposable elements, but extreme GC% is also a hallmark of certain lineages, such as several groups of early diverging Fungi (e.g. Neocallimastigales, Mucoromycota, Zoopagomycota) (Naranjo-Ortiz and Gabaldón 2019) . Despite their effects in genome analyses, GC% in eukaryotic genomes is often ignored. For example, neither NCBI nor Mycocosm report GC% in their assembly information statistics, unlike the Genome OnLine Database (GOLD), which mostly deals with prokaryotic sequences.

If the presence of the factors outlined above is anticipated, specific technical approaches- both experimental and computational- can be used. Contaminating DNA can be identified easily because sequencing coverage, nucleotide composition and phylogenetic signal is usually different from the main genome and several programs have been developed to identify

contaminations (Schmieder and Edwards 2011; Kumar et al. 2013; Trivedi et al. 2014; Laetsch and Blaxter 2017; Lu and Salzberg 2018) . Ploidy can be easily estimated with cytogenetic techniques, which has been used for animals and plants since the XIXth century. Cytogenetic techniques are time consuming and difficult to interpret for some groups, such as the fungi, due to their smaller chromosomes. Computational approaches exist to estimate composition and ploidy from sequencing reads (Margarido et al. 2015; Mapleson, Accinelli, Kettleborough, Wright, Clavijo, et al. 2016; Weib et al. 2018) . Similarly, hybridization can often be detected based on phenotypic traits (intermediate phenotypes and hybrid vigor), an approach that requires phenotypes to observe to begin with. Genomes of hybrid organisms are heterozygous, and some genome assembly software have been designed to be able to handle this situation (Kajitani et al. 2014; Safonova, Bankevich, and Pevzner 2015; Leszek P Pryszcz and Gabaldón 2016) , but proper identification of hybrid lineages cannot be done without adequate population and phylogenetic analyses that require additional datasets that might not exist.

Thus, biological factors affecting genome assembly quality increase the overall costs of a project and require expertise that might not be available. Furthermore, such complications are often unforeseen and, if the possible causes for a low-quality assembly are not investigated, it could potentially lead to the direct deposition in databases of low-quality assemblies or even failure of the whole project. Given the difficulty of performing analyses on low quality assemblies, it is likely that published genomes are biased in favor of organisms with canonical genome architectures. As a result, it is so far unknown how common non-standard genome architectures really are.

Results

The Karyon toolkit

To aid in the identification of these non-canonical genomic architectures, we developed Karyon, a python-based toolkit that assesses several parameters of sequencing data and their derived assemblies that are common indicators of different intrinsic genomic features that may lead to poor assemblies using standard procedures. Karyon is comprised of different modules that can be used independently or sequentially. Karyon is written in Python 3 and freely available to download as a Docker build or as a standalone project in https://github.com/Gabaldonlab/karyon.

Karyon integrates Trimmomatic (Bolger, Lohse, and Usadel 2014) as an optional step to eliminate low quality positions and adapters from sequencing reads. It then uses that input to generate a *de novo* assembly using SPAdes v3.9.0 (Bankevich et al. 2012) , dipSPAdes v3.9.0 (Safonova, Bankevich, and Pevzner 2015) , Platanus v1.2.4 (Kajitani et al. 2014) or SOAPdenovo2 v2.04-r240 (Luo et al. 2012) . As dipSPAdes was specially designed to deal with highly polymorphic genomes (Safonova, Bankevich, and Pevzner 2015) , it was chosen to be Karyon's default option. Karyon then uses the *de novo* assembly to generate a reduced assembly using Redundans (Leszek P Pryszcz and Gabaldón 2016) . Redundans is a pipeline that collapses assembly fragments with high similarity in order to create an artificial haploid genome assembly. This assembly is then used as reference to map the original sequencing reads using BWA-MEM (Li 2013) , and generate a variant calling file with GATK v4.1.9.0 (McKenna et al. 2010) . A battery of analyses is then performed on the sequencing libraries, the assemblies, and the maps of coverage and genetic variation to generate plots that will aid in the diagnosis of the genomic structure. Figure 2 summarizes the pipeline.

Karyon uses the K-mer analysis toolkit (KAT) (Mapleson, et. al. 2016) to provide a *k*-mer (all possible sequences of length *k*) spectrum analysis as part of its report. From this analysis it produces frequency histograms representing coverage versus *k*-mer counts. These plots inform on ploidy and heterozygosity of a genome. In a haploid genome, for *k*-mers of enough size, most *k*-mers will appear either one or zero times, with unique *k*-mers having an average coverage roughly equal to the average depth of coverage. Deviations from these patterns suggest alternative architectures. For instance, the presence of two peaks in the *k*-mer plot may indicate a non-homozygous diploid. To complement these analyses and provide further information on the features of the genome, Karyon assesses scaffold length distributions, relationships between scaffold length and coverage, sliding-window analysis of coverage and genetic variation, as well as allele-frequency distributions per scaffold (Figure 2). In addition, Karyon uses nQuire (Weib et al. 2018) to estimate the likelihood of different ploidy levels in sliding windows per scaffold. Altogether, the interpretation of these analyses can be used to detect polyploidies, aneuploidies, hybridizations, heterokaryosis, large segmental duplications, or the presence of symbiont or contaminating sequences. Further details on each of these analyses and how they can be interpreted are provided in Karyon's manual . Illustrative, practical examples of their use can be seen in the next section.

Each of the steps is optional and can be controlled with flags in the main script. Additionally, the script uses a configuration file, that allows to define the options of each of the dependency programs. This configuration file is automatically created during the installation and can be modified with any text editor. We encourage the user to make a copy of the original

configuration file for future modification. Installation is fully automated, requiring no user input during the process.

Genomic survey in the Mucorales (Fungi)

To showcase the use of Karyon, we undertook an analysis of deposited fungal genomes in the order Mucorales. Fungi are in a particularly privileged position to assess the impact of non-canonical genomic architectures in genome assemblies. Fungi generally have small and compact genomes and can be often cultured under axenic conditions. As a result, the amount of sequenced fungal genomes is now in the order of thousands, including several strains for many species. Even more, comprehensive efforts to obtain a balanced coverage of the existing fungal diversity are ongoing, such as the 1000 fungal genomes (Grigoriev et al. 2014) and the 1000 yeast genomes initiatives (Wilkening et al. 2013; Strope et al. 2015; Zhu, Sherlock, and Petrov 2016; Peter et al. 2018) . Thus, fungi provide an excellent system to study the incidence of different genomic accidents in evolution (Gerstein and Berman 2015; Berman, Wertheimer, and Stone 2016; Todd, Forche, and Selmecki 2017) . Despite this, the quality of fungal genomes is often sub-optimal, and databases are riffed with highly fragmented assemblies. Genomic factors such as those discussed above might complicate genome assembly and be responsible for this observed fragmentation, at least partially. Considering this, we hypothesized that genome databases must contain a fraction of low-quality assemblies from fungal organisms that are caused by intrinsic genomic factors. If that is true, reanalysis of the raw data should lead us to describe novel genomic accidents and obtain a minimum estimate of their relative abundance.

We thus applied Karyon to a set of 35 publicly deposited genomes from the fungal order

Mucorales. Our results suggest that non-standard genomic organizations are not rare, and that future studies on other groups are likely to uncover many new cases. We selected the order Mucorales because this group comprises several described examples of whole-genome duplication, both at ancient and recent (Ma et al. 2009; Corrochano et al. 2016) . Many sequenced members of the clade come from clinical samples, an environment that is known to promote the emergence of different genomic accidents (Schoenfelder and Fox 2015; Todd, Forche, and Selmecki 2017; Mixão and Gabaldón 2018) . Additionally, several represented species included two or more sequenced isolates, allowing to get a glimpse at their intra-specific diversity. We obtained 35 genome assemblies from 19 different Mucorales species deposited in GenBank between January 1st 2005 and December 31th 2015 (Table 1). For 4 of the species, dipSPADes was unable to generate an assembly.

Karyon was run using the complete default pipeline. Most of the analyzed genomes (27, 79.4%) presented very low levels of heterozygosity and a relatively homogeneous coverage across the genome, suggesting that those strains are haploid or, if presenting higher ploidy, extremely homozygous. Fragmentation in these cases might be caused by insufficient coverage, presence of repetitive regions or some other methodological constraints. However, our pipeline uncovered cases that produced anomalous results in the different Karyon tests. Below we describe these cases and propose a plausible scenario to explain each of the obtained results based on the data obtained from the Karyon pipeline.

Rhizopus microsporus species complex

At the moment of this study, eight *Rhizopus microsporus* strains were deposited in the NCBI

database. Interestingly, three of them presented a genome size estimated around 25Mbp; four of them had a genome size close to 50Mbp; and one presented a genome size of 75Mbp. Only the three strains with a genome size of 25Mbp had sufficiently good assemblies considering they were based on short read, with a scaffold number below 1000, and thus were not selected for further analyses. Additionally, the raw libraries for one of the strains presenting 50Mbp genome assembly size (*Rhizopus microsporus* var. *chinensis* CCTCC M201021) were not publicly available and thus could not be part of the survey. For the remaining three strains with genome size close to 50Mbp (ATCC62417, CBS344.29 and var *rhizopodiformis* B7455), our *de novo* assembly pipeline recovered a genome size of approximately 40Mb, which is smaller than the assemblies deposited in NCBI (Table 1). The heterozygosity distribution in these assemblies shows that most of the genome presented a relatively uniform behavior with low heterozygosity. In all three cases, though, a considerable proportion of the genome appears with a highly variable coverage and increased heterozygosity (Figure 3). For these three strains, BlobTools (REF) shows an important fraction of the genome which seems to be of bacterial origin (Figure 3b) and thus we conclude that contamination is the main cause of assembly fragmentation.

The remaining strain, B9738, showed a surprisingly large genome size in both the assembly deposited in NCBI (75Mbp), and the one reconstructed here (71Mbp). The genome of *R. microsporus* B9738 presents an extremely low level of heterozygosity and a very homogeneous coverage. *K*-mer spectrum also shows just one very clear peak. All in all, all this suggests that B9738 is haploid, despite presenting a 3-fold increase in genome size as compared to other strains of the same species (Figure 4). Augustus gene prediction returned a total of 21,300 gene models, which is an unusually large number for a filamentous fungus. As a reference, the seven

genomes in the Rhizopodaceae, to which *Rhizopus* belongs, available in Mycocosm range from 25 to 46 Mbp and from 10,781 to 17,676 annotated genes. Contamination analysis does not suggest the presence of widespread contamination that could explain such over-inflated genome (Figure 4). For this reason, we suggest that B9738 might be a misidentified strain that does not belong to the *R. microsporus* species complex. Indeed, phylogenomic analyses recover B9738 as sister to a clade containing *Mucor* and *Parasitella*, rather than allied with the rest of the *Rhizopus microsporus* species clade (Figure 5), thus supporting a misidentification. It is noteworthy that no sequenced species of either *Mucor* or *Parasitella* have genomes above 49Mbp or with more than 15,000 genes, at least from the available genomes in Mycocosm.

***Mucor racemosis* B9645**

Analyses on *Mucor racemosus* B9645 depicted a genome with a dual behavior. The distribution of heterozygosity and coverage showed two peaks with very low heterozygosity but with different coverage (Figure 6b). This was further confirmed by the *k*-mer spectrum analysis, which revealed two clear peaks (Figure 6a). The genome available in NCBI is 65.5Mbp-long, noticeably larger than the 45.9Mbp we recovered in our analyses (Table 1). The reduction step of Redundans cannot explain this difference, as the assembly size prior to this step is already 46.8Mbp, very close to the final result. Our analyses suggest that contaminating sequences are very minor and do not explain the observed pattern (Figure 6). We hypothesize that *M. racemosus* B9645 is a hemidiploid, which presents a portion of its genome in haploid state, and other portion in a highly homozygous diploid state. Due to the low heterozygosity exhibited by this strain, the observed genome architecture might have arisen by either autopolyploidization followed by chromosome loss or by chromosomal duplications.

Lichtheimia ramosa B5399

The Karyon assembly for this genome was only 26.6Mbp, much smaller than the NCBI assembly (45.6Mpb long, Table 1). Unlike other genomes, our assembly presented a considerable improved quality, going from 3,968 scaffolds and N50 of 33,650 in the NCBI assembly to 861 scaffolds and N50 of 133,635 in our own assembly. *L. ramosa* presents a heterozygosity level around 3% in its diploid peak (Figure 7). All considered, we propose that *L. ramosa* B5399 is a diploid with high heterozygosity and several aneuploid (both aneuploid and triploid) , likely resulting from mating between two distantly related strains, and the NCBI assembly is inflated as a consequence of this situation.

Methods

Sequencing data

We downloaded raw data from libraries deposited at Short Read Archive (SRA)(National Centre for Biotechnology Information 2015) of those species in the Mucorales with a highly fragmented assembly (>1,000 scaffolds), which included at least one paired-end Illumina library larger than 1Gb after quality filtering (Table 1), to ensure at least a decent coverage. Since most of our genomes have typical assembly sizes around 40Mbp, this measure ensures a bare minimum average coverage of 20.

De novo gene annotation

We used Augustus v3.1.0. (Brudno et al. 2003) to obtain a *de novo* gene prediction using the included *Rhizopus oryzae* trained model.

Contamination detection

For each of the conflictive assemblies, we generated an Augustus prediction. Then, we used Blastp (Stephen F. Altschul, Warren Gish, Webb Miller 1990) to query the whole proteome against Uniref100 (Consortium 2014) . Since the genomes come from public databases, their own proteins should appear as hits and thus we retrieved the 10 best hits. We have used these hits to assign a taxonomic profile. Additionally, we have used the predicted Augustus CDS to map sequencing reads with GATK. With both the taxonomic profile and the variant calling file, we have run BlobTools (Laetsch and Blaxter 2017) in order to identify the presence of widespread contamination in the sequencing libraries.

Phylogenomic analyses

In order to identify the phylogenetic position of *R. microsporus* B9738 we used the Augustus gene prediction and the proteome of 24 other zygomycetes to run OrthoFinder v.2.3.3 (D.M. and S. 2018) with the flags -S blast and -m msa.

Discussion

As genome sequencing has moved away from model organisms, it has become apparent that many possible genomic architectures are possible, and many do exist in a wide range of organisms. Most of these genomic accidents are difficult to identify from sequencing data alone. As far as we know, Karyon is the first software developed with the intention of analyzing the presence of such genomic incidents during the process of *de novo* genome assembly. We have designed this software to be easy to install and use, with the possibility of installation from both GitHub and Docker.

Despite the success in the implemented strategy, we consider our software has several limitations. Karyon requires an assembly step and variant calling protocol, for which some default options are included. However, the included programs might not suit every need. For example, extremely large genomes might require alternative assemblers that are not included in our pipeline, or some users might prefer a different set of programs for the variant calling protocol. For those cases Karyon can be used as independent steps (Figure 2). At this moment, the pipeline assumes the use of at least one Illumina paired-end sequencing library. Because of this, we recommend the use of other genome assemblers if other sequencing technologies (i.e., Nanopore or PacBio long reads) are to be used, and the same goes for variant calling protocols. Fortunately, thanks to the modular nature of Karyon, implementation of new programs is straightforward.

We provided a practical example of the usage of Karyon on a publicly available set of fungal genomes from the order Mucorales. While the majority of analyzed assemblies show no sign of any of the considered biological conditions, we were able to effectively find underlying non-standard genomic architectures that had been previously unnoticed in these assemblies. These results suggest that many authors do not take into consideration this kind of genomic accidents, which in turn greatly hampers the results that might be obtained from them.

How common are these non-standard genomic architectures? Our results suggest that they might be quite abundant, although, so far, they are restricted to a limited selection of species within a narrow clade of Fungi. As such, these genomic anomalies might, or might not, be common in other lineages. However, we consider that there are three important arguments in favor for

considering our dataset an underestimation of the abundance of unorthodox fungal genomes, even within the taxonomic range we have selected. The first one is the fact that fungal biomass used for DNA extraction and subsequent sequencing typically comes from cultures. This implies an important ecological step in which the fungus grows at optimal speed and in the absence of most stressors. Aneuploidies, polyploidies and other similar genomic rearrangements are common in the presence of stressors (C. A. Anderson et al. 2015; Berman 2016; Berman, Wertheimer, and Stone 2016; Todd, Forche, and Selmecki 2017) , but seem to be out-competed by euploid cells under optimal conditions (Kumaran, Yang, and Leu 2013; Zörgö et al. 2013; A. L. Scott et al. 2017) . Hence, isolates growing in rich medium will be selected to lose most chromosomal aberrations they might present. Analogously, many of these chromosomal aberrations might exist in nature but are unable to grow on optimal medium. The advance of environmental sequencing and single cell based technologies might cast some light in this matter in coming years. Supporting this argument, Ahrendt et al. sequenced several environmental isolates of zoosporic and zygomycetous microfungi using these techniques and found several aneuploids and polyploids (Ahrendt et al. 2018) . The frequency of unconventional genomic architectures is very likely lineage-dependent. While some of these are well known, such as the dikaryotic phase in Agaricomycetes or the macro and micronuclei of ciliates, strange genomic architectures might be common in more obscure lineages. This not only represents a yet-to-know facet of the biology of these organisms, but it could potentially complicate their study. The third factor to consider is purely human. The datasets we have analyzed were uploaded by researchers who considered they were good enough to be uploaded to a public repository. Thus, it is to be expected that many more low-quality assemblies would have never been never deposited and sit forgotten in the disks of laboratory computers, if not discarded completely.

Even if we consider these possible biases as negligible, our results recover a significant fraction of publicly available genomes with unorthodox genomic configurations. These have been correlated in many fungal groups with adaptation to novel environments (Lenassi et al. 2013; Kravets et al. 2014; Sinha et al. 2017) , resistance to antifungals (Harrison et al. 2014; M. Z. Anderson et al. 2017) , pathogenic capabilities toward both animals (W. Li et al. 2012; Morrow and Fraser 2013; Leszek Piotr Pryszcz 2014; Gerstein et al. 2015; Mixão and Gabaldón 2018) and plants (Garbelotto et al. 2004; Depotter et al. 2016) and adaptation to industrial settings (S. a. James et al. 2005; Louis et al. 2012; Borneman et al. 2014; Walther, Hesselbart, and Wendland 2014; Peter et al. 2018; Avramova et al. 2018) . Beyond that, contamination in sequencing libraries is a problem that can affect any assembly project and might mislead downstream inferences if left unaddressed. Validation of published results goes far beyond the interest of discovering overlooked findings. Comparative genomic studies are limited in their scope and reliability by the quality of assembly and annotation of the genomes, factors that can be greatly compromised by these biological factors. Comparative studies commonly require the use of flagship genomes that represent a given taxon. Often, this generates a chronology of comparisons versus the reference that shapes the perspective on the group. As such, artifacts and errors in strategic genome assemblies, such as reference strains or strains in groups with few represented species, might have a domino effect impacting future studies. Long-read sequencing technologies, which are increasingly being used for genome assembly projects, hold the promise of providing much more information that could be used to resolve many of these unorthodox genomic architectures. However, these approaches require novel computational approaches to fully employ their potential.

Conflict Statement

The authors state that they have no conflicts of interests.

Acknowledgements

TG group acknowledges support from the Spanish Ministry of Science and Innovation for grant PGC2018-099921-B-I00, cofounded by European Regional Development Fund (ERDF); from the Catalan Research Agency (AGAUR) SGR423; from the European Union's Horizon 2020 research and innovation programme (ERC-2016-724173); from the Gordon and Betty Moore Foundation (Grant GBMF9742) and from the Instituto de Salud Carlos III (INB Grant PT17/0009/0023 - ISCIII-SGEFI/ERDF).

Bibliography

Aguiar, Derek, and Sorin Istrail. 2013. "Haplotype Assembly in Polyploid Genomes and Identical by Descent Shared Tracts." *Bioinformatics (Oxford, England)* 29 (13): i352-60. https://doi.org/10.1093/bioinformatics/btt213.

Ahrendt, Steven R., C. Alisha Quandt, Doina Ciobanu, Alicia Clum, Asaf Salamov, Bill Andreopoulos, Jan-Fang Cheng, et al. 2018. "Leveraging Single-Cell Genomics to Expand the Fungal Tree of Life." *Nature Microbiology* 3 (October): 1417–1428. https://doi.org/10.1038/s41564-018-0261-0.

Anderson, Cori A, Samantha Roberts, Huaiying Zhang, Courtney M Kelly, Alexxy Kendall, ChangHwan Lee, John Gerstenberger, Aaron B Koenig, Ruth Kabeche, and Amy S

Gladfelter. 2015. “Ploidy Variation in Multinucleate Cells Changes under Stress.” *Molecular Biology of the Cell* 26 (6): 1129–40. https://doi.org/10.1091/mbc.E14-09-1375.

Anderson, Matthew Z, Amrita Saha, Abid Haseeb, and Richard J Bennett. 2017. “A Chromosome 4 Trisomy Contributes to Increased Fluconazole Resistance in a Clinical Isolate of *Candida albicans*.” *Microbiology (Reading, England)* 163 (6): 856–65. https://doi.org/10.1099/mic.0.000478.

Avramova, Marta, Alice Cibrario, Emilien Peltier, Monika Coton, Emmanuel Coton, Joseph Schacherer, Giuseppe Spano, et al. 2018. “*Brettanomyces bruxellensis* Population Survey Reveals a Diploid-Triploid Complex Structured According to Substrate of Isolation and Geographical Distribution.” *Scientific Reports* 8 (1): 1–13. https://doi.org/10.1038/s41598-018-22580-7.

Bankevich, Anton, Sergey Nurk, Dmitry Antipov, Alexey A. Gurevich, Mikhail Dvorkin, Alexander S. Kulikov, Valery M. Lesin, et al. 2012. “SPAdes: A New Genome Assembly Algorithm and Its Applications to Single-Cell Sequencing.” *Journal of Computational Biology* 19 (5): 455–77. https://doi.org/10.1089/cmb.2012.0021.

Benjamini, Yuval, and Terence P. Speed. 2012. “Summarizing and Correcting the GC Content Bias in High-Throughput Sequencing.” *Nucleic Acids Research*. https://doi.org/10.1093/nar/gks001.

Berman, Judith. 2016. “Ploidy Plasticity: A Rapid and Reversible Strategy for Adaptation to Stress.” Edited by Carol Munro. *FEMS Yeast Research* 16 (3): fow020. https://doi.org/10.1093/femsyr/fow020.

Berman, Judith, Noa Blutraich Wertheimer, and Neil Stone. 2016. “Ploidy Dynamics and

Evolvability in Fungi.” *Philosophical Transactions of the Royal Society of London B: Biological Sciences* 371 (20150461): 1–11. https://doi.org/10.1098/rstb.2015.0461.

Blanquer, Andrea, and Maria-J. Uriz. 2011. “‘Living Together Apart’: The Hidden Genetic Diversity of Sponge Populations.” *Molecular Biology and Evolution* 28 (9): 2435–38. https://doi.org/10.1093/molbev/msr096.

Bolger, Anthony M, Marc Lohse, and Bjoern Usadel. 2014. “Trimmomatic: A Flexible Trimmer for Illumina Sequence Data.” *Bioinformatics (Oxford, England)* 30 (15): 2114–20. https://doi.org/10.1093/bioinformatics/btu170.

Bonizzoni, Paola, Riccardo Dondi, Gunnar W. Klau, Yuri Pirola, Nadia Pisanti, and Simone Zaccaria. 2016. “On the Minimum Error Correction Problem for Haplotype Assembly in Diploid and Polyploid Genomes.” *Journal of Computational Biology* 23 (9): 718–36. https://doi.org/10.1089/cmb.2015.0220.

Borneman, Anthony R, Ryan Zeppel, Paul J Chambers, and Chris D Curtin. 2014. “Insights into the Dekkera Bruxellensis Genomic Landscape: Comparative Genomics Reveals Variations in Ploidy and Nutrient Utilisation Potential amongst Wine Isolates.” *PLoS Genetics* 10 (2): e1004161. https://doi.org/10.1371/journal.pgen.1004161.

Brudno, Michael, Michael Chapman, Berthold Göttgens, Serafim Batzoglou, Burkhard Morgenstern, S Knowles, JM Bye, DM Beare, and I Dunham. 2003. “Gene Prediction in Eukaryotes with a Generalized Hidden Markov Model That Uses Hints from External Sources.” *BMC Bioinformatics* 4 (1): 66. https://doi.org/10.1186/1471-2105-4-66.

Consortium, The Uniprot. 2014. “Activities at the Universal Protein Resource (UniProt).” *Nucleic Acids Research* 42 (Database issue): D191-8. https://doi.org/10.1093/nar/gkt1140.

Corrochano, Luis M, Alan Kuo, Marina Marcet-Houben, Silvia Polaino, Asaf Salamov, José M Villalobos-Escobedo, Jane Grimwood, et al. 2016. "Expansion of Signal Transduction Pathways in Fungi by Extensive Genome Duplication." *Current Biology* 26 (12): 1577–84. https://doi.org/10.1016/j.cub.2016.04.038.

D.M., Emms, and Kelly S. 2018. "OrthoFinder2: Fast and Accurate Phylogenomic Orthology Analysis from Gene Sequences." *BioRxiv*, November, 466201. https://doi.org/10.1101/466201.

Depotter, Jasper Rl, Michael F Seidl, Thomas A Wood, and Bart Phj Thomma. 2016. "Interspecific Hybridization Impacts Host Range and Pathogenicity of Filamentous Microbes." *Current Opinion in Microbiology* 32: 7–13. https://doi.org/10.1016/j.mib.2016.04.005.

Gabaldón, Toni, and Tyler S Alioto. 2016. "Whole-Genome Sequencing Recommendations." In *Field Guidelines for Genetic Experimental Designs in High-Throughput Sequencing*, 13–42. https://doi.org/10.1007/978-3-319-31350-4.

Garbelotto, Matteo, Paolo Gonthier, Rachel Linzer, Giovanni Nicolotti, and William Otrosina. 2004. "A Shift in Nuclear State as the Result of Natural Interspecific Hybridization between Two North American Taxa of the Basidiomycete Complex Heterobasidion." *Fungal Genetics and Biology : FG & B* 41 (11): 1046–51. https://doi.org/10.1016/j.fgb.2004.08.003.

Gawad, Charles, Winston Koh, and Stephen R. Quake. 2016. "Single-Cell Genome Sequencing: Current State of the Science." *Nature Reviews Genetics*. Nature Publishing Group. https://doi.org/10.1038/nrg.2015.16.

Gerdol, Marco, Rebeca Moreira, Fernando Cruz, Jessica Gómez-Garrido, Anna Vlasova, Umberto Rosani, Paola Venier, et al. 2020. “Massive Gene Presence-Absence Variation Shapes an Open Pan-Genome in the Mediterranean Mussel.” *Genome Biology* 21 (1): 275. https://doi.org/10.1186/s13059-020-02180-3.

Gerstein, Aleeza C, and Judith Berman. 2015. “Shift and Adapt: The Costs and Benefits of Karyotype Variations.” *Current Opinion in Microbiology* 26 (August): 130–36. https://doi.org/10.1016/j.mib.2015.06.010.

Gerstein, Aleeza C, Man Shun Fu, Liliane Mukaremera, Zhongming Li, Kate L Ormerod, James A Fraser, Judith Berman, and Kirsten Nielsen. 2015. “Polyploid Titan Cells Produce Haploid and Aneuploid Progeny to Promote Stress Adaptation.” *MBio* 6 (5): 1–14. https://doi.org/10.1128/mBio.01340-15.

Golicz, Agnieszka A., Jacqueline Batley, and David Edwards. 2016. “Towards Plant Pangenomics.” *Plant Biotechnology Journal* 14 (4): 1099–1105. https://doi.org/10.1111/pbi.12499.

Grigoriev, Igor V, Roman Nikitin, Sajeet Haridas, Alan Kuo, Robin Ohm, Robert Otillar, Robert Riley, et al. 2014. “MycoCosm Portal: Gearing up for 1000 Fungal Genomes.” *Nucleic Acids Research* 42: 699–704. https://doi.org/10.1093/nar/gkt1183.

Harrison, Benjamin D, Jordan Hashemi, Maayan Bibi, Rebecca Pulver, Danny Bavli, Yaakov Nahmias, Melanie Wellington, Guillermo Sapiro, and Judith Berman. 2014. “A Tetraploid Intermediate Precedes Aneuploid Formation in Yeasts Exposed to Fluconazole.” *PLoS Biology* 12 (3): 1–18. https://doi.org/10.1371/journal.pbio.1001815.

Hirsch, Candice N., and C. Robin Buell. 2013. “Tapping the Promise of Genomics in Species

with Complex, Nonmodel Genomes.” *Annual Review of Plant Biology* 64 (1): 89–110. https://doi.org/10.1146/annurev-arplant-050312-120237.

Huang, Lei, Fei Ma, Alec Chapman, Sijia Lu, and Xiaoliang Sunney Xie. 2015. “Single-Cell Whole-Genome Amplification and Sequencing: Methodology and Applications.” *Annual Review of Genomics and Human Genetics* 16 (August): 79–102. https://doi.org/10.1146/annurev-genom-090413-025352.

James, Stephen a., Christopher J. Bond, Malcolm Stratford, and Ian N. Roberts. 2005. “Molecular Evidence for the Existence of Natural Hybrids in the Genus *Zygosaccharomyces*.” *FEMS Yeast Research* 5 (8): 747–55. https://doi.org/10.1016/j.femsyr.2005.02.004.

James, Timothy Y., Jan Stenlid, Åke Olson, and Hanna Johannesson. 2008. “Evolutionary Significance of Imbalanced Nuclear Ratios within Heterokaryons of the Basidiomycete Fungus *Heterobasidion parviporum.*” *Evolution* 62 (9): 2279–96. https://doi.org/10.1111/j.1558-5646.2008.00462.x.

Kajitani, Rei, Kouta Toshimoto, Hideki Noguchi, Atsushi Toyoda, Yoshitoshi Ogura, Miki Okuno, Mitsuru Yabana, et al. 2014. “Efficient de Novo Assembly of Highly Heterozygous Genomes from Whole-Genome Shotgun Short Reads.” *Genome Research* 24 (8): 1384–95. https://doi.org/10.1101/gr.170720.113.

Kravets, Anatoliy, Feng Yang, Gabor Bethlendy, Fred Sherman, and Elena Rustchenko. 2014. “Adaptation of *Candida albicans* to Growth on Sorbose via Monosomy of Chromosome 5 Accompanied by Duplication of Another Chromosome Carrying a Gene Responsible for Sorbose Utilization.” *FEMS Yeast Research* 14 (5): 708–13. https://doi.org/10.1111/1567-

1364.12155.Adaptation.

Kumar, Sujai, Martin Jones, Georgios Koutsovoulos, Michael Clarke, and Mark Blaxter. 2013. “Blobology: Exploring Raw Genome Data for Contaminants, Symbionts and Parasites Using Taxon-Annotated GC-Coverage Plots.” *Frontiers in Genetics* 4 (November): 237. https://doi.org/10.3389/fgene.2013.00237.

Kumaran, Rajaraman, Shi Yow Yang, and Jun Yi Leu. 2013. “Characterization of Chromosome Stability in Diploid, Polyploid and Hybrid Yeast Cells.” *PLoS ONE* 8 (7). https://doi.org/10.1371/journal.pone.0068094.

Laetsch, Dominik R., and Mark L. Blaxter. 2017. “BlobTools: Interrogation of Genome Assemblies.” *F1000Research* 6: 1287. https://doi.org/10.12688/f1000research.12232.1.

Lenassi, Metka, Cene Gostinčar, Shaun Jackman, Martina Turk, Ivan Sadowski, Corey Nislow, Steven Jones, Inanc Birol, Nina Gunde Cimerman, and Ana Plemenitaš. 2013. “Whole Genome Duplication and Enrichment of Metal Cation Transporters Revealed by De Novo Genome Sequencing of Extremely Halotolerant Black Yeast *Hortaea werneckii*.” Edited by Jason E. Stajich. *PLoS ONE* 8 (8): 1–18. https://doi.org/10.1371/journal.pone.0071328.

Li, Heng. 2013. “Aligning Sequence Reads, Clone Sequences and Assembly Contigs with BWA-MEM.” *ArXiv Preprint ArXiv*, 1–3.

Li, Wenjun, Anna Floyd Averette, Marie Desnos-Ollivier, Min Ni, Françoise Dromer, and Joseph Heitman. 2012. “Genetic Diversity and Genomic Plasticity of *Cryptococcus neoformans* AD Hybrid Strains.” *G3: Genes, Genomes, Genetics* 2 (1): 83–97. https://doi.org/10.1534/g3.111.001255.

Louis, V. L., L. Despons, A. Friedrich, T. Martin, P. Durrens, S. Casaregola, C. Neuveglise, et al. 2012. “*Pichia sorbitophila*, an Interspecies Yeast Hybrid, Reveals Early Steps of Genome Resolution After Polyploidization.” *G3: Genes, Genomes, Genetics* 2 (2): 299–311. https://doi.org/10.1534/g3.111.000745.

Lu, Jennifer, and Steven L. Salzberg. 2018. “Removing Contaminants from Databases of Draft Genomes.” Edited by Fengzhu Sun. *PLOS Computational Biology* 14 (6): e1006277. https://doi.org/10.1371/journal.pcbi.1006277.

Luo, Ruibang, Binghang Liu, Yinlong Xie, Zhenyu Li, Weihua Huang, Jianying Yuan, Guangzhu He, et al. 2012. “SOAPdenovo2: An Empirically Improved Memory-Efficient Short-Read de Novo Assembler.” *GigaScience* 1 (1): 18. https://doi.org/10.1186/2047-217X-1-18.

Ma, Li-Jun, Ashraf S. Ibrahim, Christopher Skory, Manfred G. Grabherr, Gertraud Burger, Margi Butler, Marek Elias, et al. 2009. “Genomic Analysis of the Basal Lineage Fungus *Rhizopus oryzae* Reveals a Whole-Genome Duplication.” Edited by Hiten D. Madhani. *PLoS Genetics* 5 (7): 1–11. https://doi.org/10.1371/journal.pgen.1000549.

Maheshwari, Ramesh. 2005. “Nuclear Behavior in Fungal Hyphae.” *FEMS Microbiology Letters* 249: 7–14. https://doi.org/10.1016/j.femsle.2005.06.031.

Mannaert, An, Tim Downing, Hideo Imamura, and Jean Claude Dujardin. 2012. “Adaptive Mechanisms in Pathogens: Universal Aneuploidy in Leishmania.” *Trends in Parasitology*. https://doi.org/10.1016/j.pt.2012.06.003.

Mapleson, Daniel, Gonzalo Garcia Accinelli, George Kettleborough, Jonathan Wright, and Bernardo J Clavijo. 2016. “KAT: A K-Mer Analysis Toolkit to Quality Control NGS

Datasets and Genome Assemblies." *Bioinformatics* 33 (4): 574–76. https://doi.org/10.1093/bioinformatics/btw663.

Margarido, Gabriel R. A., David Heckerman, EW Myers, GG Sutton, AL Delcher, IM Dew, DP Fasulo, et al. 2015. "ConPADE: Genome Assembly Ploidy Estimation from Next-Generation Sequencing Data.". *PLOS Computational Biology* 11 (4): e1004229. https://doi.org/10.1371/journal.pcbi.1004229.

McCarthy, Charley G. P., and David A. Fitzpatrick. 2019. "Pan-Genome Analyses of Model Fungal Species." *Microbial Genomics* 5 (2): 1–23. https://doi.org/10.1099/mgen.0.000243.

McKenna, Aaron, Matthew Hanna, Eric Banks, Andrey Sivachenko, Kristian Cibulskis, Andrew Kernytsky, Kiran Garimella, et al. 2010. "The Genome Analysis Toolkit: A MapReduce Framework for Analyzing next-Generation DNA Sequencing Data." *Genome Research* 20 (9): 1297–1303. https://doi.org/10.1101/gr.107524.110.

Mehrabi, Rahim, Amir Mirzadi Gohari, and Gert H.J. Kema. 2017. "Karyotype Variability in Plant-Pathogenic Fungi." *Annual Review of Phytopathology* 55 (1): 483–503. https://doi.org/10.1146/annurev-phyto-080615-095928.

Mixão, Verónica, and Toni Gabaldón. 2018. "Yeast Interspecies Hybrids Hybridization and Emergence of Virulence in Opportunistic Human Yeast Pathogens." *Yeast* 35: 5–20. https://doi.org/10.1002/yea.3242.

Morrow, Carl a., and James a. Fraser. 2013. "Ploidy Variation as an Adaptive Mechanism in Human Pathogenic Fungi." *Seminars in Cell and Developmental Biology* 24 (4): 339–46. https://doi.org/10.1016/j.semcdb.2013.01.008.

Naranjo-Ortiz, M.A., and T. Gabaldón. 2019. "Fungal Evolution: Diversity, Taxonomy and

Phylogeny of the Fungi.” *Biological Reviews* 94 (6). https://doi.org/10.1111/brv.12550.

Naranjo‐ Ortiz, Miguel A., and Toni Gabaldón. 2020. “Fungal Evolution: Cellular, Genomic and Metabolic Complexity.” *Biological Reviews*, April, brv.12605. https://doi.org/10.1111/brv.12605.

National Centre for Biotechnology Information. 2015. “SRA: Sequence Read Archive.” *NCBI Handout Series*, 4. https://www.ncbi.nlm.nih.gov/core/assets/sra/files/Factsheet_SRA.pdf.

Peter, Jackson, Matteo De Chiara, Anne Friedrich, Jia-Xing Yue, David Pflieger, Anders Bergström, Anastasie Sigwalt, et al. 2018. “Genome Evolution across 1,011 Saccharomyces Cerevisiae Isolates.” *Nature* 556 (7701): 339–44. https://doi.org/10.1038/s41586-018-0030-5.

Pryszcz, Leszek P, and Toni Gabaldón. 2016. “Redundans : An Assembly Pipeline for Highly Heterozygous Genomes.” *Nucleic Acids Research* 8 (44): 1–16. https://doi.org/10.1093/nar/gkw294.

Pryszcz, Leszek Piotr. 2014. “Comparative Genomics to Unravel Virulence Mechanisms in Fungal Human Pathogens.”

Ross, Michael G., Carsten Russ, Maura Costello, Andrew Hollinger, Niall J. Lennon, Ryan Hegarty, Chad Nusbaum, and David B. Jaffe. 2013. “Characterizing and Measuring Bias in Sequence Data.” *Genome Biology*. https://doi.org/10.1186/gb-2013-14-5-r51.

Safonova, Yana, Anton Bankevich, and Pavel A Pevzner. 2015. “DipSPAdes: Assembler for Highly Polymorphic Diploid Genomes.” *Journal of Computational Biology : A Journal of Computational Molecular Cell Biology* 22 (6): 528–45. https://doi.org/10.1089/cmb.2014.0153.

Schmieder, Robert, and Robert Edwards. 2011. “Fast Identification and Removal of Sequence Contamination from Genomic and Metagenomic Datasets.” Edited by Francisco Rodriguez-Valera. *PLoS ONE* 6 (3): e17288. https://doi.org/10.1371/journal.pone.0017288.

Schoenfelder, Kevin P, and Donald T Fox. 2015. “The Expanding Implications of Polyploidy.” *The Journal of Cell Biology* 209 (4): 485–91. https://doi.org/10.1083/jcb.201502016.

Scott, Amber L, Phillip A Richmond, Robin D Dowell, and Anna M Selmecki. 2017. “The Influence of Polyploidy on the Evolution of Yeast Grown in a Sub-Optimal Carbon Source.” *Molecular Biology and Evolution* 34 (10): 2690–2703. https://doi.org/10.1093/molbev/msx205.

Scott, Derrick, and Bert Ely. 2014. “Comparison of Genome Sequencing Technology and Assembly Methods for the Analysis of a GC-Rich Bacterial Genome.” *Current Microbiology* 70 (3): 338–44. https://doi.org/10.1007/s00284-014-0721-6.

Sibbald, Shannon J., Laura Eme, John M. Archibald, and Andrew J. Roger. 2020. “Lateral Gene Transfer Mechanisms and Pan-Genomes in Eukaryotes.” *Trends in Parasitology,* August. https://doi.org/10.1016/j.pt.2020.07.014.

Simpson, Jared T., and Mihai Pop. 2015. “The Theory and Practice of Genome Sequence Assembly.” *Annual Review of Genomics and Human Genetics* 16 (1): 153–72. https://doi.org/10.1146/annurev-genom-090314-050032.

Sinha, Sunita, Stephane Flibotte, Mauricio Niera, Sean Formby, Ana Plemenitaš, Nina Gunde Cimerman, Metka Lenassi, Cene Gostinčar, Jason E. Stajich, and Corey Nislow. 2017. “Insight into the Recent Genome Duplication of the Halophilic Yeast Hortaea Werneckii: Combining an Improved Genome with Gene Expression and Chromatin Structure.” *G3:*

Genes, Genomes, Genetics 7 (7): 2015–22. http://www.g3journal.org/content/early/2017/05/12/g3.117.040691.

Stephen F. Altschul, Warren Gish, Webb Miller, Eugene W. Myers and David J. Lipman. 1990. “BLAST.” Journal of Molecular Biology. 1990.

Strom, Noah B, and Kathryn E Bushley. 2016. “Two Genomes Are Better than One: History, Genetics, and Biotechnological Applications of Fungal Heterokaryons.” *Fungal Biology and Biotechnology* 3 (4): 1–14. https://doi.org/10.1186/s40694-016-0022-x.

Strope, Pooja K, Daniel A Skelly, Stanislav G Kozmin, Gayathri Mahadevan, Eric A Stone, Paul M Magwene, Fred S Dietrich, and John H McCusker. 2015. “The 100-Genomes Strains, an *S. cerevisiae* Resource That Illuminates Its Natural Phenotypic and Genotypic Variation and Emergence as an Opportunistic Pathogen.” *Genome Research* 25 (5): 762–74. https://doi.org/10.1101/gr.185538.114.

Todd, Robert T, Anja Forche, and Anna Selmecki. 2017. “Ploidy Variation in Fungi: Polyploidy, Aneuploidy, and Genome Evolution.” In *The Fungal Kingdom*, 5:599–618. American Society of Microbiology. https://doi.org/10.1128/microbiolspec.FUNK-0051-2016.

Torres, Eduardo M, Bret R Williams, and Angelika Amon. 2008. “Aneuploidy: Cells Losing Their Balance.” *Genetics* 179 (2): 737–46. https://doi.org/10.1534/genetics.108.090878.

Trivedi, Urmi H., Timothée Cézard, Stephen Bridgett, Anna Montazam, Jenna Nichols, Mark Blaxter, and Karim Gharbi. 2014. “Quality Control of Next-Generation Sequencing Data without a Reference.” *Frontiers in Genetics* 5 (MAY): 111. https://doi.org/10.3389/fgene.2014.00111.

Tůmová, Pavla, Magdalena Uzlíková, Tomáš Jurczyk, and Eva Nohýnková. 2016. “Constitutive

Aneuploidy and Genomic Instability in the Single-Celled Eukaryote *Giardia intestinalis*." *MicrobiologyOpen* 5 (4): 560–74. https://doi.org/10.1002/mbo3.351.

Wajid, Bilal, and Erchin Serpedin. 2012. "Review of General Algorithmic Features for Genome Assemblers for Next Generation Sequencers." *Genomics, Proteomics & Bioinformatics* 10: 58–73. https://doi.org/10.1016/j.gpb.2012.05.006.

Wajid, Bilal, Muhammad U Sohail, Ali R Ekti, and Erchin Serpedin. 2016. "The A, C, G, and T of Genome Assembly." *BioMed Research International* 2016: 6329217. https://doi.org/10.1155/2016/6329217.

Walther, Andrea, Ana Hesselbart, and Jürgen Wendland. 2014. "Genome Sequence of Saccharomyces Carlsbergensis, the World's First Pure Culture Lager Yeast." *G3: Genes, Genomes, Genetics* 4 (5): 1–11. https://doi.org/10.1534/g3.113.010090.

Weib, Clemens L., Marina Pais, Liliana M. Cano, Sophien Kamoun, and Hernán A. Burbano. 2018. "nQuire: A Statistical Framework for Ploidy Estimation Using next Generation Sequencing." *BMC Bioinformatics* 19 (1): 122. https://doi.org/10.1186/s12859-018-2128-z.

Wilkening, Stefan, Manu M. Tekkedil, Gen Lin, Emilie S. Fritsch, Wu Wei, Julien Gagneur, David W. Lazinski, Andrew Camilli, and Lars M. Steinmetz. 2013. "Genotyping 1000 Yeast Strains by Next-Generation Sequencing." *BMC Genomics* 14 (1). https://doi.org/10.1186/1471-2164-14-90.

Zhu, Yuan O., Gavin Sherlock, and Dmitri A. Petrov. 2016. "Whole Genome Analysis of 132 Clinical Saccharomyces Cerevisiae Strains Reveals Extensive Ploidy Variation." *G3: Genes, Genomes, Genetics* 6 (8). https://doi.org/10.1534/g3.116.029397.

Zörgö, Enikö, Karolina Chwialkowska, Arne B. Gjuvsland, Elena Garré, Per Sunnerhagen,

Gianni Liti, Anders Blomberg, Stig W. Omholt, and Jonas Warringer. 2013. “Ancient Evolutionary Trade-Offs between Yeast Ploidy States.” *PLoS Genetics* 9 (3). https://doi.org/10.1371/JOURNAL.PGEN.1003388.

Figure 1: Factors that difficult genome assembly

Ploidy and aneuploidy increase the number of possible states per site. Extreme GC% composition affects the information that different *k*-mers have, and extreme deviations are relatively common in extremophilic organisms. Transposable elements and other forms of repetitive elements increase genome size, affect GC% locally and reduce sequence complexity. Hybridization, heterokaryosis and chimerism introduce two genotypic signals that might be quite divergent, which increases heterozygosity. Finally, contamination introduces undesired sequences with uneven composition, heterozygosity and stoichiometry.

Figure 2: Karyon pipeline

Schematic representation of the steps and program used by Karyon. Red circles represent possible user inputs. Blue boxes represent software used for each step. Orange hexagons represent files generated by the software. Red arrows indicate input to a program, blue arrows represent output of a program. Thicker red arrows represent the standard pipeline, while thinner red arrows represent the different options the user can select to skip some of the steps. These options appear next to the arrow.

Figure 3. Analysis of *Rhizopus microsporus* ATCC62417

A) Variation versus coverage plot reveals the existence of a highly variable portion of the

genome that presents variable heterozygosity levels. B) BlobTools analyses suggest that the genome presents a considerable portion of contaminating sequences. Coverage of the sequences assigned to bacteria is very low when the analyses are performed with other libraries (Data not shown), which proves that the conflicting signal observed in this sample has its origin in a contaminated sequencing library. Results for *R. microsporus* CBS344.5 and var. *rhizopodiformus* B7455 show similar patterns of contamination (data not shown).

Figure 4. Analysis of *Rhizopus microsporus* B9738

A) KAT *k*-mer plot shows very low genome compaction (black area), suggestive of a haploid genome. **B)** Variation versus coverage plot reveals a single main behavior for the genome with regards of its SNP density and coverage. **C)** BlobTools analysis shows no sign of widespread contamination that might be inflating the genome.

Figure 5. Phylogenetic tree of *Rhizopus microsporus* B9738

Phylogenetic tree inferred from OrthoFinder. The *Rhizopus microsporus* species complex is marked in blue. The problematic strain, B9738, is marked in yellow.

Figure 6. Analysis of *Mucor racemosus* B9645

A) KAT *k*-mer plot shows two peaks of coverage considerably affected by genome reduction (black area), suggestive of a highly heterozygous diploid genome. **B)** Variation versus coverage plot reveals a bimodal behaviour for the genome with regards of its coverage, but both peaks appear with very low SNP density. **C)** BlobTools analysis shows no sign of widespread contamination that might be inflating the genome.

Figure 7. Analysis of *Lichtheimia ramosa* B5399.

A) KAT *k*-mer plot shows one peak with considerable genome compaction (black area) suggestive of a diploid genome. **B)** Variation versus coverage plot reveals a unimodal behaviour for the genome with regards of its coverage, presenting a widespread heterozygosity of approximately 3% (maximum density around 30 SNP/Kbp). **C)** Alternative allele frequency shows that all scaffolds present a behaviour very similar to the ideal diploid. **D)** Scaffold length plot shows that, with the exception of a group of very low coverage scaffolds, all the genome presents a uniform coverage.

Table 1:

NCBI Assembly statistics for the analyzed strains. Strains with darker background possessed some property that was affecting assembly quality and was diagnosed using Karyon. Fragmentation in all remaining strains is attributed to low sequencing depth.

Species	NCBI genome size (Mbp)	NCBI number of scaffolds	GeneBank Accesion	Genome size after Karyon (Mbp)	Number of scaffolds after Karyon	Diagnosis
Rhizopus microsporus ATCC 62417	49.6	1386	GCA_900000135.1	40.1	5521	Contamination
Rhizopus microsporus CBS_344.29	49.2	1554	GCA_000825725.1	32.1	3037	Contaminatioon
Rhizopus microsporus B9738	75.1	5266	GCA_000697275.1	71.6	12789	Misidentification
Rhizopus	48.7	4658	GCA_00073	21.8	2176	Contamination

microsporus var. *rhizopodiformus* B7455			8565.1			
Rhizopus delemar Type I NRRL 21789	42.0	3921	GCA_000697155.1	33.4	4824	Unknown
Rhizopus delemar Type II NRRL 21446	38.9	1156	GCA_000738605.1	33.7	5071	Unknown
Rhizopus delemar Type II NRRL 21447	38.7	1177	GCA_000738595.1	28.9	6683	Unknown
Rhizopus delemar Type II NRRL 21477	40.8	1808	GCA_000738585.1	None	None	Unknown
Rhizopus oryzae 99-892	39.1	1168	GCA_000697725.1	29.6	1875	Unknown
Rhizopus oryzae HUMC02	40.3	2313	GCA_000697605.1	None	None	Unknown
Rhizopus oryzae B7407	43.3	4683	GCA_000696915.1	34.7	3720	Unknown
Rhizopus oryzae type I NRRL 13440	43.4	5022	GCA_000697075.1	None	None	Unknown
Rhizopus oryzae type I NRRL 18148	47.5	14653	GCA_000697095.1	None	None	Unknown
Rhizopus oryzae type I NRRL 21396	42.8	4445	GCA_000697115.1	34.2	4115	Unknown
Rhizopus oryzae 99-133	41.5	4317	GCA_000697135.1	27.2	1332	Unknown
Rhizopus oryzae 97-1192	42.9	4566	GCA_000697195.1			Unknown
Rhizopus stolonifer B9770	38	5567	GCA_000697035.1	30.1	6406	Unknown
Mucor circinelloides B8987	36.7	2210	GCA_000696935.1	29.9	4864	Unknown

Mucor indicus B7402	39.8	3117	GCA_000697295.1	32.1	691	Unknown
Mucor racemosus B9645	65.5	6360	GCA_000697255.1	46.0	4444	Hemidiploid
Mucor velutinosus B5328	35.9	2411	GCA_000696895.1	28.2	2743	Unknown
Lichtheimia corymbifera 008-049	36.6	1629	GCA_000697175.1	42.8	3575	Unknown
Lichtheimia corymbifera B2541	36.6	1176	GCA_000697475.1	13.2	3575	Unknown
Lichtheimia ramosa B5399	45.6	3968	GCA_000738555.1	26.6	861	Aneuploid, hybrid
Saksenaea oblongisporus B3353	40.8	1702	GCA_000697495.1	29.7	622	Unknown
Saksenaea vasiformis B4078	42.5	2417	GCA_000697055.1	32.7	1506	Unknown
Cokeromyces recurvatus B5483	29.3	2637	GCA_000697235.1	26.6	5213	Unknown
Syncephalastrum monosporum B8922	29.6	1284	GCA_000697355.1	24.1	5271	Unknown
Syncephalastrum racemosum B6101	29.6	1035	GCA_000696955.1	23.3	311	Unknown
Cunninghamella elegans B9769	31.7	1380	GCA_000697015.1	30.8	5465	Unknown
Apophysomyces elegans B7760	38.5	1528	GCA_000696995.1	29.3	1293	Unknown
Apophysomyces trapeziformis B9324	35.8	1400	GCA_000696975.1	30.1	898	Unknown
Thermomucor indicae-seudaticae HACC 243	29.6	1958	GCA_000787465.1	25.7	4118	Unknown
Parasitella parasitica CBS 412.66 isolate NGI315 ade- mutant	44.9	15637	GCA_000938895.1	23.5	3295	Unknown

801
802
803
804

Figure 1

Click here to access/download;Figure;Fig1.png

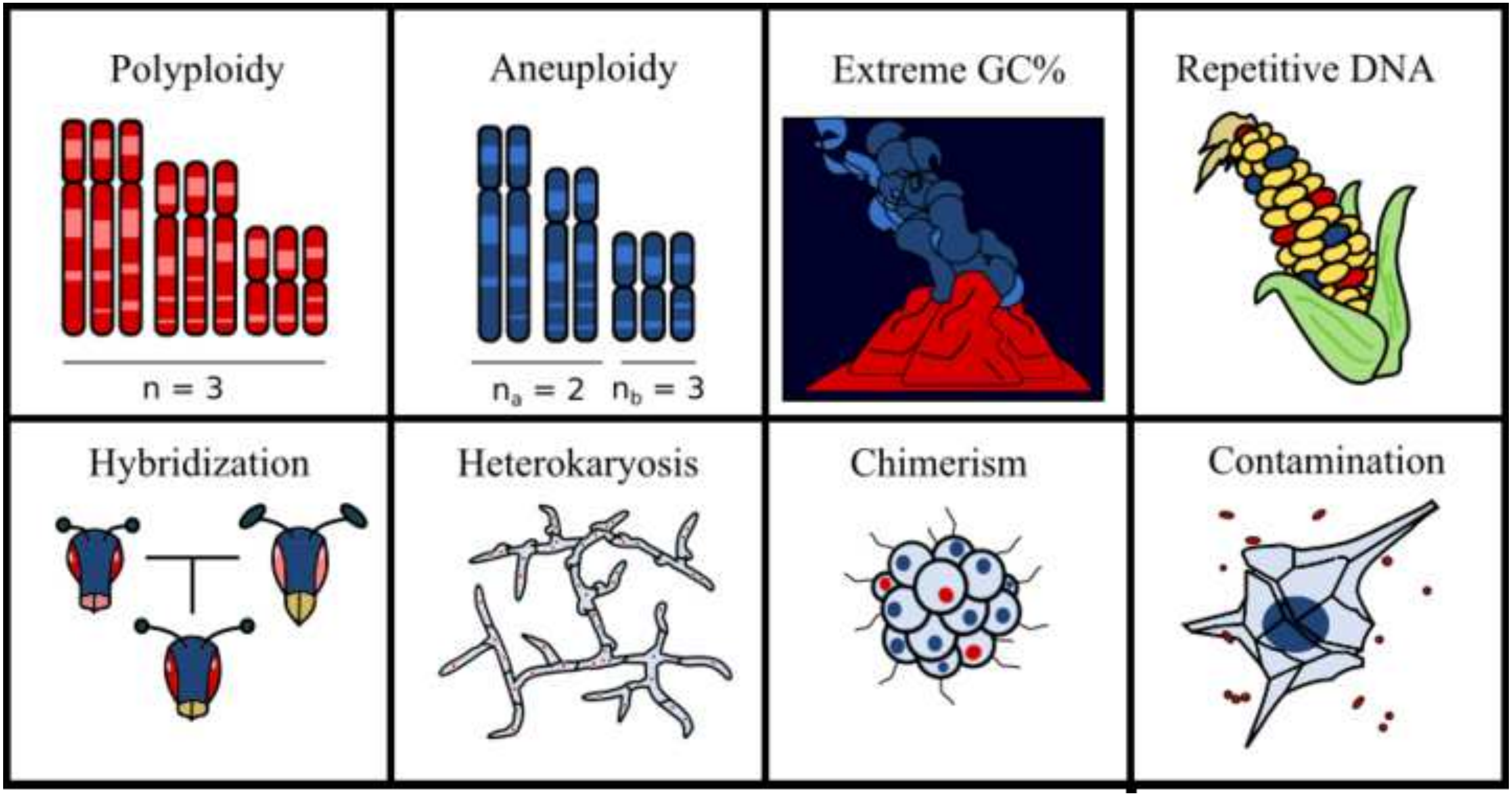

Figure 2

Click here to access/download;Figure;Fig2.png

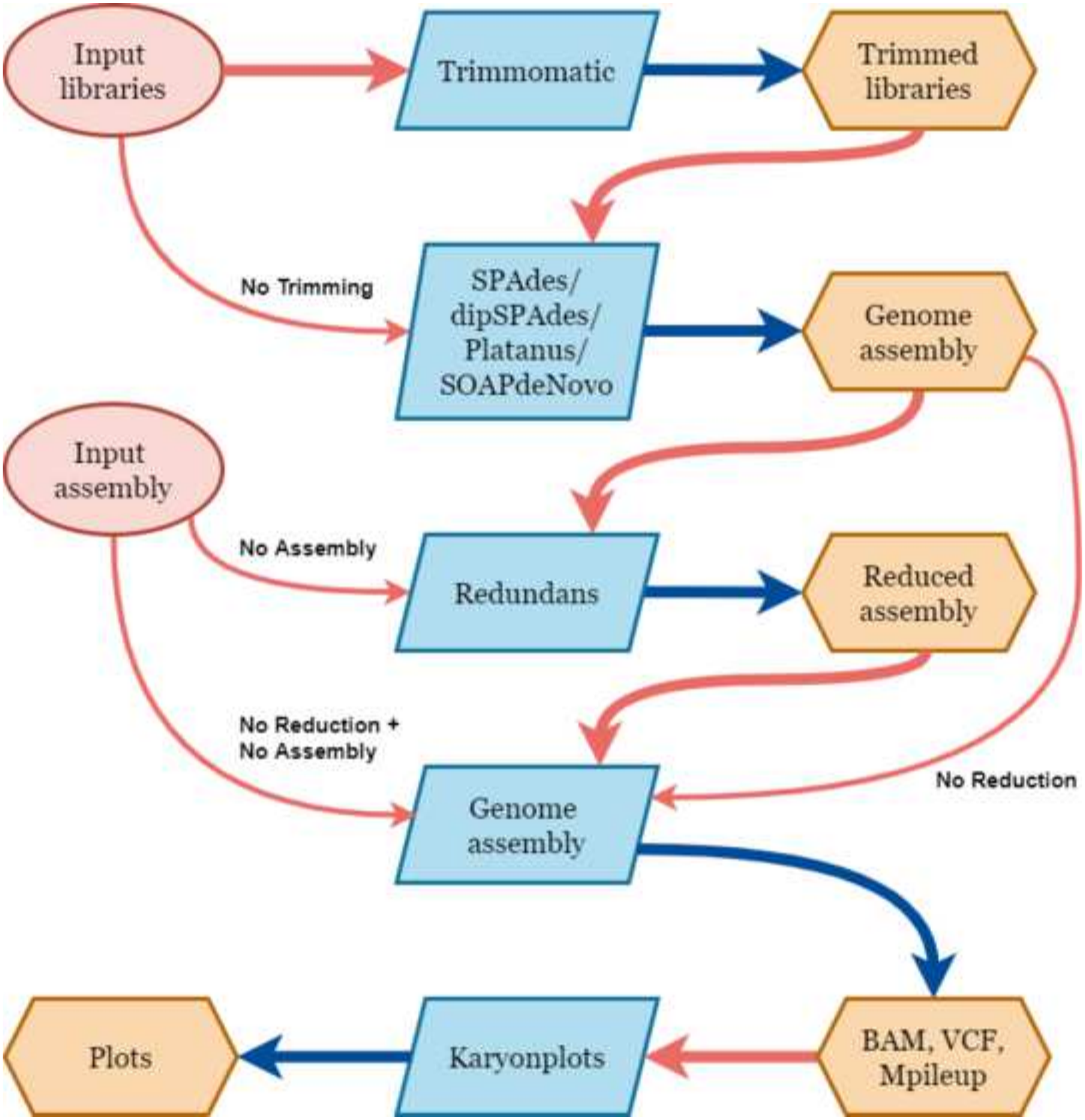

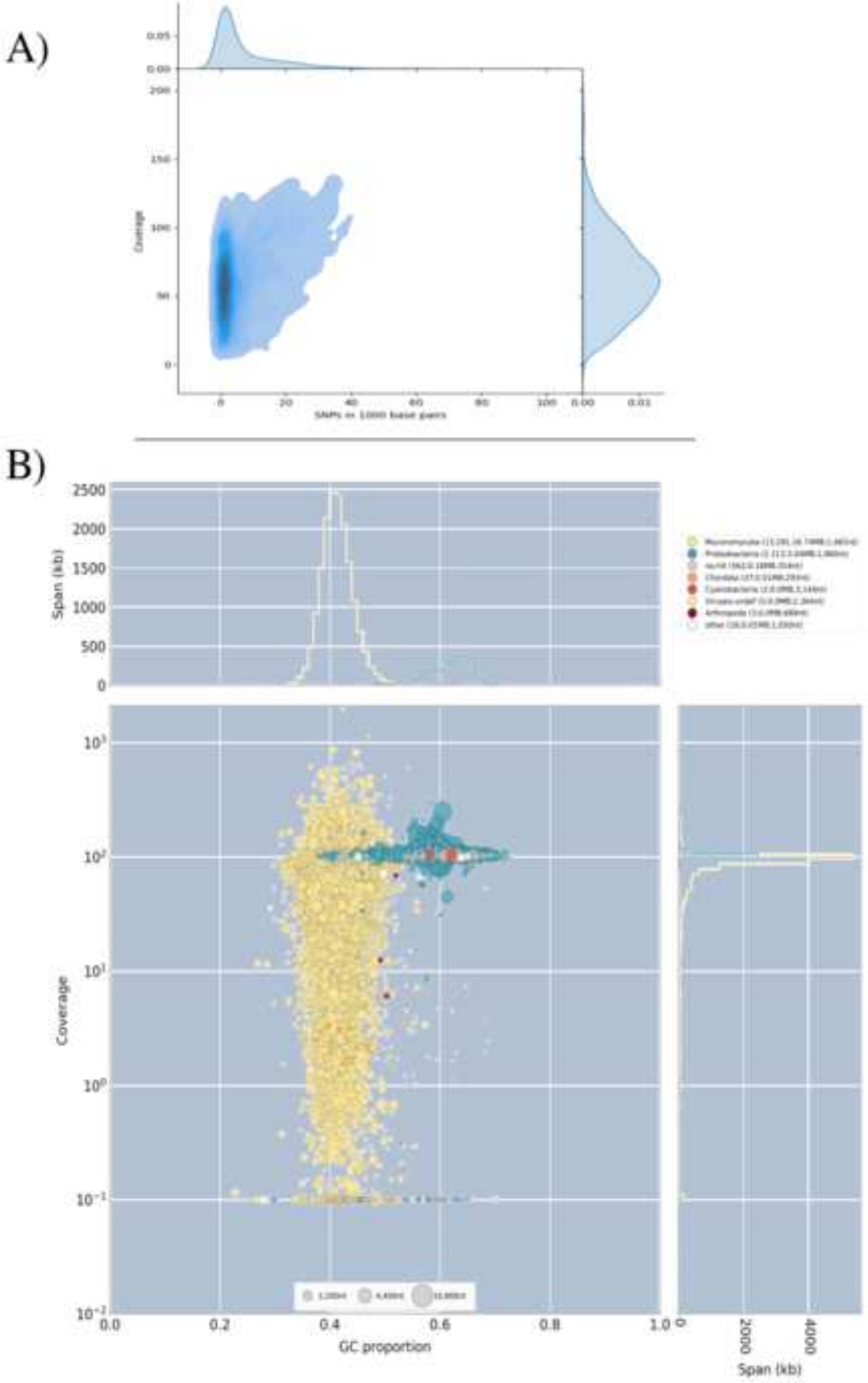
A)
Coverage
SNPs in 1000 base pairs
B)
Span (kb)
Coverage
GC proportion
Span (kb)

Figure 4

Click here to access/download;Figure;Fig4.png

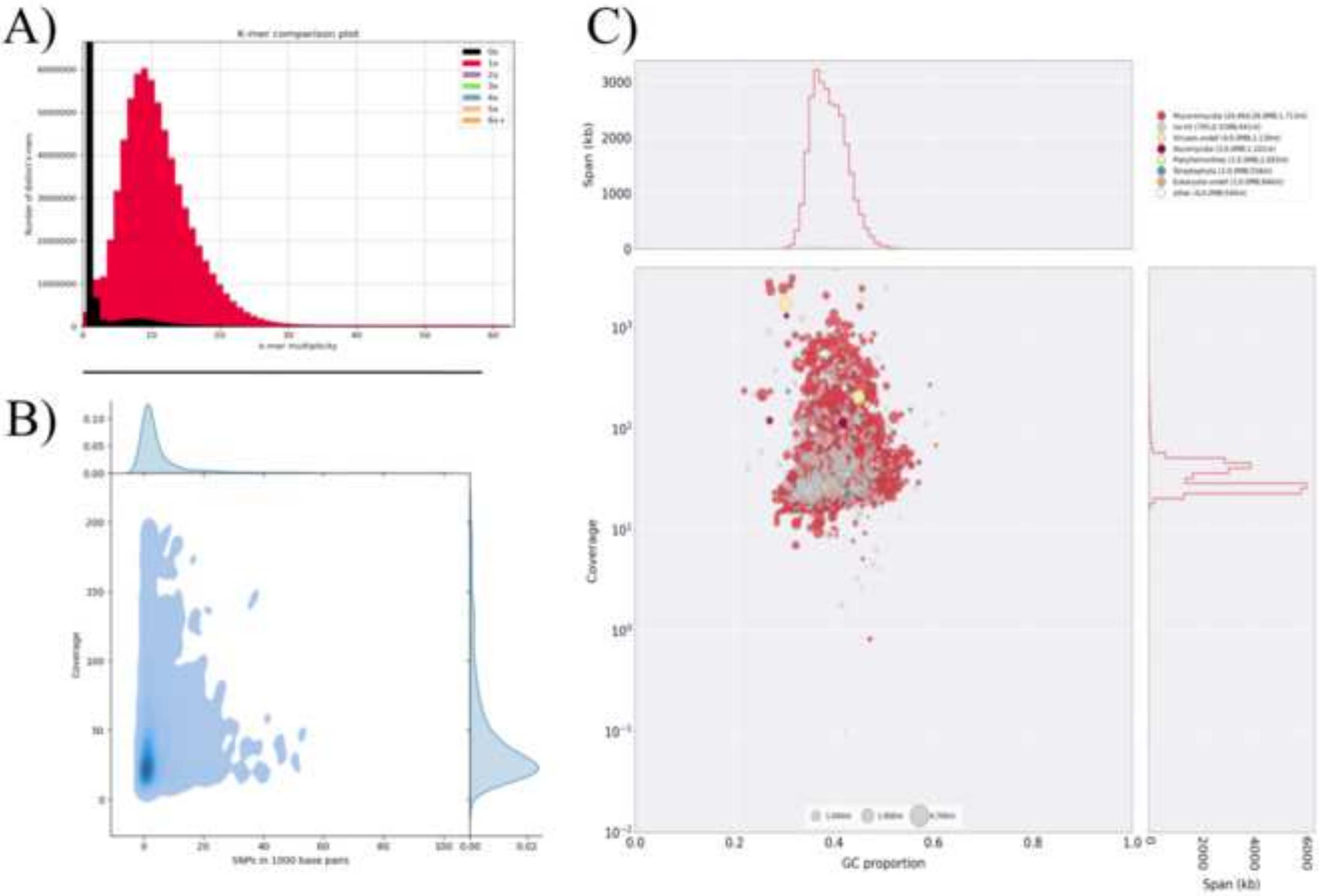

Figure 5

Click here to access/download;Figure;Fig5.png

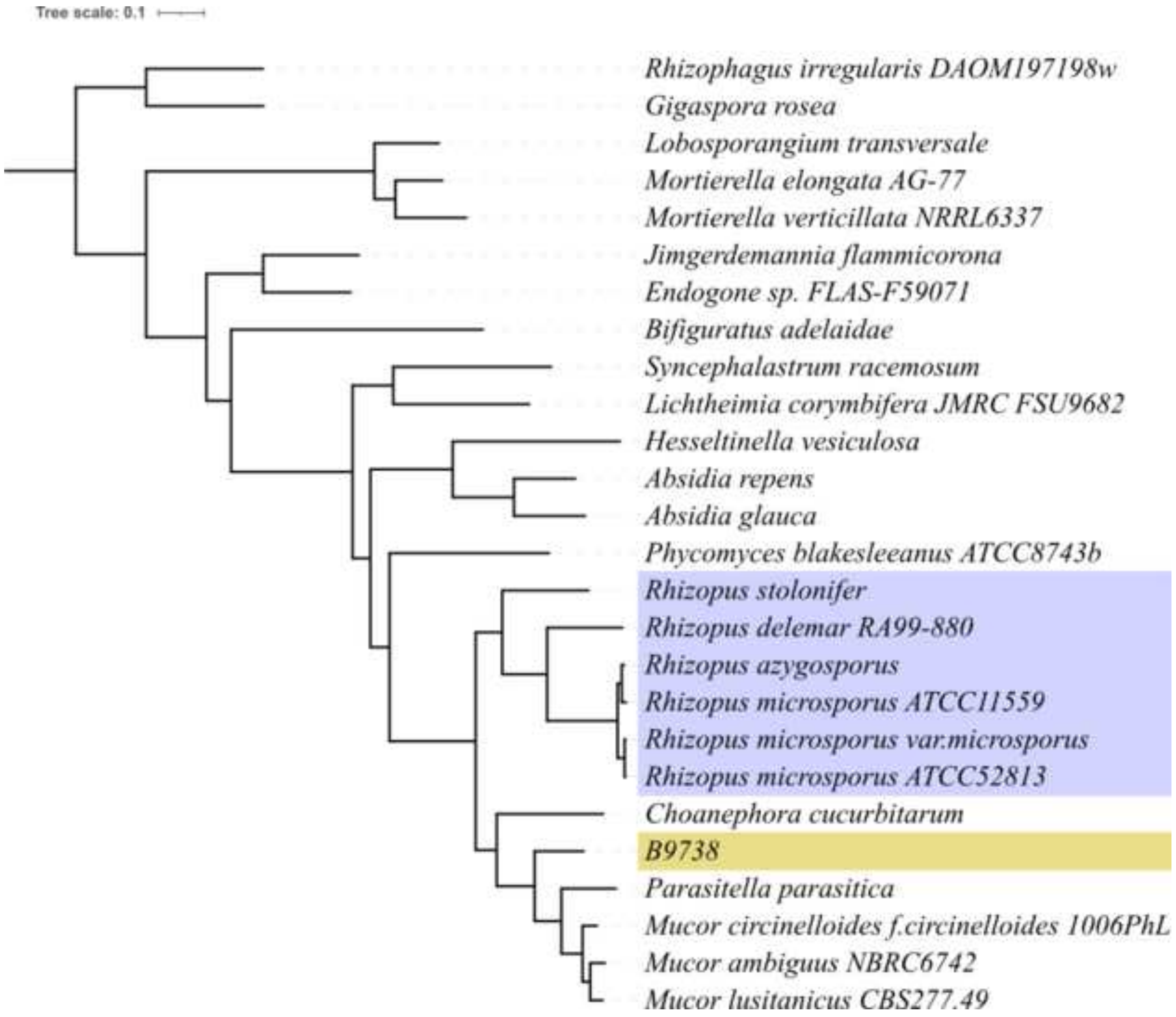

Figure 6

Click here to access/download;Figure;Fig6.png

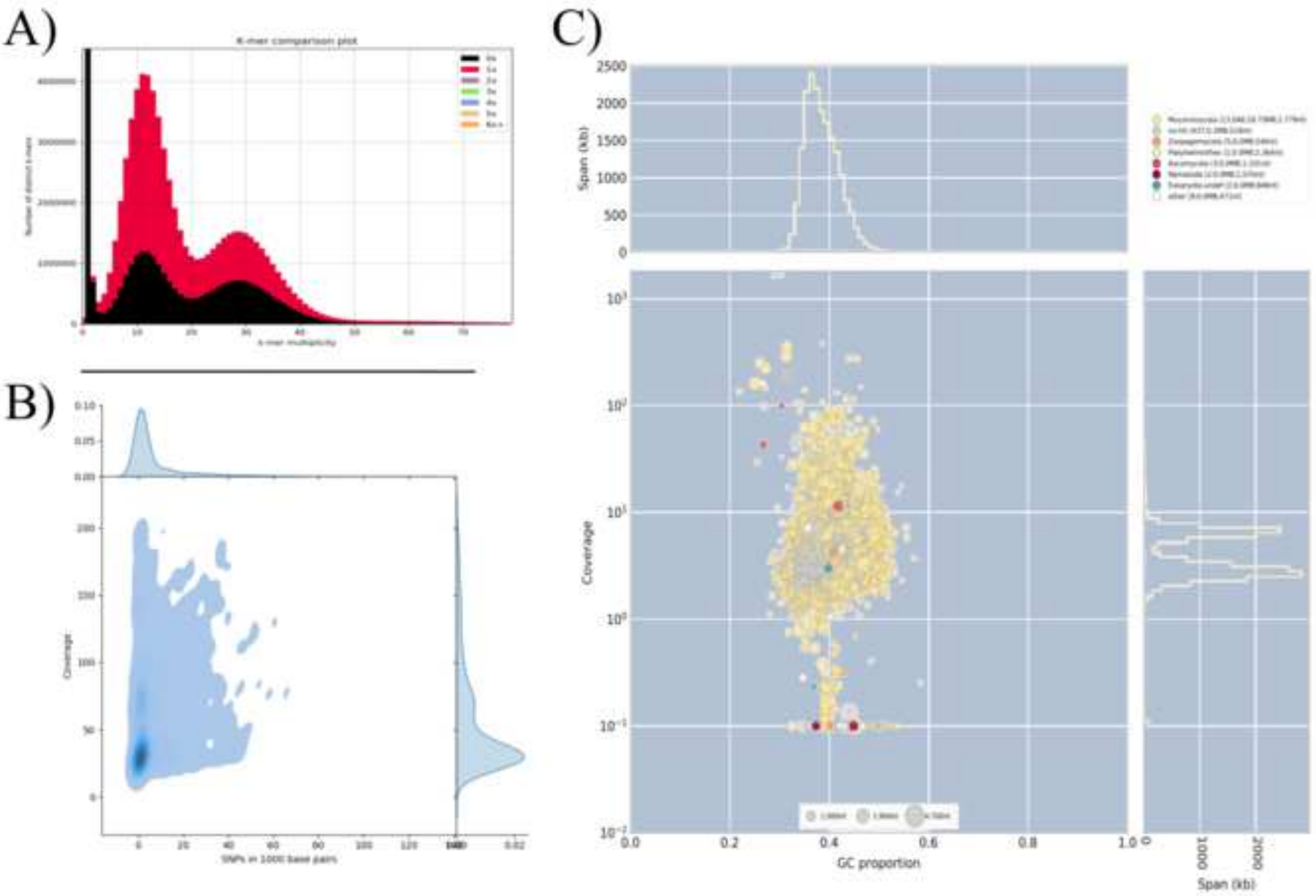

Figure 7

Click here to access/download;Figure;Fig7.png

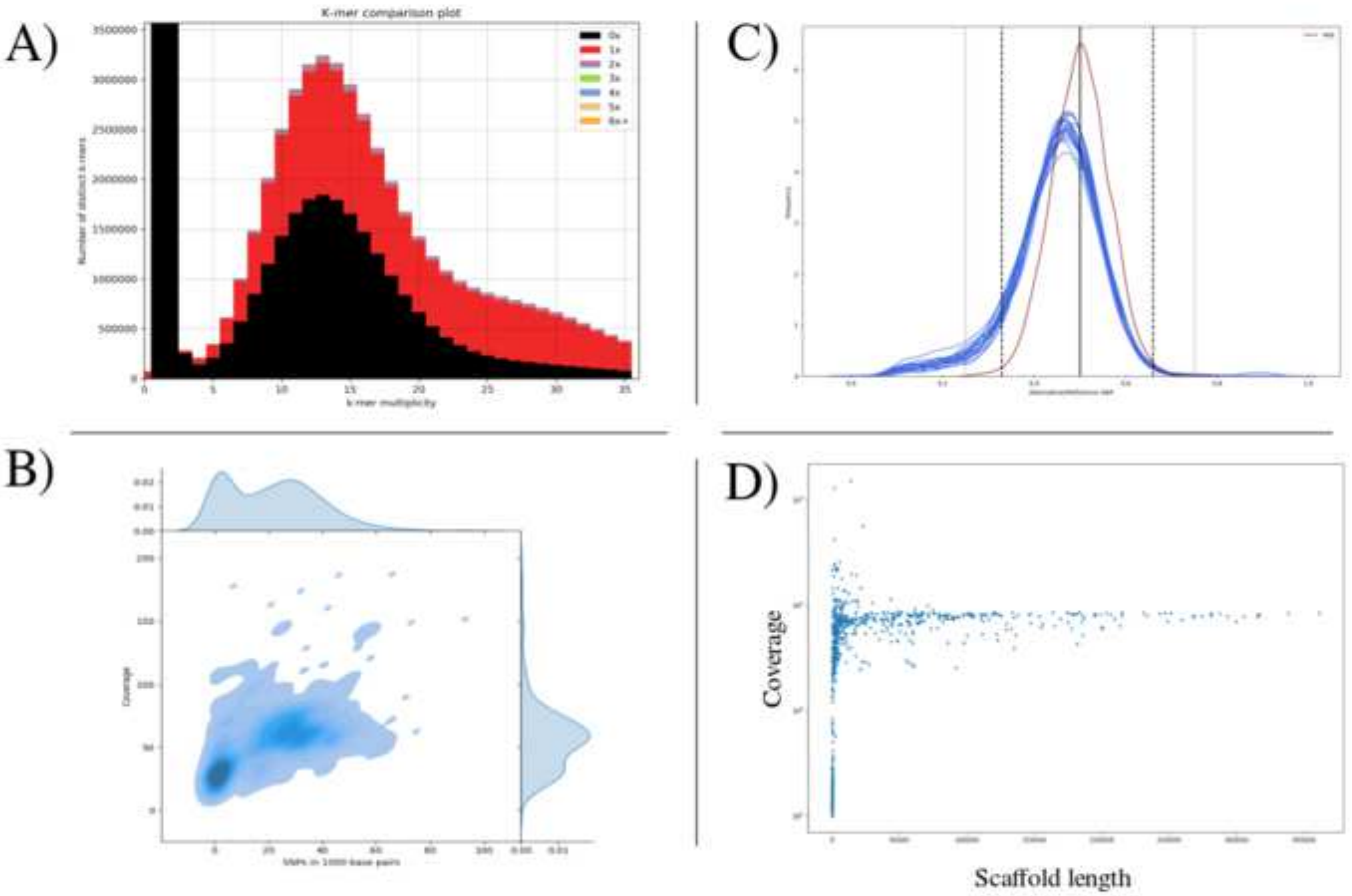

Personal Cover Click here to access/download;Personal Cover;Cover_Karyon.docx

Dear Editors,

Herewith I am sending our article entitled "**Karyon: a computational framework for the diagnosis of hybrids, aneuploids, and other non-standard architectures in genome assemblies"** to be considered for publication as a Technical Note in your journal.

Genome sequencing and assembly has become a routine tasks in many laboratories worldwide thanks to recent technological developments. Many assemblies are obtained using standard sequencing strategies and assembly approaches, which work reasonably well if the target genome does not present complex features. However, the presence in sequenced organisms of certain genomic features such as high heterozygosity, polyploidy, aneuploidy, or heterokaryosis can challenge current standard assembly procedures and result in highly fragmented assemblies. Considering this, it is expected that genome databases contain a non-negligible fraction of low-quality assemblies that result from such type of intrinsic genomic factors. To aid researchers in the identification of such problematic features in genome assemblies and sequencing data, we developed Karyon, a Python-based toolkit that uses raw sequencing data and de novo genome assembly to assess several parameters and generate informative plots to assist in the identification of non-chanonical genomic traits. Karyon can be installed through a docker container and the inclusion of assembly and variant calling pipelines allows users to start a Karyon analysis directly from raw reads.

We illustrate the use of Karyon by diagnosing 35 highly fragmented publicly available assemblies from 19 different Mucorales (Fungi) species. Our results show that 6 (17%) of the assemblies presented signs of unusual genomic configurations, suggesting that these are common, at least within the Fungi.

We look forward to your response.

Yours sincerely,

Toni Gabaldón, PhD
ICREA Research Professor
Comparative Genomics Group
Barcelona Supercomputing Centre (BSC-CNS), and
Institute for Research in Biomedicine (IRB).
Jordi Girona, 29.
08034 Barcelona, Spain
Phone:+34 933160281
http://cgenomics.org
