# Supplementary material for: Karyon: a computational framework for the diagnosis of hybrids, aneuploids, and other nonstandard architectures in genome assemblies
Source: Gigascience. 2022 Oct 7;11:giac088. doi: 10.1093/gigascience/giac088 (PMC9540331; doi:10.1093/gigascience/giac088)

GigaScience

Karyon: a computational framework for the diagnosis of hybrids, aneuploids, and other non-standard architectures in genome assemblies.

--Manuscript Draft--

Manuscript Number:	GIGA-D-21-00155R3
Full Title:	Karyon: a computational framework for the diagnosis of hybrids, aneuploids, and other non-standard architectures in genome assemblies.
Article Type:	Technical Note
Funding Information:	H2020 European Research Council (724173) — Dr Toni Gabaldon<br>Ministerio de Ciencia e Innovación (PGC2018-099921-B-I00) — Dr Toni Gabaldon
Abstract:	Recent technological developments have made genome sequencing and assembly highly accessible and widely used . However, the presence in sequenced organisms of certain genomic features such as high heterozygosity, polyploidy, aneuploidy, heterokaryosis or extreme compositional biases can challenge current standard assembly procedures and result in highly fragmented assemblies. Hence, we hypothesized that genome databases must contain a non-negligible fraction of low-quality assemblies that result from such type of intrinsic genomic factors. Here we present Karyon, a Python-based toolkit that uses raw sequencing data and de novo genome assembly to assess several parameters and generate informative plots to assist in the identification of non-chanonical genomic traits. Karyon includes automated de novo genome assembly and variant calling pipelines. We tested Karyon by diagnosing 35 highly fragmented publicly available assemblies from 19 different Mucorales (Fungi) species. Our results show that 10 (28.57 %) of the assemblies presented signs of unusual genomic configurations, suggesting that these are common, at least for some lineages within the Fungi.
Corresponding Author:	Toni Gabaldon<br>IRB Barcelona: Institut de Recerca Biomedica<br>SPAIN
Corresponding Author Secondary Information:	
Corresponding Author's Institution:	IRB Barcelona: Institut de Recerca Biomedica
Corresponding Author's Secondary Institution:	
First Author:	Miguel A. Naranjo-Ortiz
First Author Secondary Information:	
Order of Authors:	Miguel A. Naranjo-Ortiz
	Manu Molina
	Diego Fuentes
	Verónica Mixão
	Toni Gabaldon
Order of Authors Secondary Information:	
Response to Reviewers:	Dear Toni,<br><br>Your revised manuscript was again with reviewer #3, and unfortunately the reviewer still had problems installing the software. Both using the docker image and the stand-alone code failed, although the reviewer said he had spent an entire working day trying to make it work. Please see his latest report below this email.<br><br>I'm not quite sure what the problem is, and I now double checked with reviewer 1, who

Powered by Editorial Manager® and ProduXion Manager® from Aries Systems Corporation

used your latest version of the code and the manual to successfully install the tool on an Ubuntu system.

As all reviewers like the method, and reviewer 1 confirmed that it runs on his system, we're likely going to accept the Technical Note, but before we proceed I hope you can think of ways to further improve the installation process and manual.

Response: We are sorry that some installation problems remained for reviewer 3. We have taken this issue very seriously and have performed additional efforts to ease the installation process. We have updated the installation script by creating separated conda environments for the different conflicting dependencies. We hope this will make the installation much easier. We have needed the help of one more person from my group, who is now included as a co-author. Details about the new implementations are described in the response to reviewer 3. We have also solved the minor comments from reviewer 1.

After testing the installation, reviewer 1 also mentioned two recommendations that I hope you can address:

1. The package does not include standard test datasets, and I agree with the reviewer it would be good to provide those.

Response: The manual now refers to one of the libraries used for the analyses in the text, that can be downloaded through the SRA toolkit and be used as input for test analyses. We agree that we could have been more clear about how to obtain the test data and we have also added the commands we used to the github README.

2. "One minor comment: the main script "bin/karyon.py" is executable, its first line needs to be sth. like this

#!/bin/env python "

Response: This has been added

Reviewer reports:
Reviewer #3:
In the revised work, the important visible changes are in the actual GitHub repository of the presented tool. Authors removed some of the problematic sections for installation of the tool, but not all. I have (again!) tried to 1. run the docker image, which is at least visible, but not functional. 2. I tried to install the tool locally and still faced many issues. I overcame some, but even after one whole working day and a few shorter attempts, I did not manage to run it through on their test dataset.

Therefore my first and only major comment remains unresolved. The presented tool is still not easily deployable. Not as a docker image, not via local installation.

The idea is great, I thought it's a rather creative way to attack intra-individual ploidy variation. But I simply can't recommend a non-functional pipeline for publication. I am really sorry about this!

Response: We are sorry for the remaining problems. As us and other reviewers successfully used the tool on different systems we think it may be related to some specific incompatibilities. We have therefore extensively tested the installation and performed additional changes to make it easier. We have updated the installation script by creating separated conda environments for the different conflicting dependencies. We hope this will make the installation much more intuitive and simple. For the Docker installation we have updated the docker_install.sh script to install all necessary

	dependencies within the docker container and the Github README to reflect this change. We have also updated the steps on how to set up the Docker environment to account for all the above changes. Moreover, in addition to all the above, we have also updated the pipeline to take advantage of the conda environments added. Users should carefully follow README instructions in the github repository or the Karyon manual, and follow the installation instructions precisely.
Additional Information:	
Question	Response
Are you submitting this manuscript to a special series or article collection?	No
**Experimental design and statistics**<br><br>Full details of the experimental design and statistical methods used should be given in the Methods section, as detailed in our Minimum Standards Reporting Checklist. Information essential to interpreting the data presented should be made available in the figure legends.<br><br>Have you included all the information requested in your manuscript?	Yes
**Resources**<br><br>A description of all resources used, including antibodies, cell lines, animals and software tools, with enough information to allow them to be uniquely identified, should be included in the Methods section. Authors are strongly encouraged to cite Research Resource Identifiers (RRIDs) for antibodies, model organisms and tools, where possible.<br><br>Have you included the information requested as detailed in our Minimum Standards Reporting Checklist?	Yes
**Availability of data and materials**<br><br>All datasets and code on which the conclusions of the paper rely must be either included in your submission or deposited in publicly available repositories (where available and ethically	Yes

Powered by Editorial Manager® and ProduXion Manager® from Aries Systems Corporation

appropriate), referencing such data using a unique identifier in the references and in the "Availability of Data and Materials" section of your manuscript.<br><br>Have you have met the above requirement as detailed in our Minimum Standards Reporting Checklist?	

Manuscript

Click here to access/download;Manuscript;Karyon_revision3_2July2022_Clea

Karyon: a computational framework for the diagnosis of hybrids, aneuploids, and other non-standard architectures in genome assemblies.

Miguel A. Naranjo-Ortiz[1,2,3,4,], Manu Molina[1,2,5], Diego Fuentes[5,6], Verónica Mixão[1,2,,5,6], Toni Gabaldón[1,2,,5,6,7,8*]

1) Centre for Genomic Regulation (CRG), The Barcelona Institute of Science and Technology, Dr. Aiguader 88, Barcelona 08003, Spain

2) Universitat Pompeu Fabra (UPF). 08003 Barcelona, Spain.

3) Clark University. 01610 Worcester, Massachusetts, United States of America.

4) Naturhistoriskmuseum, University of Oslo. 0562 Oslo, Norway.

5) Barcelona Supercomputing Centre (BSC-CNS). Jordi Girona, 29. 08034. Barcelona, Spain.

6) Institute for Research in Biomedicine (IRB Barcelona), The Barcelona Institute of Science and Technology, Baldiri Reixac, 10, 08028 Barcelona, Spain

7) ICREA, Pg. Lluís Companys 23, 08010 Barcelona, Spain.

8) Centro de Investigación Biomédica en Red de Enfermedades Infecciosas. Barcelona, Spain.

*** author for correspondence: toni.gabaldon.bcn@gmail.com**

Abstract

Recent technological developments have made genome sequencing and assembly highly accessible and widely used. However, the presence in sequenced organisms of certain genomic features such as high heterozygosity, polyploidy, aneuploidy, heterokaryosis or extreme compositional biases can challenge current standard assembly procedures and result in highly fragmented assemblies. Hence, we hypothesized that genome databases must contain a non-negligible fraction of low-quality assemblies that result from such type of intrinsic genomic factors. Here we present Karyon, a Python-based toolkit that uses raw sequencing data and *de novo* genome assembly to assess several parameters and generate informative plots to assist in the identification of non-chanonical genomic traits. Karyon includes automated *de novo* genome assembly and variant calling pipelines. We tested Karyon by diagnosing 35 highly fragmented publicly available assemblies from 19 different Mucorales (Fungi) species. Our results show that 10 (28.57%) of the assemblies presented signs of unusual genomic configurations, suggesting that these are common, at least for some lineages within the Fungi.

Keywords

Genome assembly, Heterozygosity, Hybridization, Polyploidy, Aneuploidy

Findings

- We present Karyon, a python-based bioinformatic pipeline that integrates genome

assembly and a series of structural analyses for the diagnosis of problematic genomic structures. Karyon is freely available in github and as a docker container (https://github.com/Gabaldonlab/karyon).

- We applied Karyon to 35 highly fragmented, publicly available genome assemblies to identify putative undescribed deviations in genomic architecture that might have caused problems in a standard assembly process. From 35 assemblies, ten presented features that suggested possible underlying biological factors as the likely cause of the observed assembly fragmentation. Even though our sample size is small and restricted to a single lineage (Mucoromycotina), our results suggest that the number of unreported deviations in genome architecture in Fungi is considerable. This is emphasized if we consider that most researchers that have produced low quality assemblies are unlikely to publish their data.

Introduction

Recent developments in high-throughput sequencing and bioinformatic tools have made the process of sequencing the genome of a new organism a routine task for many laboratories, specially those working on groups with small compact genomes (prokaryotes, fungi, many parasitic lineages). The success of a genome assembly is limited by technical aspects as well as by intrinsic properties of the sequenced genome. A successful assembly depends on the quality, design, and depth of the sequencing libraries which must typically adapt to budget limitations. Naturally, if the sequencing methodology or the computational approaches are inappropriate, the resulting assembly will be poor (i. e. Highly fragmented, incompleted or misassembled). However, additional difficulties might arise independently of the methodology employed, due to intrinsic properties of the genome that interfere with genome assembly algorithms.

Biological factors affecting genome assembly quality

The main intrinsic factors that compromise the success of a genome assembly are the genome size, the sequence heterozygosity, the abundance of low complexity regions (i.e., highly repetitive sequences), as well as the presence of high or uneven ploidy, contaminating sequences or extreme nucleotide compositions (Figure 1).

Genome size impacts computational costs, as many assembly algorithms scale non-linearly (Wajid and Serpedin 2012; Simpson and Pop 2015; Wajid et al. 2016) . Heterozygosity implies the existence of allelic differences within an individual. Standard assembly algorithms have difficulty to differentiate between highly heterozygous regions and distinct but highly similar genomic regions (Hirsch and Robin Buell 2013; Leszek P Pryszcz and Gabaldón 2016) . This in turn results in fragmented assemblies with inflated size compared to empirical measurements, as many of these regions appear duplicated (Leszek P Pryszcz and Gabaldón 2016) , often in short scaffolds. This is particularly problematic in the case of individuals or population that derived from sexual recombination between two or more distinct phylogenetic lineages (Fig 1a), as the component subgenomes often develop structural rearrangements after the split of the two parental lineages. Similarly, repetitive or low complexity genomic regions (Fig 1b) are difficult to resolve without the aid of expensive experimental approaches (e. g. Genetic Maps, Bacterial Artificial Chromosomes, Long Read Sequencing Techniques or Chromosome Conformation Capture), particularly when they span large genomic regions. Duplicated regions introduce multiple possible solutions to the process of scaffolding, increasing assembly fragmentation and computational costs (Hirsch and Robin Buell 2013; Wajid et al. 2016) .

Similarly, ploidy deviations can greatly affect genome assembly. The first possible ploidy deviation is polyploidy (Fig 1c), which is the presence of more than two chromosomes for the majority of the genome. Polyploidy is generally associated to genome heterozygosity, as it increases the number of possible states per site (Aguiar and Istrail 2013; Bonizzoni et al. 2016) . For a diploid site only two states are possible: heterozygous or homozygous, depending on whether the two alleles are different or equal, respectively. For a triploid, however, there are two possible heterozygotic states (e. g. AAB and ABB) and differentiating between them depends on relative frequencies. Allele frequency is affected by stochastic variation, specially if depth of sequencing is low. Aneuploidy (Fig 1d) tends to cause the same problems as polyploidy in assemblies, albeit with the effect being limited only to the aneuploid regions. Because of this, genes present in chromosomes with ploidy higher than two will have a higher likelihood of being unannotated. Animal and plant genomics have traditionally considered aneuploidies as rare events, due to their deleterious effects on many of these organisms, specially during embryonic development. This paradigm is clearly false for many fungal (C. A. Anderson et al. 2015; Berman, Wertheimer, and Stone 2016; Mehrabi, Mirzadi Gohari, and Kema 2017) and protist (Mannaert et al. 2012; Tůmová et al. 2016) lineages. Eukaryotic genomics has only recently started to focus on pangenomes (Golicz, Batley, and Edwards 2016; McCarthy and Fitzpatrick 2019; Sibbald et al. 2020; Naranjo- Ortiz and Gabaldón 2020; Gerdol et al. 2020) , but aneuploidies might be an important confounding factor for these studies. For example, genes located in aneuploid regions are more likely to be missed in annotations, which can inflate estimations of presence/absence variation.

In syncitial organisms, such as filamentous fungi or slime moulds, there is the possibility of coexistence of genetically different populations of nuclei within a cytoplasmic continuum, a condition known as heterokaryosis (Fig 1e) (Maheshwari 2005; James et al. 2008; Strom and Bushley 2016) . Heterokaryosis is functionally similar to ploidy, although with some important differences. First, the relative proportions between heterozygous sites do not necessarily adjust to a simple fraction, as often one population is more abundant that the other. Second, since nuclei divide independently from each other, mitotic or meiotic recombination should be rare. This independency implies that any relative chromosomal rearrangements (i.e., duplications, deletions, translocations, and inversions) between the two nuclear populations, either pre or post union, would remain in nuclear populations for long periods of time. These rearrangements introduce the aforementioned complications in genome assemblies, and some of these might be difficult to differentiate from other chromosomal aberrations. A similar phenomenon is chimerism (Fig 1f), in which the body of an organism is composed by two or more populations of genetically distinct cells. Certain lineages, especially colonial species, might arise by fusion of several genetically distinct individuals (Blanquer and Uriz 2011), but very little is known regarding the effect of chimerism in genome assemblies.

The presence of sequence contamination (Fig 1g) can greatly compromise the quality of the genome assembly (Schmieder and Edwards 2011; Kumar et al. 2013; Trivedi et al. 2014; Laetsch and Blaxter 2017; Lu and Salzberg 2018) . Extraneous sequences introduce noise, create chimeric contigs and might introduce errors in *k*-mer estimations. Highly diverse contaminations (e.g., from the gut microbiota) introduce sequences with highly variable level of coverage, heterozygosity, and composition. On the other hand, highly abundant contaminants (e.g., symbiotic bacteria) are

typically more homogeneous in all these parameters but might still form chimeric contigs and would indirectly reduce the depth of coverage in the main genome. Contaminations reducing the signal of the main genome are particularly problematic for single cell sequencing projects (Huang et al. 2015; Gawad, Koh, and Quake 2016). This is normally prevented by methodological means, but contaminating sequences are intrinsic for certain samples or even organisms, such as the case of symbiotic organisms (e.g., Lichens).

Finally, genomes with extreme compositions, typically very high or low GC content (GC%), can be difficult to assemble (Fig 1h). For these genomes, the information contained by any AT positions is different than the information contained by a GC, as *k*-mers composed of the favoured nucleotide pair will appear at higher frequencies. GC% has a well-documented effect on some sequencing technologies, most notably on the quality of Illumina reads (Benjamini and Speed 2012; Ross et al. 2013) . Fortunately, GC% is easy to measure from raw reads, and some genome assemblers include options specially adapted for these cases (Bankevich et al. 2012; D. Scott and Ely 2014) . Low GC% is typically associated to high abundance of low complexity regions and transposable elements, but extreme GC% is also a hallmark of certain lineages, such as several groups of early diverging Fungi (Naranjo-Ortiz and Gabaldón 2019) . Despite their effects in genome analyses, GC% in eukaryotic genomes is often ignored. For example, neither NCBI nor Mycocosm report GC% in their assembly information statistics, unlike the Genome OnLine Database (GOLD), which has a greater focus on prokaryotic sequences.

If the presence of the factors outlined above is anticipated, specific technical approaches- both experimental and computational- can be used. Contaminating DNA can be identified easily

because sequencing coverage, nucleotide composition and phylogenetic signal is usually different from the main genome and several programs have been developed to identify contaminations (Schmieder and Edwards 2011; Kumar et al. 2013; Trivedi et al. 2014; Laetsch and Blaxter 2017; Lu and Salzberg 2018) . Ploidy can be estimated with cytogenetic techniques, which has been used for animals and plants since the XIXth century. Unfortunately, cytogenetic techniques are time consuming and difficult to interpret for some groups, such as the Fungi. Computational approaches exist to estimate composition and ploidy from sequencing reads (Margarido et al. 2015; Mapleson, Accinelli, Kettleborough, Wright, Clavijo, et al. 2016; Weiß et al. 2018) . Similarly, hybridization can be detected based on phenotypic traits (intermediate phenotypes and hybrid vigor). Again, this is not feasible for most microbial eukaryotes due to the lack of easily identificable phenotypes. Genomes of hybrid organisms are heterozygous, and some genome assembly software have been designed to be able to handle this situation (Kajitani et al. 2014; Safonova, Bankevich, and Pevzner 2015; Pryszcz and Gabaldón 2016) , but proper identification of hybrid lineages cannot be done without adequate population and phylogenetic analyses.

Thus, biological factors affecting genome assembly quality increase the overall costs of a project and require expertise that might not be available. Given the difficulty of performing analyses on low quality assemblies, it is likely that published genomes are biased in favor of organisms with genomic characteristics that makes them easier to work with. In contrast, genomic projects that choose organisms with non-standard genomic architectures are more likely to suffer methodological obstacles that delay or even prevent analyses. Our inability to work around non-standard genomic architectures distorts our perception of biological phenomena, relegating them to mere oddities.

Results

The Karyon toolkit

To aid in the identification of these non-canonical genomic architectures, we developed Karyon, a python-based toolkit that assesses several parameters of sequencing data and their derived assemblies that are common indicators of different intrinsic genomic features leading to poor assemblies. Karyon is comprised of different modules that can be used independently or sequentially. Karyon is written in Python 3 and freely available to download as a Docker build or as a standalone project in https://github.com/Gabaldonlab/karyon.

Karyon integrates Trimmomatic (Bolger, Lohse, and Usadel 2014) as an optional step to eliminate low quality positions and adapters from sequencing reads. It then uses that input to generate a *de novo* assembly using SPAdes v3.9.0 (Bankevich et al. 2012) , dipSPAdes v3.9.0 (Safonova, Bankevich, and Pevzner 2015) , Platanus v1.2.4 (Kajitani et al. 2014) or SOAPdenovo2 v2.04-r240 (Luo et al. 2012) . Karyon then uses the *de novo* assembly to generate a reduced assembly using Redundans (Leszek P Pryszcz and Gabaldón 2016) . Redundans is a pipeline that collapses assembly fragments with high similarity to create an artificial haploid genome assembly. This assembly is then used as reference to map the original sequencing reads using BWA-MEM (Li 2013) and generate a variant calling file with GATK v4.1.9.0 (McKenna et al. 2010) . A battery of analyses is then performed on the sequencing libraries, the assemblies, and the maps of coverage and genetic variation to generate plots that will aid in the diagnosis of the genomic structure. Figure 2 summarizes the pipeline.

Karyon uses the K-mer analysis toolkit (KAT) (Mapleson, et. al. 2016) to provide a *k*-mer (all possible sequences of length *k*) spectrum analysis as part of its report. From this analysis it produces frequency histograms representing coverage versus *k*-mer counts. These plots inform on ploidy and heterozygosity of a genome. In a haploid genome *k*-mers of enough size will appear either one or zero times, with unique *k*-mers having an average coverage roughly equal to the average global depth of coverage. Deviations from these patterns suggest alternative architectures. For instance, the presence of two peaks in the *k*-mer plot typically indicate a genome that is totally or partially non-homozygous diploid. To complement these analyses and provide further information on the features of the genome, Karyon assesses scaffold length distributions, relationships between scaffold length and coverage, sliding-window analysis of coverage and genetic variation, as well as allele-frequency distributions per scaffold (Figure 2). In addition, Karyon uses nQuire (Weiß et al. 2018) to estimate the likelihood of different ploidy levels in sliding windows per scaffold. Karyon also incorporates BUSCO completeness analysis (Simão et al. 2015) with automatic taxonomic assignment. Altogether, the interpretation of these analyses can be used to detect polyploidies, aneuploidies, hybridizations, heterokaryosis, large segmental duplications, unusual DNA composition or the presence of symbiont or contaminating sequences. Karyon generates a report file that summarizes the results of these analyses and raises some warning messages in case certain metrics are problematic, such as low BUSCO completeness, low percent of mapped reads or extreme GC% values.

Karyon generates a series of original plots that aim to provide valuable information regarding the architecture of the problem assembly:

1. **Scaffold length plots.** (Fig 3a) These plots represent the distribution of scaffold length

through a bar plot, where each value represents a single scaffold versus its length, with all scaffolds sorted from shortest to longest. Karyon generates the results in linear and logarithmic distribution. Very short scaffolds (shorter than 1Kbp) might introduce noise in the analyses and might be interesting to just filter them out.

2. **Scaffold versus coverage.** (Fig 3b) Karyon generates a scatter plot representing the average coverage versus length for each scaffold in the assembly. Quite often short scaffolds have different coverage from most of the genome, which might be indicative of contamination or repetitive regions.
3. **Variation versus coverage plot**. (Fig 3c) This plot allows the user to observe overall patterns across the whole genome. It uses a Kernel Density Estimation over a cloud of dots. Each dot represents the number of SNPs (X axis) versus the average coverage (Y axis) in a window of the genome, typically 1Kb. Presence of more than one population of dots is indicative of genomes with dual behavior, such as aneuploidies or loss of heterozygosity.
4. **Fair coin plot.** (Fig 3d) This plot represents the proportion of alternative vs. reference SNP for the whole genome and for each individual scaffold. Vertical lines indicate expected frequencies of 0.5, 0.33 and 0.25, corresponding to ideal diploids, triploids and tetraploids, respectively. An expected frequency is drawn, which is based on a per-site simulation of proportions assuming ideal 0.5 relative frequencies and random sampling equal to the coverage of the site. The plot is generated for the whole genome, as well as per scaffold.
5. **nQuire per scaffold plot.** (Fig 3e) Karyon will run nQuire across sliding windows of defined length (by default 1Kbp) across different scaffolds. The plot for each scaffold contains five subplots. The first three represent the nQuire score for diploid, triploid, and tetraploid for that particular window. This allows the user to visualize patterns of

aneuploidy per scaffold, especially with regards to diploid and triploid regions. The fourth and fifth subplot represents the location and coverage of SNPs across the scaffold. A color code is assigned to represent the density. Since nQuire requires information of SNPs, homozygous regions cannot be assessed and will appear as missing data.

Each of the steps is optional and can be controlled with flags in the main script. Additionally, the script uses a configuration file, that allows to define the options of each of the dependency programs. This configuration file is automatically created during the installation and can be modified with any text editor. We encourage the user to make a copy of the original configuration file for future modification. Installation is fully automated, requiring no user input during the process.

Genomic survey in the Mucorales (Fungi)

To showcase the use of Karyon, we undertook an analysis of deposited fungal genomes in the order Mucorales. Fungi are in a particularly privileged position to assess the impact of non-canonical genomic architectures in genome assemblies. Fungi generally have small and compact genomes and can be often cultured under axenic conditions. As a result, the amount of sequenced fungal genomes is now in the order of thousands, including multiple strains for many species. Even more, comprehensive efforts to obtain a balanced coverage of the existing fungal diversity are ongoing, such as the 1000 fungal genomes (Grigoriev et al. 2014) and the 1000 yeast genomes initiatives (Wilkening et al. 2013; Strope et al. 2015; Zhu, Sherlock, and Petrov 2016; Peter et al. 2018) . Thus, fungi provide an excellent system to study the incidence of different genomic accidents in evolution (Gerstein and Berman 2015; Berman, Wertheimer, and Stone 2016; Todd,

Forche, and Selmecki 2017) . Despite this, the quality of fungal genomes is often sub-optimal, and databases are riffed with highly fragmented assemblies. Genomic factors such as those discussed above might complicate genome assembly and be responsible for this observed fragmentation, at least partially. Considering this, we hypothesized that genome databases must contain a fraction of low-quality assemblies from fungal organisms that are caused by intrinsic genomic factors. If that is true, reanalysis of the raw data should lead us to describe novel genomic accidents and obtain a minimum estimate of their relative abundance.

We thus applied Karyon to a set of 35 publicly deposited genomes from the fungal order Mucorales. Our results suggest that non-standard genomic organizations are not rare, and that future studies on other groups are likely to uncover many new cases. We selected the order Mucorales because this group comprises several described examples of whole-genome duplication, both ancient and recent (Ma et al. 2009; Corrochano et al. 2016) . Many sequenced members of the clade come from clinical samples, an environment that is known to promote the emergence of different genomic accidents (Schoenfelder and Fox 2015; Todd, Forche, and Selmecki 2017; Mixão and Gabaldón 2018) . Additionally, several represented species included two or more sequenced isolates, allowing to get a glimpse at their intra-specific diversity. We obtained 35 genome assemblies from 19 different Mucorales species deposited in GenBank between January 1st 2005 and December 31th 2015 (Table 1). For 4 of the species, dipSPADes was unable to generate an assembly.

Karyon was run using the complete default pipeline. Most of the analyzed genomes (27, 79.4%) presented very low levels of heterozygosity and a relatively homogeneous coverage across the

genome, suggesting that those strains are haploid or, if presenting higher ploidy, extremely homozygous. Fragmentation in these cases might be caused by insufficient coverage, presence of repetitive regions or some other methodological constraints. However, our pipeline uncovered cases that produced anomalous results in the different Karyon tests. Many zygomycetous fungi exhibit low or very low GC%. In our dataset, 5 species showed GC% below our threshold of 35%, with several others approximating that value. Additionally, some of the analyzed genomes show signs of ploidy anomalies or contamination. Below we describe these cases and propose a plausible scenario to explain each of the obtained results based on the data obtained from the Karyon pipeline.

***Rhizopus microsporus* species complex**

At the time of this study, the NCBI database had deposited sequences for eight *Rhizopus microsporus* strains. Interestingly, three of them presented a genome size estimated around 25Mbp; four of them had a genome size close to 50Mbp; and one presented a genome size of 75Mbp. Only the three strains with a genome size of 25Mbp had sufficiently good assemblies considering they were based on short read, with a scaffold number below 1000, and thus were not selected for further analyses. Additionally, the raw libraries for one of the strains presenting 50Mbp genome assembly size (*Rhizopus microsporus* var. *chinensis* CCTCC M201021) were not publicly available and thus could not be part of the survey. For the remaining three strains with genome size close to 50Mbp (ATCC62417, CBS344.29 and var *rhizopodiformis* B7455), our *de novo* assembly pipeline recovered a genome size of approximately 40Mb, which is smaller than the assemblies deposited in NCBI (Table 1). The heterozygosity distribution in these assemblies shows that most of the genome presented a relatively uniform behavior with low heterozygosity.

In all three cases, though, a considerable proportion of the genome appears with a highly variable coverage and increased heterozygosity (Figure 4). For these three strains, BlobTools (Laetsch and Blaxter 2017) shows widespread bacterial contamination (Figure 4b) and thus we conclude that contamination might be responsible for the observed assembly fragmentation.

The remaining strain, B9738, showed a surprisingly large genome size in both the assembly deposited in NCBI (75Mbp), and the one reconstructed here (71Mbp). The genome of *R. microsporus* B9738 presents an extremely low level of heterozygosity and a very homogeneous coverage. *K*-mer spectrum also shows just one very clear peak. All in all, all this suggests that B9738 is haploid (or a highly homozygous diploid), despite presenting a 3-fold increase in genome size as compared to other strains of the same species (Figure 5). Augustus gene prediction returned a total of 21,300 gene models, which is an unusually large number for a filamentous fungus. As a reference, the seven genomes in the Rhizopodaceae, to which *Rhizopus* belongs, available in Mycocosm range from 25 to 46 Mbp and from 10,781 to 17,676 annotated genes. Contamination analysis does not suggest the presence of widespread contamination that could explain such over-inflated genome (Figure 5). For this reason, we suggest that B9738 might be a misidentified strain that does not belong to the *R. microsporus* species complex. Indeed, phylogenomic analyses recover B9738 as sister to a clade containing *Mucor* and *Parasitella*, rather than allied with the rest of the *Rhizopus microsporus* species clade (Figure 6), thus supporting a misidentification. It is noteworthy that no sequenced species of either *Mucor* or *Parasitella* have genomes above 49Mbp or with more than 15,000 genes, at least from the available genomes in Mycocosm.

***Mucor racemosus* B9645**

Analyses on *Mucor racemosus* B9645 depicted a genome with a dual behavior. The distribution

of heterozygosity and coverage showed two peaks with very low heterozygosity but with different coverage (Figure 7b). This was further confirmed by the *k*-mer spectrum analysis, which revealed two clear peaks (Figure 7a). The genome available in NCBI is 65.5Mbp-long, noticeably larger than the 45.9Mbp we recovered in our analyses (Table 1). The reduction step of Redundans cannot explain this difference, as the assembly size prior to this step is already 46.8Mbp, very close to the final result. Our analyses suggest that contaminating sequences are very minor and do not explain the observed pattern (Figure 7). We hypothesize that *M. racemosus* B9645 is a hemidiploid, which presents a portion of its genome in haploid state, and other portion in a highly homozygous diploid state. Due to the low heterozygosity exhibited by this strain, the observed genome architecture might have arisen by either autopolyploidization followed by chromosome loss or by chromosomal duplications. Additionally, GC% for this species was only 32.6%.

***Lichtheimia ramosa* B5399**

The Karyon assembly for this genome was only 26.6Mbp, much smaller than the NCBI assembly (45.6Mpb long, Table 1). Unlike other genomes, our assembly presented a considerable improved quality, going from 3,968 scaffolds and N50 of 33,650 in the NCBI assembly to 861 scaffolds and N50 of 133,635 in our own assembly. *L. ramosa* presents a heterozygosity level around 3% in its diploid peak (Figure 8). All considered, we propose that *L. ramosa* B5399 is a mix of haploid and diploid with high heterozygosity, likely resulting from mating between two distantly related strains followed by genomic aneuploidization.

Methods

Sequencing data

We downloaded raw data from libraries deposited at Short Read Archive (SRA) of those species in the Mucorales with a highly fragmented assembly (>1,000 scaffolds), which included at least one paired-end Illumina library larger than 1Gb after quality filtering (Table 1), to ensure at least a decent coverage. Since most of our genomes have typical assembly sizes around 40Mbp, this measure ensures a bare minimum average coverage of 20. All available sequencing libraries were used for all the analyses.

De novo gene annotation

We used Augustus v3.1.0. (Brudno et al. 2003) to obtain a *de novo* gene prediction using the *Rhizopus oryzae* Generalized Hidden Markov Model included in the default installation of Augustus.

Contamination detection

For each of the conflictive assemblies, we generated an Augustus prediction. Then, we used Blastp (Stephen F. Altschul, Warren Gish, Webb Miller 1990) to query the whole proteome against Uniref100 (Consortium 2014) . Since the genomes come from public databases, their own proteins should appear as hits and thus we retrieved the 10 best hits. We have used these hits to assign a taxonomic profile. Additionally, we have used the predicted Augustus CDS to map sequencing reads with GATK. With both the taxonomic profile and the variant calling file, we have run BlobTools (Laetsch and Blaxter 2017) to identify the presence of widespread contamination in the sequencing libraries.

Phylogenomic analyses

In order to identify the phylogenetic position of *R. microsporus* B9738 we used the Augustus gene prediction and the proteome of 24 other zygomycetes to run OrthoFinder v.2.3.3 (Emms and Kelly 2019) with the flags -S blast and -m msa.

Discussion

As genome sequencing has moved away from model organisms, it has become apparent that many possible genomic architectures are possible, and many do exist in a wide range of organisms. Most of these genomic accidents are difficult to identify from sequencing data alone. As far as we know, Karyon is the first software developed with the intention of performing reference-free analyses for the presence of a wide array of genomic factors affecting the quality of *de novo* genome assembly. We have designed this software to be easy to install and use, with the possibility of installation from both GitHub and Docker.

Despite the success in the implemented strategy, we consider our software has several limitations. Karyon requires an assembly step and variant calling protocol, for which some default options are included. However, the included programs might not suit every need. For example, extremely large genomes might require alternative assemblers that are not included in our pipeline, or some users might prefer a different set of programs for the variant calling protocol. For those cases Karyon can still be used as independent steps (Figure 2). Karyon is designed to work without any preexisting data, which limits the information it can predict. Comparing different genome assemblies, specially if at least one of them has good quality, can help detect many of these alternative genomic architectures and some others that are outside the capabilities of Karyon. If other reference genomes are available, tools like QUAST (Gurevich *et al,* 2013) can generate similar analyses to Karyon with higher accuracy and speed.

Despite the increasing use of long-read technologies for assembly purposes, a large amount of genome assemblies available in public databases have been generated exclusively from short reads. As of October 2021, NCBI SRA contains 173087 DNA libraries for Fungi, of which 157144 are Illumina short reads, and only 6163 are long reads (4700 PacBio and 1463 Nanopore). At this moment, the pipeline assumes the use of at least one Illumina paired-end sequencing library. Because of this, we recommend the use of other genome assemblers if other sequencing technologies (i.e., Nanopore or PacBio long reads) are to be used, and the same goes for variant calling protocols.

We provided a practical example of the usage of Karyon on a publicly available set of fungal genomes from the order Mucorales. While the majority of analyzed assemblies show no sign of any of the considered biological conditions, we were able to effectively find underlying non-standard genomic architectures that had been previously unnoticed in these assemblies. These results suggest that many authors do not take into consideration this kind of genomic accidents, which in turn greatly hampers the results that might be obtained from them.

How common are these non-standard genomic architectures? Our results suggest that they might be quite abundant, although so far they are restricted to a limited selection of species within a narrow clade of Fungi. As such, these genomic anomalies might, or might not, be common in other lineages. However, we consider that there are three important arguments in favor for considering our dataset an underestimation of the abundance of unorthodox fungal genomes, even within the limited taxonomic range we have selected. The first one is the fact that fungal biomass used for

DNA extraction and subsequent sequencing typically comes from cultures. This implies an important ecological step in which the fungus grows at optimal speed and in the absence of most stressors. Aneuploidies, polyploidies, and other similar genomic rearrangements are common in the presence of stressors (C. A. Anderson et al. 2015; Berman 2016; Berman, Wertheimer, and Stone 2016; Todd, Forche, and Selmecki 2017) , but seem to be outcompeted by euploid cells under optimal growth conditions (Kumaran, Yang, and Leu 2013; Zörgö et al. 2013; A. L. Scott et al. 2017) . Hence, isolates growing in rich medium will be selected to lose most chromosomal aberrations they might present. Analogously, many of these chromosomal aberrations might exist in nature but are unable to grow on optimal medium. The advance of environmental sequencing and single-cell based technologies might cast some light in this matter in coming years. Supporting this argument, Ahrendt et al. sequenced several environmental isolates of zoosporic and zygomycetous microfungi using these techniques and found several aneuploids and polyploids (Ahrendt et al. 2018) . The frequency of unconventional genomic architectures is very likely lineage dependent. While some of these are well known, such as the dikaryotic phase in Agaricomycetes or the macro and micronuclei of ciliates, strange genomic architectures might be common in more obscure lineages. This not only represents a yet-to-know facet of the biology of these organisms, but it could potentially complicate their study. The third factor to consider is purely human. The datasets we have analyzed were uploaded by researchers who considered they were good enough to be uploaded to a public repository. Thus, it is to be expected that many more low-quality assemblies would have never been never deposited and sit forgotten in the disks of laboratory computers, if not discarded completely.

Even if we consider these possible biases as negligible, our results recover a significant fraction of

publicly available genomes with unorthodox genomic configurations. These have been correlated in many fungal groups with adaptation to novel environments (Lenassi et al. 2013; Kravets et al. 2014; Sinha et al. 2017) , resistance to antifungals (Harrison et al. 2014; M. Z. Anderson et al. 2017) , pathogenic capabilities toward both animals (W. Li et al. 2012; Morrow and Fraser 2013; Gerstein et al. 2015; Mixão and Gabaldón 2018) and plants (Garbelotto et al. 2004; Depotter et al. 2016) and adaptation to industrial settings (S. a. James et al. 2005; Louis et al. 2012; Borneman et al. 2014; Walther, Hesselbart, and Wendland 2014; Peter et al. 2018; Avramova et al. 2018) . Beyond that, contamination in sequencing libraries is a problem that can affect any assembly project and might mislead downstream inferences if left unaddressed. Validation of published results goes far beyond the interest of discovering overlooked findings. Comparative genomic studies are limited in their scope and reliability by the quality of assembly and annotation of the genomes, factors that can be greatly compromised by these biological factors. Comparative studies commonly require the use of flagship genomes that represent a given taxon. Often, this generates a chronology of comparisons versus the reference that shapes the perspective on the group. As such, artifacts, and errors in strategic genome assemblies, such as reference strains or strains in groups with few represented species, might have a domino effect impacting future studies. Long-read sequencing technologies, which are increasingly being used for genome assembly projects, hold the promise of providing much more information that could be used to resolve many of these unorthodox genomic architectures. However, these approaches require novel computational approaches to fully employ their potential.

Conflict Statement

The authors state that they have no conflicts of interests.

Acknowledgements

TG group acknowledges support from the Spanish Ministry of Science and Innovation for grant PGC2018-099921-B-I00, cofounded by European Regional Development Fund (ERDF); from the Catalan Research Agency (AGAUR) SGR423; from the European Union's Horizon 2020 research and innovation programme (ERC-2016-724173); from the Gordon and Betty Moore Foundation (Grant GBMF9742) and from the Instituto de Salud Carlos III (IMPACT Grant IMP/00019 and CIBERINFEC CB21/13/00061- ISCIII-SGEFI/ERDF).

Bibliography

Aguiar, Derek, and Sorin Istrail. 2013. "Haplotype Assembly in Polyploid Genomes and Identical by Descent Shared Tracts." *Bioinformatics (Oxford, England)* 29 (13): i352-60. https://doi.org/10.1093/bioinformatics/btt213.

Ahrendt, Steven R., C. Alisha Quandt, Doina Ciobanu, Alicia Clum, Asaf Salamov, Bill Andreopoulos, Jan-Fang Cheng, et al. 2018. "Leveraging Single-Cell Genomics to Expand the Fungal Tree of Life." *Nature Microbiology* 3 (October): 1417–1428. https://doi.org/10.1038/s41564-018-0261-0.

Anderson, Cori A, Samantha Roberts, Huaiying Zhang, Courtney M Kelly, Alexxy Kendall, ChangHwan Lee, John Gerstenberger, Aaron B Koenig, Ruth Kabeche, and Amy S Gladfelter. 2015. "Ploidy Variation in Multinucleate Cells Changes under Stress." *Molecular Biology of the Cell* 26 (6): 1129–40. https://doi.org/10.1091/mbc.E14-09-1375.

Anderson, Matthew Z, Amrita Saha, Abid Haseeb, and Richard J Bennett. 2017. "A

Chromosome 4 Trisomy Contributes to Increased Fluconazole Resistance in a Clinical Isolate of *Candida albicans*.” *Microbiology (Reading, England)* 163 (6): 856–65. https://doi.org/10.1099/mic.0.000478.

Avramova, Marta, Alice Cibrario, Emilien Peltier, Monika Coton, Emmanuel Coton, Joseph Schacherer, Giuseppe Spano, et al. 2018. “*Brettanomyces bruxellensis* Population Survey Reveals a Diploid-Triploid Complex Structured According to Substrate of Isolation and Geographical Distribution.” *Scientific Reports* 8 (1): 1–13. https://doi.org/10.1038/s41598-018-22580-7.

Bankevich, Anton, Sergey Nurk, Dmitry Antipov, Alexey A. Gurevich, Mikhail Dvorkin, Alexander S. Kulikov, Valery M. Lesin, et al. 2012. “SPAdes: A New Genome Assembly Algorithm and Its Applications to Single-Cell Sequencing.” *Journal of Computational Biology* 19 (5): 455–77. https://doi.org/10.1089/cmb.2012.0021.

Benjamini, Yuval, and Terence P. Speed. 2012. “Summarizing and Correcting the GC Content Bias in High-Throughput Sequencing.” *Nucleic Acids Research*. https://doi.org/10.1093/nar/gks001.

Berman, Judith. 2016. “Ploidy Plasticity: A Rapid and Reversible Strategy for Adaptation to Stress.” Edited by Carol Munro. *FEMS Yeast Research* 16 (3): fow020. https://doi.org/10.1093/femsyr/fow020.

Berman, Judith, Noa Blutraich Wertheimer, and Neil Stone. 2016. “Ploidy Dynamics and Evolvability in Fungi.” *Philosophical Transactions of the Royal Society of London B: Biological Sciences* 371 (20150461): 1–11. https://doi.org/10.1098/rstb.2015.0461.

Blanquer, Andrea, and Maria-J. Uriz. 2011. "'Living Together Apart': The Hidden Genetic Diversity of Sponge Populations." *Molecular Biology and Evolution* 28 (9): 2435–38. https://doi.org/10.1093/molbev/msr096.

Bolger, Anthony M, Marc Lohse, and Bjoern Usadel. 2014. "Trimmomatic: A Flexible Trimmer for Illumina Sequence Data." *Bioinformatics (Oxford, England)* 30 (15): 2114–20. https://doi.org/10.1093/bioinformatics/btu170.

Bonizzoni, Paola, Riccardo Dondi, Gunnar W. Klau, Yuri Pirola, Nadia Pisanti, and Simone Zaccaria. 2016. "On the Minimum Error Correction Problem for Haplotype Assembly in Diploid and Polyploid Genomes." *Journal of Computational Biology* 23 (9): 718–36. https://doi.org/10.1089/cmb.2015.0220.

Borneman, Anthony R, Ryan Zeppel, Paul J Chambers, and Chris D Curtin. 2014. "Insights into the Dekkera Bruxellensis Genomic Landscape: Comparative Genomics Reveals Variations in Ploidy and Nutrient Utilisation Potential amongst Wine Isolates." *PLoS Genetics* 10 (2): e1004161. https://doi.org/10.1371/journal.pgen.1004161.

Brudno, Michael, Michael Chapman, Berthold Göttgens, Serafim Batzoglou, Burkhard Morgenstern, S Knowles, JM Bye, DM Beare, and I Dunham. 2003. "Gene Prediction in Eukaryotes with a Generalized Hidden Markov Model That Uses Hints from External Sources." *BMC Bioinformatics* 4 (1): 66. https://doi.org/10.1186/1471-2105-4-66.

Burmester, Anke, Sedighe Karimi, Jana Wetzel, and Johannes Wöstemeyer. 2013. "Complementation of a Stable Met2-1 Mutant of the Zygomycete Absidia Glauca by the Corresponding Wild-Type Allele of the Mycoparasite Parasitella Parasitica, Transferred during Infection." *Microbiology (Reading, England)* 159 (Pt 8): 1639–48. https://doi.org/10.1099/MIC.0.066910-0.

Chibucos, Marcus C., Sameh Soliman, Teclegiorgis Gebremariam, Hongkyu Lee, Sean Daugherty, Joshua Orvis, Amol C. Shetty, et al. 2016. “An Integrated Genomic and Transcriptomic Survey of Mucormycosis-Causing Fungi.” *Nature Communications* 7 (July): 12218. https://doi.org/10.1038/ncomms12218.

Consortium, The Uniprot. 2014. “Activities at the Universal Protein Resource (UniProt).” *Nucleic Acids Research* 42 (Database issue): D191-8. https://doi.org/10.1093/nar/gkt1140.

Corrochano, Luis M, Alan Kuo, Marina Marcet-Houben, Silvia Polaino, Asaf Salamov, José M Villalobos-Escobedo, Jane Grimwood, et al. 2016. “Expansion of Signal Transduction Pathways in Fungi by Extensive Genome Duplication.” *Current Biology* 26 (12): 1577–84. https://doi.org/10.1016/j.cub.2016.04.038.

D.M., Emms, and Kelly S. 2019. “OrthoFinder2: Phylogenetic orthology inference for comparative genomics.” *Genome Biology* **20** (238) 1-14.

Depotter, Jasper Rl, Michael F Seidl, Thomas A Wood, and Bart Phj Thomma. 2016. “Interspecific Hybridization Impacts Host Range and Pathogenicity of Filamentous Microbes.” *Current Opinion in Microbiology* 32: 7–13. https://doi.org/10.1016/j.mib.2016.04.005.

Garbelotto, Matteo, Paolo Gonthier, Rachel Linzer, Giovanni Nicolotti, and William Otrosina. 2004. “A Shift in Nuclear State as the Result of Natural Interspecific Hybridization between Two North American Taxa of the Basidiomycete Complex Heterobasidion.” *Fungal Genetics and Biology : FG & B* 41 (11): 1046–51. https://doi.org/10.1016/j.fgb.2004.08.003.

Gawad, Charles, Winston Koh, and Stephen R. Quake. 2016. “Single-Cell Genome Sequencing:

Current State of the Science.” *Nature Reviews Genetics*. Nature Publishing Group. https://doi.org/10.1038/nrg.2015.16.

Gerdol, Marco, Rebeca Moreira, Fernando Cruz, Jessica Gómez-Garrido, Anna Vlasova, Umberto Rosani, Paola Venier, et al. 2020. “Massive Gene Presence-Absence Variation Shapes an Open Pan-Genome in the Mediterranean Mussel.” *Genome Biology* 21 (1): 275. https://doi.org/10.1186/s13059-020-02180-3.

Gerstein, Aleeza C, and Judith Berman. 2015. “Shift and Adapt: The Costs and Benefits of Karyotype Variations.” *Current Opinion in Microbiology* 26 (August): 130–36. https://doi.org/10.1016/j.mib.2015.06.010.

Gerstein, Aleeza C, Man Shun Fu, Liliane Mukaremera, Zhongming Li, Kate L Ormerod, James A Fraser, Judith Berman, and Kirsten Nielsen. 2015. “Polyploid Titan Cells Produce Haploid and Aneuploid Progeny to Promote Stress Adaptation.” *MBio* 6 (5): 1–14. https://doi.org/10.1128/mBio.01340-15.

Golicz, Agnieszka A., Jacqueline Batley, and David Edwards. 2016. “Towards Plant Pangenomics.” *Plant Biotechnology Journal* 14 (4): 1099–1105. https://doi.org/10.1111/pbi.12499.

Grigoriev, Igor V, Roman Nikitin, Sajeet Haridas, Alan Kuo, Robin Ohm, Robert Otillar, Robert Riley, et al. 2014. “MycoCosm Portal: Gearing up for 1000 Fungal Genomes.” *Nucleic Acids Research* 42: 699–704. https://doi.org/10.1093/nar/gkt1183.

Gurevich, Alexey, Vladislav Saveliev, Nikolay Vyahhi, and Glenn Tesler. 2013. “QUAST: Quality Assessment Tool for Genome Assemblies.” *Bioinformatics* 29 (8): 1072–75. https://doi.org/10.1093/BIOINFORMATICS/BTT086.

Harrison, Benjamin D, Jordan Hashemi, Maayan Bibi, Rebecca Pulver, Danny Bavli, Yaakov Nahmias, Melanie Wellington, Guillermo Sapiro, and Judith Berman. 2014. "A Tetraploid Intermediate Precedes Aneuploid Formation in Yeasts Exposed to Fluconazole." *PLoS Biology* 12 (3): 1–18. https://doi.org/10.1371/journal.pbio.1001815.

Hirsch, Candice N., and C. Robin Buell. 2013. "Tapping the Promise of Genomics in Species with Complex, Nonmodel Genomes." *Annual Review of Plant Biology* 64 (1): 89–110. https://doi.org/10.1146/annurev-arplant-050312-120237.

Horn, Fabian, Zerrin Üzüm, Nadine Möbius, Reinhard Guthke, Jörg Linde, and Christian Hertweck. 2015. "Draft Genome Sequences of Symbiotic and Nonsymbiotic Rhizopus Microsporus Strains CBS 344.29 and ATCC 62417." *Genome Announcements* 3 (1). https://doi.org/10.1128/GENOMEA.01370-14.

Huang, Lei, Fei Ma, Alec Chapman, Sijia Lu, and Xiaoliang Sunney Xie. 2015. "Single-Cell Whole-Genome Amplification and Sequencing: Methodology and Applications." *Annual Review of Genomics and Human Genetics* 16 (August): 79–102. https://doi.org/10.1146/annurev-genom-090413-025352.

James, Stephen a., Christopher J. Bond, Malcolm Stratford, and Ian N. Roberts. 2005. "Molecular Evidence for the Existence of Natural Hybrids in the Genus *Zygosaccharomyces*." *FEMS Yeast Research* 5 (8): 747–55. https://doi.org/10.1016/j.femsyr.2005.02.004.

James, Timothy Y., Jan Stenlid, Åke Olson, and Hanna Johannesson. 2008. "Evolutionary Significance of Imbalanced Nuclear Ratios within Heterokaryons of the Basidiomycete Fungus *Heterobasidion parviporum*." *Evolution* 62 (9): 2279–96.

https://doi.org/10.1111/j.1558-5646.2008.00462.x.

Kajitani, Rei, Kouta Toshimoto, Hideki Noguchi, Atsushi Toyoda, Yoshitoshi Ogura, Miki Okuno, Mitsuru Yabana, et al. 2014. “Efficient de Novo Assembly of Highly Heterozygous Genomes from Whole-Genome Shotgun Short Reads.” *Genome Research* 24 (8): 1384–95. https://doi.org/10.1101/gr.170720.113.

Kravets, Anatoliy, Feng Yang, Gabor Bethlendy, Fred Sherman, and Elena Rustchenko. 2014. “Adaptation of *Candida albicans* to Growth on Sorbose via Monosomy of Chromosome 5 Accompanied by Duplication of Another Chromosome Carrying a Gene Responsible for Sorbose Utilization.” *FEMS Yeast Research* 14 (5): 708–13. https://doi.org/10.1111/1567-1364.12155.Adaptation.

Kumar, Sujai, Martin Jones, Georgios Koutsovoulos, Michael Clarke, and Mark Blaxter. 2013. “Blobology: Exploring Raw Genome Data for Contaminants, Symbionts and Parasites Using Taxon-Annotated GC-Coverage Plots.” *Frontiers in Genetics* 4 (November): 237. https://doi.org/10.3389/fgene.2013.00237.

Kumaran, Rajaraman, Shi Yow Yang, and Jun Yi Leu. 2013. “Characterization of Chromosome Stability in Diploid, Polyploid and Hybrid Yeast Cells.” *PLoS ONE* 8 (7). https://doi.org/10.1371/journal.pone.0068094.

Laetsch, Dominik R., and Mark L. Blaxter. 2017. “BlobTools: Interrogation of Genome Assemblies.” *F1000Research* 6: 1287. https://doi.org/10.12688/f1000research.12232.1.

Lenassi, Metka, Cene Gostinčar, Shaun Jackman, Martina Turk, Ivan Sadowski, Corey Nislow, Steven Jones, Inanc Birol, Nina Gunde Cimerman, and Ana Plemenitaš. 2013. “Whole Genome Duplication and Enrichment of Metal Cation Transporters Revealed by De Novo

Genome Sequencing of Extremely Halotolerant Black Yeast *Hortaea werneckii*.” Edited by Jason E. Stajich. *PLoS ONE* 8 (8): 1–18. https://doi.org/10.1371/journal.pone.0071328.

Li, Heng. 2013. “Aligning Sequence Reads, Clone Sequences and Assembly Contigs with BWA-MEM.” *ArXiv Preprint ArXiv*, 1–3.

Li, Wenjun, Anna Floyd Averette, Marie Desnos-Ollivier, Min Ni, Françoise Dromer, and Joseph Heitman. 2012. “Genetic Diversity and Genomic Plasticity of *Cryptococcus neoformans* AD Hybrid Strains.” *G3: Genes, Genomes, Genetics* 2 (1): 83–97. https://doi.org/10.1534/g3.111.001255.

Louis, V. L., L. Despons, A. Friedrich, T. Martin, P. Durrens, S. Casaregola, C. Neuveglise, et al. 2012. “*Pichia sorbitophila*, an Interspecies Yeast Hybrid, Reveals Early Steps of Genome Resolution After Polyploidization.” *G3: Genes, Genomes, Genetics* 2 (2): 299–311. https://doi.org/10.1534/g3.111.000745.

Lu, Jennifer, and Steven L. Salzberg. 2018. “Removing Contaminants from Databases of Draft Genomes.” Edited by Fengzhu Sun. *PLOS Computational Biology* 14 (6): e1006277. https://doi.org/10.1371/journal.pcbi.1006277.

Luo, Ruibang, Binghang Liu, Yinlong Xie, Zhenyu Li, Weihua Huang, Jianying Yuan, Guangzhu He, et al. 2012. “SOAPdenovo2: An Empirically Improved Memory-Efficient Short-Read de Novo Assembler.” *GigaScience* 1 (1): 18. https://doi.org/10.1186/2047-217X-1-18.

Ma, Li-Jun, Ashraf S. Ibrahim, Christopher Skory, Manfred G. Grabherr, Gertraud Burger, Margi Butler, Marek Elias, et al. 2009. “Genomic Analysis of the Basal Lineage Fungus *Rhizopus oryzae* Reveals a Whole-Genome Duplication.” . *PLoS Genetics* 5 (7): 1–11.

https://doi.org/10.1371/journal.pgen.1000549.

Maheshwari, Ramesh. 2005. “Nuclear Behavior in Fungal Hyphae.” *FEMS Microbiology Letters* 249: 7–14. https://doi.org/10.1016/j.femsle.2005.06.031.

Mannaert, An, Tim Downing, Hideo Imamura, and Jean Claude Dujardin. 2012. “Adaptive Mechanisms in Pathogens: Universal Aneuploidy in Leishmania.” *Trends in Parasitology*. https://doi.org/10.1016/j.pt.2012.06.003.

Mapleson, Daniel, Gonzalo Garcia Accinelli, George Kettleborough, Jonathan Wright, and Bernardo J Clavijo. 2016. “KAT: A K-Mer Analysis Toolkit to Quality Control NGS Datasets and Genome Assemblies.” *Bioinformatics* 33 (4): 574–76. https://doi.org/10.1093/bioinformatics/btw663.

Margarido, Gabriel R. A., David Heckerman, EW Myers, GG Sutton, AL Delcher, IM Dew, DP Fasulo, et al. 2015. “ConPADE: Genome Assembly Ploidy Estimation from Next-Generation Sequencing Data.”. *PLOS Computational Biology* 11 (4): e1004229. https://doi.org/10.1371/journal.pcbi.1004229.

McCarthy, Charley G. P., and David A. Fitzpatrick. 2019. “Pan-Genome Analyses of Model Fungal Species.” *Microbial Genomics* 5 (2): 1–23. https://doi.org/10.1099/mgen.0.000243.

McKenna, Aaron, Matthew Hanna, Eric Banks, Andrey Sivachenko, Kristian Cibulskis, Andrew Kernytsky, Kiran Garimella, et al. 2010. “The Genome Analysis Toolkit: A MapReduce Framework for Analyzing next-Generation DNA Sequencing Data.” *Genome Research* 20 (9): 1297–1303. https://doi.org/10.1101/gr.107524.110.

Mehrabi, Rahim, Amir Mirzadi Gohari, and Gert H.J. Kema. 2017. “Karyotype Variability in Plant-Pathogenic Fungi.” *Annual Review of Phytopathology* 55 (1): 483–503.

https://doi.org/10.1146/annurev-phyto-080615-095928.

Mixão, Verónica, and Toni Gabaldón. 2018. “Yeast Interspecies Hybrids Hybridization and Emergence of Virulence in Opportunistic Human Yeast Pathogens.” *Yeast* 35: 5–20. https://doi.org/10.1002/yea.3242.

Morrow, Carl a., and James a. Fraser. 2013. “Ploidy Variation as an Adaptive Mechanism in Human Pathogenic Fungi.” *Seminars in Cell and Developmental Biology* 24 (4): 339–46. https://doi.org/10.1016/j.semcdb.2013.01.008.

Naranjo-Ortiz, M.A., and T. Gabaldón. 2019. “Fungal Evolution: Diversity, Taxonomy and Phylogeny of the Fungi.” *Biological Reviews* 94 (6). https://doi.org/10.1111/brv.12550.

Naranjo- Ortiz, Miguel A., and Toni Gabaldón. 2020. “Fungal Evolution: Cellular, Genomic and Metabolic Complexity.” *Biological Reviews*, April, brv.12605. https://doi.org/10.1111/brv.12605.

Peter, Jackson, Matteo De Chiara, Anne Friedrich, Jia-Xing Yue, David Pflieger, Anders Bergström, Anastasie Sigwalt, et al. 2018. “Genome Evolution across 1,011 Saccharomyces Cerevisiae Isolates.” *Nature* 556 (7701): 339–44. https://doi.org/10.1038/s41586-018-0030-5.

Pryszcz, Leszek P, and Toni Gabaldón. 2016. “Redundans : An Assembly Pipeline for Highly Heterozygous Genomes.” *Nucleic Acids Research* 8 (44): 1–16. https://doi.org/10.1093/nar/gkw294.

Ross, Michael G., Carsten Russ, Maura Costello, Andrew Hollinger, Niall J. Lennon, Ryan Hegarty, Chad Nusbaum, and David B. Jaffe. 2013. “Characterizing and Measuring Bias in Sequence Data.” *Genome Biology*. https://doi.org/10.1186/gb-2013-14-5-r51.

Safonova, Yana, Anton Bankevich, and Pavel A Pevzner. 2015. “DipSPAdes: Assembler for Highly Polymorphic Diploid Genomes.” *Journal of Computational Biology : A Journal of Computational Molecular Cell Biology* 22 (6): 528–45. https://doi.org/10.1089/cmb.2014.0153.

Schmieder, Robert, and Robert Edwards. 2011. “Fast Identification and Removal of Sequence Contamination from Genomic and Metagenomic Datasets.” *PLoS ONE* 6 (3): e17288. https://doi.org/10.1371/journal.pone.0017288.

Schoenfelder, Kevin P, and Donald T Fox. 2015. “The Expanding Implications of Polyploidy.” *The Journal of Cell Biology* 209 (4): 485–91. https://doi.org/10.1083/jcb.201502016.

Scott, Amber L, Phillip A Richmond, Robin D Dowell, and Anna M Selmecki. 2017. “The Influence of Polyploidy on the Evolution of Yeast Grown in a Sub-Optimal Carbon Source.” *Molecular Biology and Evolution* 34 (10): 2690–2703. https://doi.org/10.1093/molbev/msx205.

Scott, Derrick, and Bert Ely. 2014. “Comparison of Genome Sequencing Technology and Assembly Methods for the Analysis of a GC-Rich Bacterial Genome.” *Current Microbiology* 70 (3): 338–44. https://doi.org/10.1007/s00284-014-0721-6.

Sibbald, Shannon J., Laura Eme, John M. Archibald, and Andrew J. Roger. 2020. “Lateral Gene Transfer Mechanisms and Pan-Genomes in Eukaryotes.” *Trends in Parasitology*, August. https://doi.org/10.1016/j.pt.2020.07.014.

Simâo, Felipe A., Robert M. Waterhouse, Panagiotis Ioannidis, Evgenia V. Kriventseva, and Evgeny M. Zdobnov. 2015. "BUSCO: Assessing Genome Assembly and Annotation Completeness with Single-Copy Orthologs." *Bioinformatics* 31 (19): 3210–12. https://doi.org/10.1093/bioinformatics/btv351.

Simpson, Jared T., and Mihai Pop. 2015. "The Theory and Practice of Genome Sequence Assembly." *Annual Review of Genomics and Human Genetics* 16 (1): 153–72. https://doi.org/10.1146/annurev-genom-090314-050032.

Sinha, Sunita, Stephane Flibotte, Mauricio Niera, Sean Formby, Ana Plemenitaš, Nina Gunde Cimerman, Metka Lenassi, Cene Gostinčar, Jason E. Stajich, and Corey Nislow. 2017. "Insight into the Recent Genome Duplication of the Halophilic Yeast *Hortaea werneckii*: Combining an Improved Genome with Gene Expression and Chromatin Structure." *G3: Genes, Genomes, Genetics* 7 (7): 2015–22. http://www.g3journal.org/content/early/2017/05/12/g3.117.040691.

Stephen F. Altschul, Warren Gish, Webb Miller, Eugene W. Myers and David J. Lipman. 1990. "BLAST." Journal of Molecular Biology. 1990. **215**, 403-410

Strom, Noah B, and Kathryn E Bushley. 2016. "Two Genomes Are Better than One: History, Genetics, and Biotechnological Applications of Fungal Heterokaryons." *Fungal Biology and Biotechnology* 3 (4): 1–14. https://doi.org/10.1186/s40694-016-0022-x.

Strope, Pooja K, Daniel A Skelly, Stanislav G Kozmin, Gayathri Mahadevan, Eric A Stone, Paul M Magwene, Fred S Dietrich, and John H McCusker. 2015. "The 100-Genomes Strains, an *S. cerevisiae* Resource That Illuminates Its Natural Phenotypic and Genotypic Variation and Emergence as an Opportunistic Pathogen." *Genome Research* 25 (5): 762–74.

https://doi.org/10.1101/gr.185538.114.

Todd, Robert T, Anja Forche, and Anna Selmecki. 2017. "Ploidy Variation in Fungi: Polyploidy, Aneuploidy, and Genome Evolution." *Microbiology Spectrum*, **5** (4), 599–618.. https://doi.org/10.1128/microbiolspec.FUNK-0051-2016.

Torres, Eduardo M, Bret R Williams, and Angelika Amon. 2008. "Aneuploidy: Cells Losing Their Balance." *Genetics* 179 (2): 737–46. https://doi.org/10.1534/genetics.108.090878.

Trivedi, Urmi H., Timothée Cézard, Stephen Bridgett, Anna Montazam, Jenna Nichols, Mark Blaxter, and Karim Gharbi. 2014. "Quality Control of Next-Generation Sequencing Data without a Reference." *Frontiers in Genetics* 5 (MAY): 111. https://doi.org/10.3389/fgene.2014.00111.

Tůmová, Pavla, Magdalena Uzlíková, Tomáš Jurczyk, and Eva Nohýnková. 2016. "Constitutive Aneuploidy and Genomic Instability in the Single-Celled Eukaryote *Giardia intestinalis*." *MicrobiologyOpen* 5 (4): 560–74. https://doi.org/10.1002/mbo3.351.

Wajid, Bilal, and Erchin Serpedin. 2012. "Review of General Algorithmic Features for Genome Assemblers for Next Generation Sequencers." *Genomics, Proteomics & Bioinformatics* 10: 58–73. https://doi.org/10.1016/j.gpb.2012.05.006.

Wajid, Bilal, Muhammad U Sohail, Ali R Ekti, and Erchin Serpedin. 2016. "The A, C, G, and T of Genome Assembly." *BioMed Research International* 2016: 6329217. https://doi.org/10.1155/2016/6329217.

Walther, Andrea, Ana Hesselbart, and Jürgen Wendland. 2014. "Genome Sequence of Saccharomyces Carlsbergensis, the World's First Pure Culture Lager Yeast." *G3: Genes, Genomes, Genetics* 4 (5): 1–11. https://doi.org/10.1534/g3.113.010090.

Weiß , Clemens L., Marina Pais, Liliana M. Cano, Sophien Kamoun, and Hernán A. Burbano. 2018. “nQuire: A Statistical Framework for Ploidy Estimation Using next Generation Sequencing.” *BMC Bioinformatics* 19 (1): 122. https://doi.org/10.1186/s12859-018-2128-z.

Wilkening, Stefan, Manu M. Tekkedil, Gen Lin, Emilie S. Fritsch, Wu Wei, Julien Gagneur, David W. Lazinski, Andrew Camilli, and Lars M. Steinmetz. 2013. “Genotyping 1000 Yeast Strains by Next-Generation Sequencing.” *BMC Genomics* 14 (1). https://doi.org/10.1186/1471-2164-14-90.

Zhu, Yuan O., Gavin Sherlock, and Dmitri A. Petrov. 2016. “Whole Genome Analysis of 132 Clinical Saccharomyces Cerevisiae Strains Reveals Extensive Ploidy Variation.” *G3: Genes, Genomes, Genetics* 6 (8). https://doi.org/10.1534/g3.116.029397.

Zörgö, Enikö, Karolina Chwialkowska, Arne B. Gjuvsland, Elena Garré, Per Sunnerhagen, Gianni Liti, Anders Blomberg, Stig W. Omholt, and Jonas Warringer. 2013. “Ancient Evolutionary Trade-Offs between Yeast Ploidy States.” *PLoS Genetics* 9 (3). https://doi.org/10.1371/JOURNAL.PGEN.1003388.

Figure 1: Factors that difficult genome assembly

Ploidy and aneuploidy increase the number of possible states per site. Extreme GC% composition affects the information that different *k*-mers have, and extreme deviations are relatively common in extremophilic organisms. Transposable elements and other forms of repetitive elements increase genome size, affect GC% locally and reduce sequence complexity. Hybridization, heterokaryosis and chimerism introduce two genotypic signals that might be quite divergent, which increases heterozygosity. Finally, contamination introduces undesired sequences with uneven composition, heterozygosity and stoichiometry.

Figure 2: Karyon pipeline

Schematic representation of the steps and program used by Karyon. Red circles represent possible user inputs. Blue boxes represent software used for each step. Orange hexagons represent files generated by the software. Red arrows indicate input to a program, blue arrows represent output of a program. Thicker red arrows represent the standard pipeline, while thinner red arrows represent the different options the user can select to skip some of the steps. These options appear next to the arrow.

Figure 3. Summary of karyonplots

A) Scaffold length plot. **B)** Scaffold length versus coverage plot. In the example, scaffolds form two populations with different coverage, which suggests aneuploidy behavior. **C)** Variation versus coverage plot. In the example the genome forms a clear population with low SNP density and approximately 30x of coverage; and a second more diffuse population with higher SNP density and approximately 60x coverage. This behavior suggests a mix of haploid and diploid regions. **D)** Fair coin analysis. The red line represents a simulated distribution assuming perfect 50% distribution of reference and alternative SNPs. Each blue line represents the empirical distribution of reference versus alternative SNPs per scaffold, which in this case all follow a diploid distribution. **E)** Per scaffold nQuire plot. The plot represents nQuire generated normalized values across sliding windows for a single scaffold. The vast majority of windows have high diploid score, which suggests that this particular scaffold is diploid.

Figure 4. Analysis of *Rhizopus microsporus* ATCC62417

A) Variation versus coverage plot reveals the existence of a highly variable portion of the genome

that presents variable heterozygosity levels. B) BlobTools analyses suggest that the genome presents a considerable portion of contaminating sequences. Coverage of the sequences assigned to bacteria is very low when the analyses are performed with other libraries (Data not shown), which proves that the conflicting signal observed in this sample has its origin in a contaminated sequencing library. Results for *R. microsporus* CBS344.5 and var. *rhizopodiformus* B7455 show similar patterns of contamination (data not shown).

Figure 5. Analysis of *Rhizopus microsporus* B9738

A) KAT *k*-mer plot shows very low genome compaction (black area), suggestive of a haploid genome. **B)** Variation versus coverage plot reveals a single main behavior for the genome with regards of its SNP density and coverage. **C)** BlobTools analysis shows no sign of widespread contamination that might be inflating the genome.

Figure 6. Phylogenetic tree of *Rhizopus microsporus* B9738

Phylogenetic tree inferred from OrthoFinder. The *Rhizopus microsporus* species complex is marked in blue. The problematic strain, B9738, is marked in yellow.

Figure 7. Analysis of *Mucor racemosus* B9645

A) KAT *k*-mer plot shows two peaks of coverage considerably affected by genome reduction (black area), suggestive of a highly heterozygous diploid genome. **B)** Variation versus coverage plot reveals a bimodal behaviour for the genome with regards of its coverage, but both peaks appear with very low SNP density. **C)** BlobTools analysis shows no sign of widespread contamination that might be inflating the genome.

Figure 8. Analysis of *Lichtheimia ramosa* B5399.

A) KAT *k*-mer plot shows one peak with considerable genome compaction (black area) suggestive of a diploid genome. **B)** Variation versus coverage plot reveals a unimodal behaviour for the genome with regards of its coverage, presenting a widespread heterozygosity of approximately 3% (maximum density around 30 SNP/Kbp). **C)** Alternative allele frequency shows that all scaffolds present a behaviour very similar to the ideal diploid. **D)** Scaffold length plot shows that, except for a group of very low coverage scaffolds, all the genome presents a uniform coverage.

Table 1:

NCBI Assembly statistics for the analyzed strains. Strains with darker background possessed some property that was affecting assembly quality and was diagnosed using Karyon. Fragmentation in all remaining strains is attributed to low sequencing depth.

Species	NCBI genome size (Mbp)	NCBI number of scaffolds	GeneBank Accesion	Genome size after Karyon (Mbp)	Number of scaffolds after Karyon	Diagnosis	Reference
Rhizopus microsporus ATCC 62417	49.6	1386	GCA_900000135.1	40.1	5521	Contamination	(Horn et al. 2015)
Rhizopus microsporus CBS_344.29	49.2	1554	GCA_000825725.1	32.1	3037	Contaminatioon	(Horn et al. 2015)
Rhizopus microsporus B9738	75.1	5266	GCA_000697275.1	71.6	12789	Misidentification	(Chibucos et al. 2016)
Rhizopus microsporus var. *rhizopodiformus* B7455	48.7	4658	GCA_000738565.1	21.8	2176	Contamination	(Chibucos et al. 2016)
Rhizopus delemar Type I NRRL 21789	42.0	3921	GCA_000697155.1	33.4	4824	Unknown	(Chibucos et al. 2016)
Rhizopus delemar Type II NRRL 21446	38.9	1156	GCA_000738605.1	33.7	5071	Unknown	(Chibucos et al. 2016)
Rhizopus delemar Type II NRRL 21447	38.7	1177	GCA_000738595.1	28.9	6683	Unknown	(Chibucos et al. 2016)
Rhizopus delemar Type II NRRL 21477	40.8	1808	GCA_000738585.1	None	None	Unknown	(Chibucos et al. 2016)
Rhizopus oryzae 99-892	39.1	1168	GCA_000697725.1	29.6	1875	Low GC% (Below 35%)	(Chibucos et al. 2016)
Rhizopus oryzae HUMC02	40.3	2313	GCA_000697605.1	None	None	Unknown	(Chibucos et al. 2016)
Rhizopus oryzae B7407	43.3	4683	GCA_000696915.1	34.7	3720	Low GC% (Below	(Chibucos et al. 2016)

						35%)	
Rhizopus oryzae type I NRRL 13440	43.4	5022	GCA_000697075.1	None	None	Unknown	(Chibucos et al. 2016)
Rhizopus oryzae type I NRRL 18148	47.5	14653	GCA_000697095.1	None	None	Unknown	(Chibucos et al. 2016)
Rhizopus oryzae type I NRRL 21396	42.8	4445	GCA_000697115.1	34.2	4115	Unknown	(Chibucos et al. 2016)
Rhizopus oryzae 99-133	41.5	4317	GCA_000697135.1	27.2	1332	Unknown	(Chibucos et al. 2016)
Rhizopus oryzae 97-1192	42.9	4566	GCA_000697195.1	None	None	Unknown	(Chibucos et al. 2016)
Rhizopus stolonifer B9770	38	5567	GCA_000697035.1	30.1	6406	Unknown	(Chibucos et al. 2016)
Mucor circinelloides B8987	36.7	2210	GCA_000696935.1	29.9	4864	Unknown	(Chibucos et al. 2016)
Mucor indicus B7402	39.8	3117	GCA_000697295.1	32.1	691	Unknown	(Chibucos et al. 2016)
Mucor racemosus B9645	65.5	6360	GCA_000697255.1	46.0	4444	Hemidiploid, Low GC% (Below 35%)	(Chibucos et al. 2016)
Mucor velutinosus B5328	35.9	2411	GCA_000696895.1	28.2	2743	Unknown	(Chibucos et al. 2016)
Lichtheimia corymbifera 008-049	36.6	1629	GCA_000697175.1	42.8	3575	Unknown	(Chibucos et al. 2016)
Lichtheimia corymbifera B2541	36.6	1176	GCA_000697475.1	13.2	3575	Unknown	(Chibucos et al. 2016)
Lichtheimia ramosa B5399	45.6	3968	GCA_000738555.1	26.6	861	Aneuploid, hybrid	(Chibucos et al. 2016)
Saksenaea oblongisporus B3353	40.8	1702	GCA_000697495.1	29.7	622	Unknown	(Chibucos et al. 2016)
Saksenaea vasiformis B4078	42.5	2417	GCA_000697055.1	32.7	1506	Unknown	(Chibucos et al. 2016)
Cokeromyces recurvatus B5483	29.3	2637	GCA_000697235.1	26.6	5213	Low GC% (Below 35%)	(Chibucos et al. 2016)
Syncephalastrum monosporum B8922	29.6	1284	GCA_000697355.1	24.1	5271	Unknown	(Chibucos et al. 2016)
Syncephalastrum racemosum B6101	29.6	1035	GCA_000696955.1	23.3	311	Unknown	(Chibucos et al. 2016)
Cunninghamella elegans B9769	31.7	1380	GCA_000697015.1	30.8	5465	Low GC% (Below 35%)	(Chibucos et al. 2016)
Apophysomyces elegans B7760	38.5	1528	GCA_000696995.1	29.3	1293	Unknown	(Chibucos et al. 2016)
Apophysomyces trapeziformis B9324	35.8	1400	GCA_000696975.1	30.1	898	Unknown	(Chibucos et al. 2016)
Thermomucor indicae-seudaticae HACC 243	29.6	1958	GCA_000787465.1	25.7	4118	Unknown	Busk et al, unpublished. Genome submitted in 2014.
Parasitella parasitica CBS 412.66 isolate NGI315 ade-mutant	44.9	15637	GCA_000938895.1	23.5	3295	Unknown	(Burmester et al. 2013)

Figure 1

Click here to access/download;Figure;Fig1.png

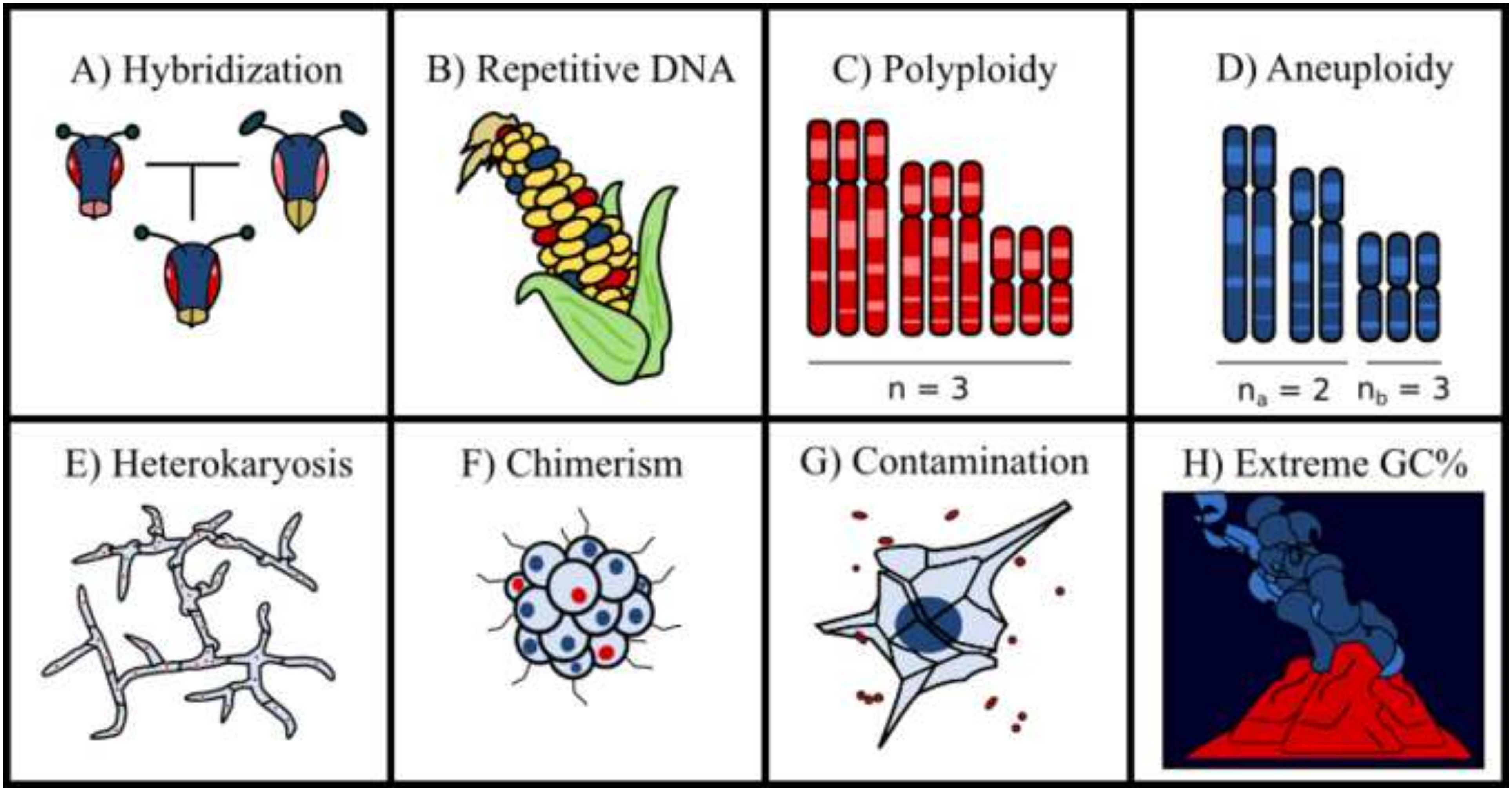

Figure 3

Click here to access/download;Figure;Fig3.png

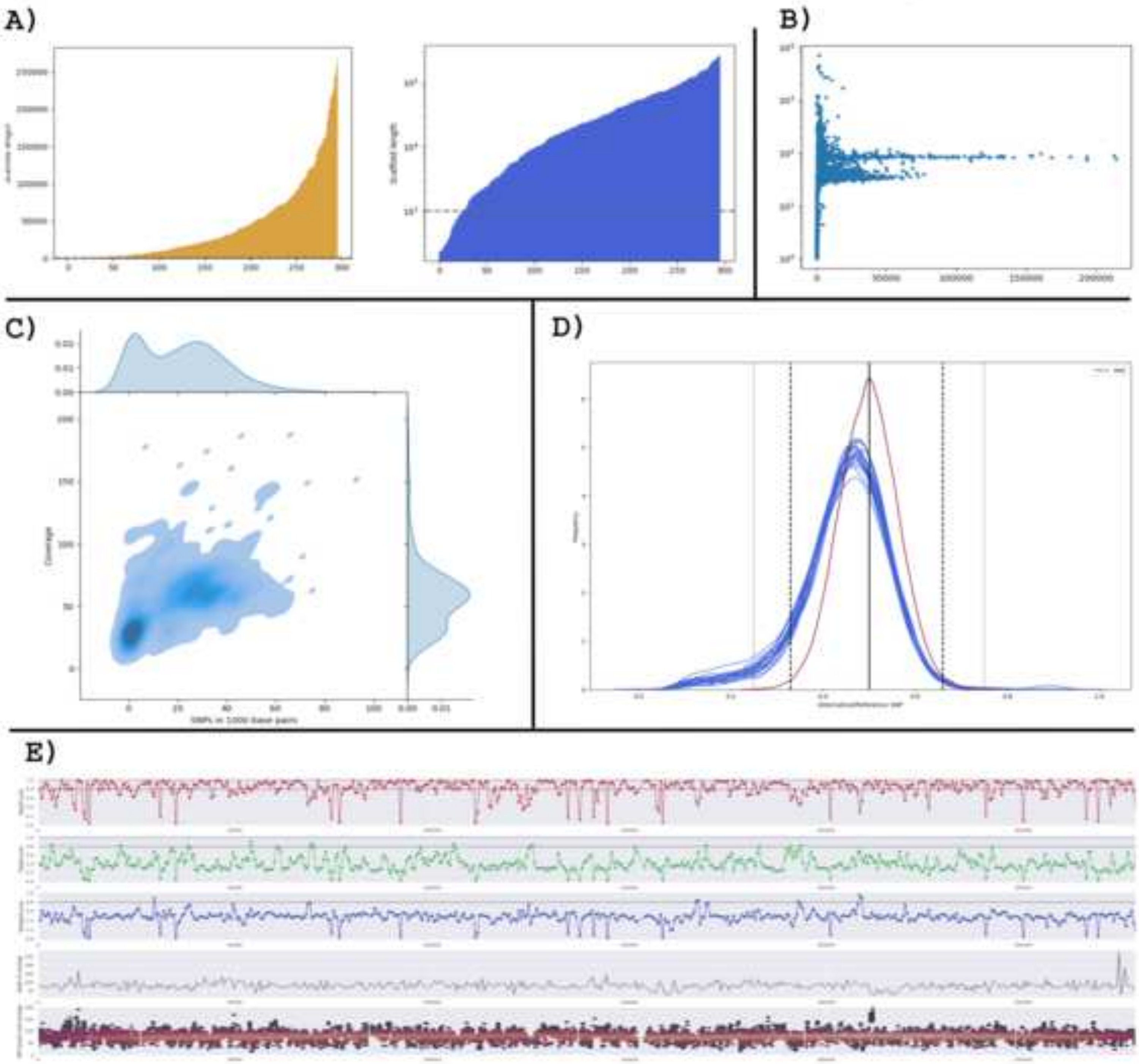

Figure 4

Click here to access/download;Figure;Fig4.png

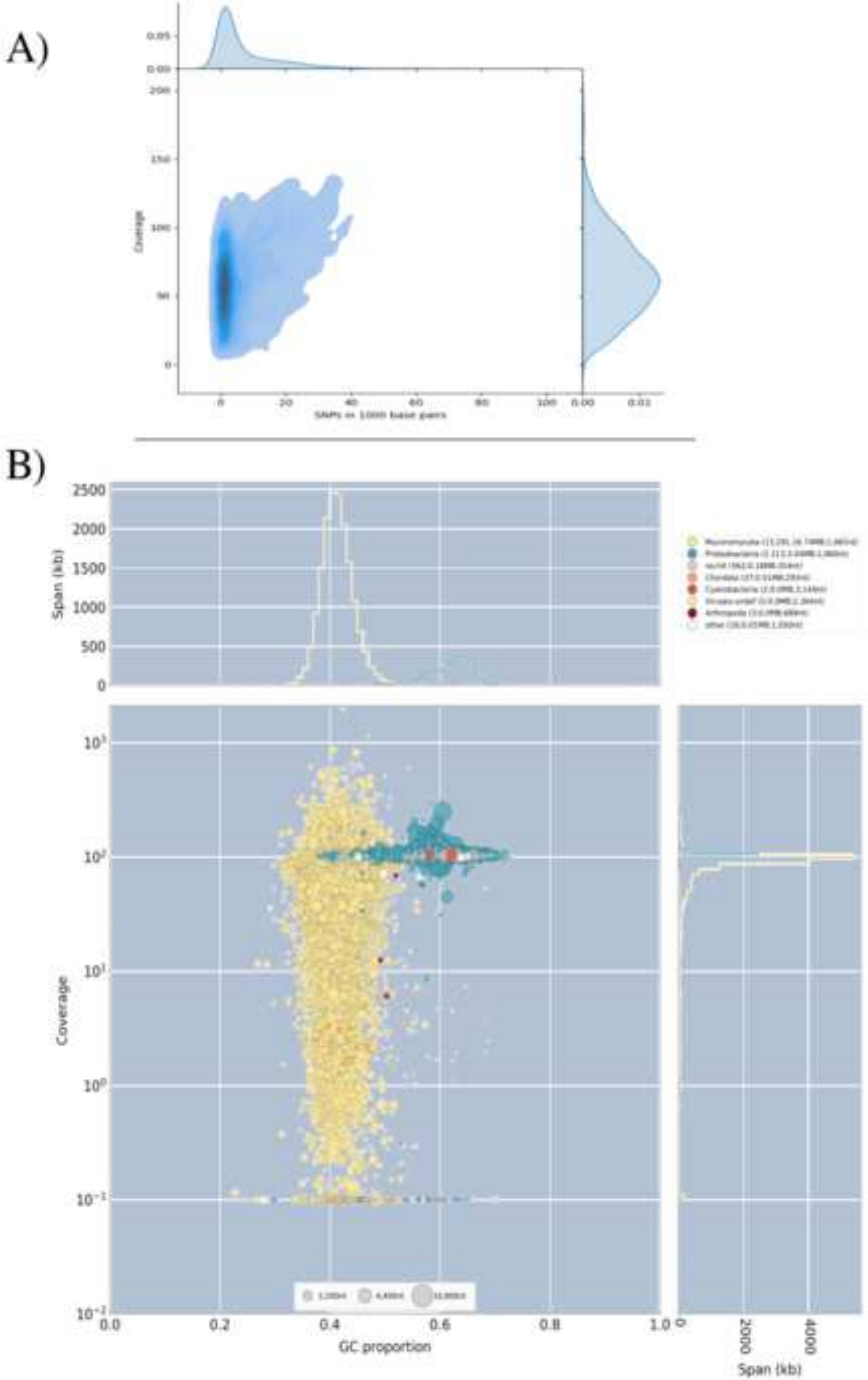

Figure 5

Click here to access/download;Figure;Fig5.png

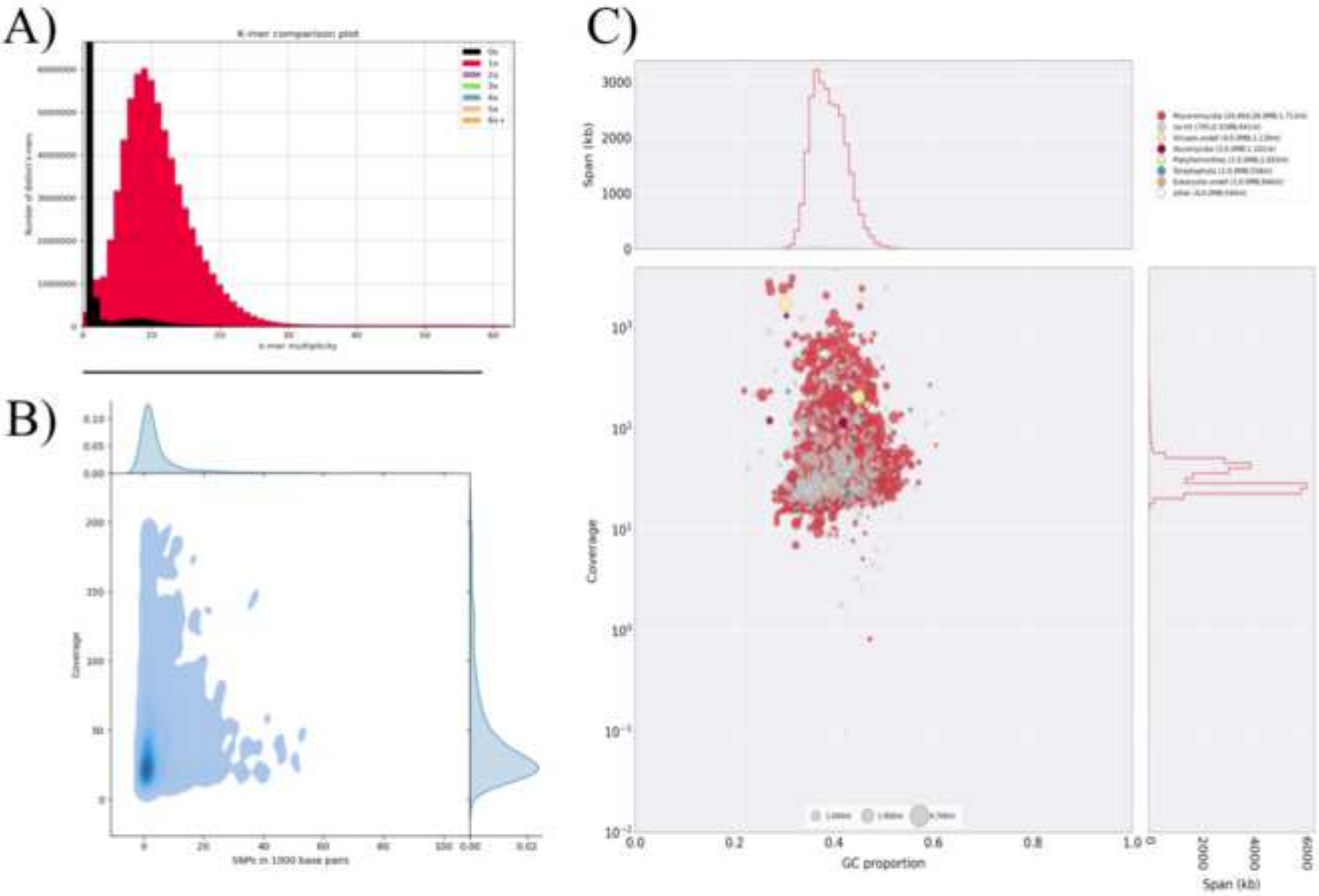

Figure 7

Click here to access/download;Figure;Fig7.png

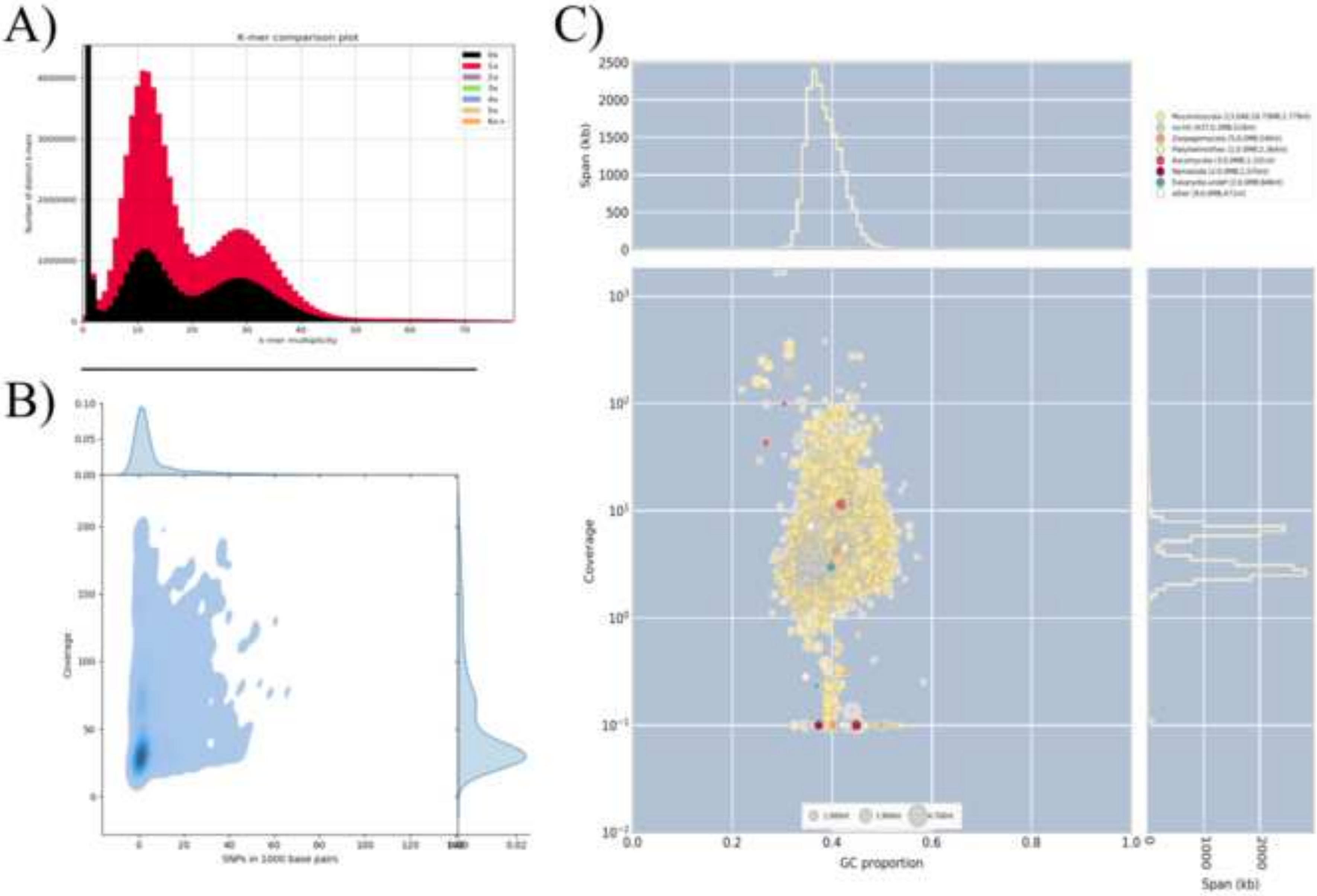

Figure 6

Click here to access/download;Figure;Fig6.png

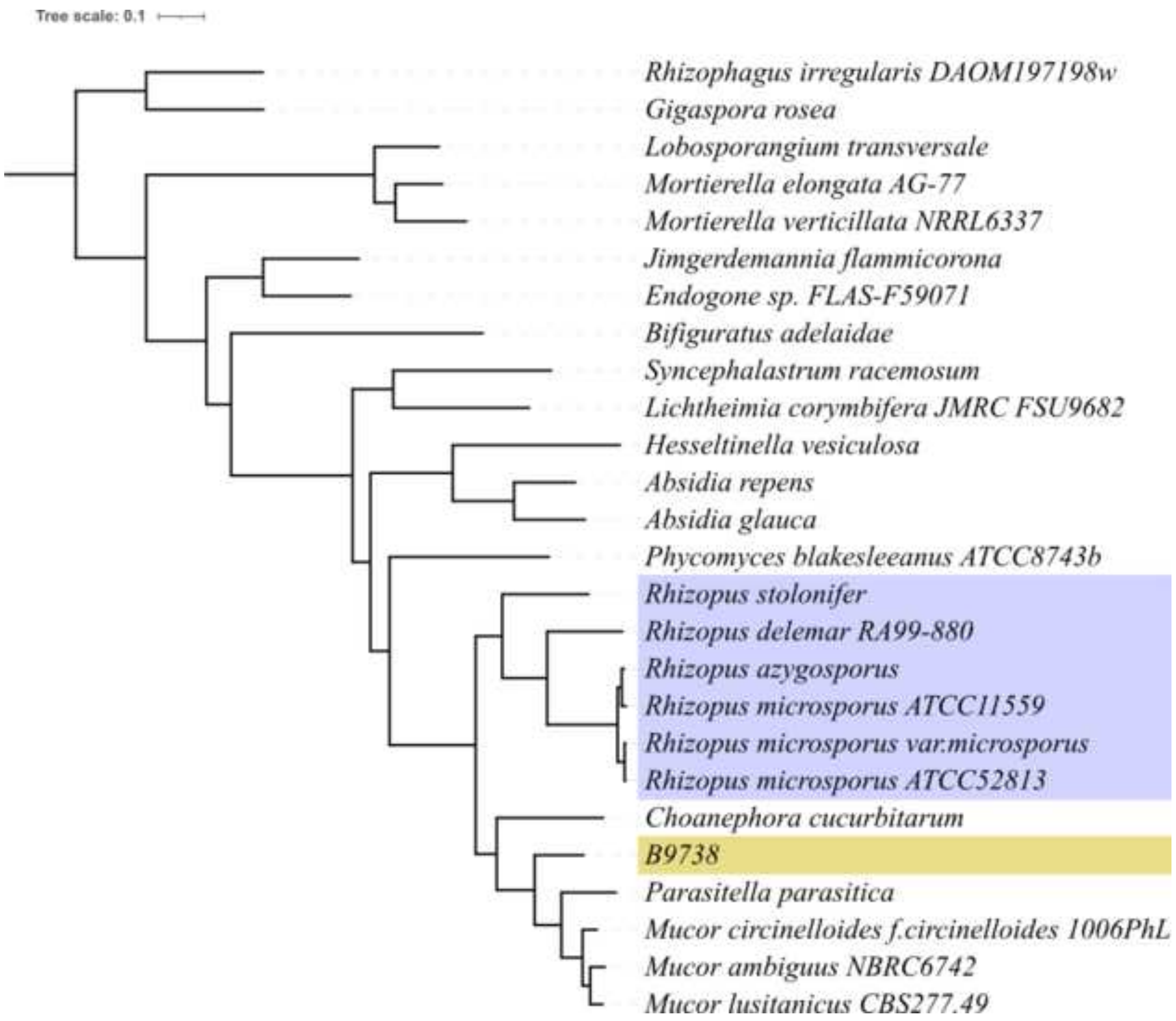

Figure 8

Click here to access/download;Figure;Fig8.png

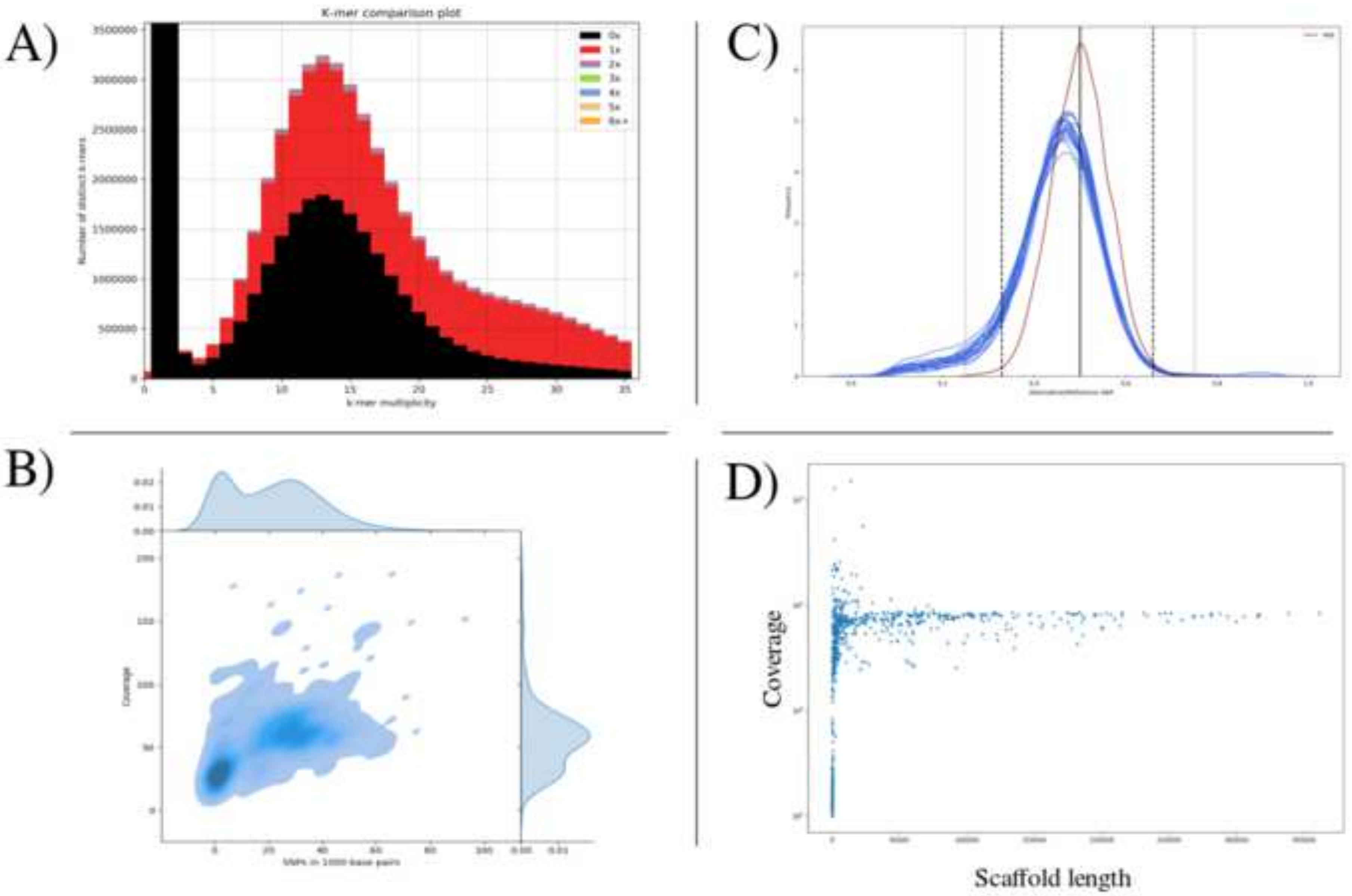

Supplement: giac088_GIGA-D-21-00155_Revision_3 [file giac088_giga-d-21-00155_revision_3.pdf]
