# Supplementary material for: Karyon: a computational framework for the diagnosis of hybrids, aneuploids, and other nonstandard architectures in genome assemblies
Source: Gigascience. 2022 Oct 7;11:giac088. doi: 10.1093/gigascience/giac088 (PMC9540331; doi:10.1093/gigascience/giac088)
Supplement: giac088_Response_to_Reviewer_Comments_Original_Submission [file giac088_response_to_reviewer_comments_original_submission.pdf]

Author's Response To Reviewer Comments

Close

Dear Editor,

Thank you so much for handling our manuscript. I would like to apologize for the delay in preparing the resubmission. The first three authors have already left the laboratory and it has been difficult to organize the requested analysis. As you will see, we have addressed all reviewers comments, which has resulted in significant improvements of the text and manuals and in the inclusion of a new figure.

Below I copy the point-by-point response to each of the reviewers comments

Reviewer reports:

Reviewer #1: In this work, Naranjo-Ortiz et al. presented a software pipeline that is capable of de novo genome assembly, variant calling, and generating diagnostic plots. Applying this software to 35 publically available, highly fragmented fugal genome assemblies revealed prevalent inconsistencies between the sequencing data and the assembly.

I really appreciate the authors' effort to make their software, Karyon, easy to use by providing multiple ways to install and a detailed software manual. I especially like the detailed explanation of how to use the diagnostic plots to infer the "nonstandard genome architectures". The manuscript is clearly written and very easy to follow. I have the following general comments:

Response: We thank the reviewer’s nice comments on our work.

1. It wasn't clear to me the relationships between the raw sequencing data and the assembly -- were they belong to the same isolate? If so, then the inconsistencies may reflect assembly errors in the fungal genome assembly. Have the authors rule our this possibility? The fact that these genomes are highly fragmented suggests they likely contain many errors. If they were from different isolates, then I agree with the authors that the diagnostic plots could be examined carefully to detect structural variations. For that, have the authors used any alternative method to validate at least some of their findings? To establish the validity of their approach, it would be more convincing to obtain the same findings using independent approaches, including experimental ones.

Response. We indeed compare raw sequencing data and assemblies from the same isolate, and we indeed expect that the inconsistencies reflect errors, some of which are triggered by underlying biological factors. These factors are the focus of the analysis. Karyon is not meant to detect structural variations across isolates, but to discover underlying non-standard genomic features (polyploidy, chimerisms, heterozygosity, etc) in sequencing datasets. The pipeline explores properties of the raw data and also creates a de novo assembly, which we are comparing against the existing assembly available at NCBI. Our working hypothesis is that biological properties of these genomes interfere with commonly used genome assembly algorithms. Our pipeline includes some steps to try to minimize the effect of some common biological limiting factors (mainly high heterozygosis), and then runs a series of analyses that try to diagnose the problem. We are aware of the low quality of the data and the resulting model, the goal of the pipeline is to obtain useful information from these “failed” assemblies.
The driving hypothesis of this project is that hybridization and other non-canonical genomic architectures are very common in Fungi, but these phenomena are often not observed because they interfere with common genome assembly protocols. To test that hypothesis we have applied our pipeline to low quality (highly fragmented), publicly available genome assemblies and reported any detected biological factor that might explain why these assemblies have such a low quality. As such, we do not have the original strains available for cultivation, so we cannot test experimentally our findings or produce additional sequencing data.

2. Given the raw WGS reads and assembled genome, another software, QUAST (http://quast.sourceforge.net/), automatically detect assembly errors and structural variations. It would be interesting to see a comparison between the findings via Karyon and via Quast.

Response. The main limitation of QUAST is that it requires a reference genome assembly, which is something we want to avoid for our software. Fungal genomes are very dynamic and the boundaries between species are ill-defined. Consequently, without a good knowledge of the intraspecific genomic variability in your species it is very difficult to select a reference that is phylogenetically close enough. Additionally, most of the species analyzed in this study have only one assembly publicly available in NCBI. QUAST does generate a series of reference-free statistics, but those same parameters are computed by some Karyon components (N50, N90 and similar).

3.This is an optional suggestion, as I realize it may not be easy to implement. The biggest limitation of Karyon is that it does not automatically detect these usual genome organization. It may be possible by comparing the de novo assemblies produced by Karyon to the reference genomes. At least such possibilities should be discussed.

Response. Using reference genomes in fungi is a tricky subject, since the genetic diversity of different strains of the same species is often very high. Also, many of these genomic architectures are often strain-dependent and will probably be absent in the reference. For example, aneuploidies are often associated with adaptation to stress such as unusual carbon sources or antifungals. Hybrids are often classified as one of the parental strains, since the phylogenetic signal is mixed.

Reviewer #2: The technical note 'Karyon: a computational framework for the diagnosis of hybrids, aneuploids, and other non-standard architectures in genome assemblies' by Naranjo-Ortiz and colleagues reports on the development and application of the Karyon framework. Karyon is a python-based toolkit that utilizes several software tools developed by the authors' and/or others with the overall aim to assess sequencing data and genome assemblies for potential assembly artefacts caused by a plethora of different features intrinsic to the analyzed species/strain. Karyon is publicly available from github and as a docker image.

Genome assemblies are nowadays important tools to develop novel biological hypotheses. However, genome assemblies are often not ideal, i.e., they are highly fragmented and/or incomplete, which can significantly hamper their full exploitation. The genome assembly quality is impacted by different biological factors that can be, at least partially, discovered directly based on the raw sequencing data and from the genome assembly (e.g., allele frequency, k-mer profiles, coverage depth, etc.). There are already plenty of established computational tools available to perform these type of analyses (to name a few: KAT, genomscope, nQuire). Karyon will ease these analyses by providing a single computation framework that combines different and complex software tool and generates diagnostic figures to support biological interpretation. Karyon thus represents a valuable contribution to the scientific community.

Response. We thank the Reviewer for his/her overall positive remarks on our tool.

The Karyon toolkit is built around established software tools and the overall methodology is sound and suitable to assess genome qualities. The interpretation of the results of Karyon is on the user, which still necessitates expert knowledge to correctly interpret signals. While examples are provided in the manual, the level of experience required will likely hamper the full exploitation of the pipeline by not expert users. Furthermore, it can be anticipated that expert users already employ the separate software to study genome complexities, and thus might not be in full need for Karyon. Obviously, this is inherent to the problem at hand and cannot be easily addressed by the authors. However, I would like to encourage the authors to further improve the manual and the examples to guide the data interpretation with the aim to make this software as accessible to as many researchers as possible.

R. In order to improve user experience, Karyon now generates a report file that summaries the most important statistics in an easy to interpret way. The manual has been updated with instructions on how to interpret this file and some solutions for certain scenarios. Karyon has been used for our own group to deal with problematic assemblies. The pipeline has been presented in several international congresses, where various researchers had manifested their interest in its applications and provided feedback. Note that the authors are mainly involved in fungal genomic projects, and despite their high expertise the pipeline is now used broadly in the group as it very much facilitates our work. So the interest for expert

users should not be neglected. Producing the data required for de novo genome assemblies of fungi is relatively cheap, meaning that many fungal genome projects are produced by small researcher groups or are the main project of researchers in training. We consider this pipeline will be useful for a broad community.

I nevertheless also have some comments related to the data presented in the manuscript that the authors need to address.

First, the introduction finishes by asserting that different biological factors are expected to impact published genome assemblies. Furthermore, the manuscript mentions that quality of fungal genomes is often sub-optimal. However, no evidence for these statements is provided. To strengthen this point and to further highlight the urgency of methods to discover and ultimately address these problems, the authors need to provide a more systematic analyses based on publicly available genome assemblies for the occurrence of compromised genome assemblies. For example, a random subset of genome sequences for different eukaryotic phyla and / or classes, and more systematic throughout the fungi, would i) significantly substantiate the manuscript's message and ii) confirm the applicability of the authors' framework to most eukaryotes and not only to specific fungal groups (Mucorales).

Response. While we agree that including additional sampling would reinforce the message of the paper, we consider this aspect is secondary in the paper. The goal of the paper is to present a new tool, not to analyze genomes and to estimate what fraction of them is suboptimal. We showcase this with an example that is close to our own interests and clearly makes the point. Mucorales have genome sizes and gene content comparable to the average filamentous Ascomycota, although they tend to have lower GC%. The work proposed by the Reviewer actually constitutes a full research program that is out of the scope of this manuscript and we consider it not to be essential. We expect that the publication of our tool will allow replicating this type of studies in other groups.
Second, the table mentions the diagnosis derived from Karyon but simply mentions 'unknown' for most entries. Based on the manuscript is seem that these are supposedly haploid with very little heterozygosity (L279) but table 1 nevertheless reports for most species/strains strikingly different genome size estimates between the original and the Karyon-derived genome assemblies (Karyon is consistently smaller). The authors need to explain in much more depth the nature of these differences for the reported genomes. For instance, it could be that publicly deposited assemblies have been generated by a combination of different sequencing libraries and technologies that are not fully exploited by Karyon.

Response. We are now reporting cases with extreme GC%, which is a relatively common phenomenon in Mucoromycota. We are only reporting those strains with GC% below 35%, but this threshold is arbitrary and many are quite close to it. Several of the species have only one SRA library with relatively low depth of sequencing. Also, the vast majority of these problematic assemblies have been published by the same institution, which suggests that the artifacts might have a shared methodological origin. The scope of this paper is not to point fingers and accuse others of malpractice, and as such these observations have been omitted from the manuscript.

Third, one additional measure often applied to assess genome quality is genome completeness as for instance assayed by BUSCO. Karyon should include as strategy such as BUSCO to i) assess the occurrence of marker genes in the genome assemblies and ii) the duplication level of these genes as this might reveal un-collapsed alleles etc. Especially the latter is important to interpret genome size differences between original and Karyon-derived genome assemblies.

Response. We thank the Reviewer for this good suggestion, we have now implemented BUSCO in the pipeline.

Further detailed comments and suggestions to improve the manuscript:

L21: could the authors please specify what 'groups' they refer to?

Response. We have rephrased this sentence
L22: there seems to be an extra space

Response. Corrected.

L59: could the authors please specify what they mean with a 'poor assembly'. What is poor in terms of

genome assembly? Contiguity or completeness, or unresolved haplotypes, or …, or a combination of thereof?

Response: done

L63-: the authors only once refer explicitly to Fig 1 in this section. the manuscript would be clearer if they would refer to specific panels as they describe factors impacting genome assembly quality

Response: done

L66: could the authors please further substantiate their notion that most genome assemblies publicly available are formed by short-read sequencing data. This information should be readily available at NCBI and/or GOLD

Response: We now provide some numbers to substantiate that statement.

L119: the manuscript mentions pan-genomics, but the relevance of aneuploidy in these studies is not explained. The manuscript should provide a brief explanation for the importance of aneuploidy (or any form of ploidy shift) for pan-genomics

Response: done

L147: 'From' -> 'from'
Response. Corrected.

L148: 'Symbiotic' -> 'symbiotic'
Response. Corrected.

L232: the reference to nQuire should read Weiß et al. 2018.
Response. Corrected.

L302: the reference to blobtools is missing
Response. Corrected.

L349: To initiate the pipeline, was a single sequencing library or a combination of multiple libraries used?
Response. All available libraries were used. This is now specified in the text.

Table 1: The table formatting, at least in the combined pdf, seems to be broken.

Response: all these errors have been corrected.

Reviewer #3: Assembling a genome using short reads quite often cause a mixed bag of scaffolds representing uncollapsed haplotypes, collapsed haplotypes (i.e. the desired haploid genome representation) and collapsed duplicates. While there are individual software for collapsing uncollapsed haplotypes (e.g. HaploMerger2, or Redundans), there is no established workflow or standards for quality control of finished assemblies.

Naranjo-Ortiz et al. describes a pipeline attempting to make one. The Karyon pipeline is a workflow for assembling haploid reference genomes, while evaluating the ploidy levels on all scaffolds using GATK for variant calling and nQuire for a statistical method for estimating of ploidy from allelic coverage supports. I appreciated the pipeline promotes some of good habits - such as comparing k-mer spectra with the genome assembly (by KAT) or treatment of contamination (using Blobtools). Nearly all components of the pipeline are established tools, but authors also propose karyon plots - diagnostic plots for quality control of assemblies. The most interesting and novel one I have seen is a plot of SNP density vs coverage. Such plot might be helpful in identifying various changes to ploidy levels specific to subset of chromosome, as authors demonstrated on the example of several fungal genomes (Mucorales).

Response: We thank the reviewer for his/her appreciation of our work.

I attempted to run the pipeline and run in several technical issues. Authors, helped me overcoming the major ones (documented here: https://github.com/Gabaldonlab/karyon/issues/1) and I managed to generate a karyon plot for the genome of a male hexapod with X0 sex determination system. I did that, because we know well the karyotype and I suspected, the X chromosome will nicely pop-up in the karyon plot. To my surprise, although I know the scaffold coverages are very much bi-modal, I got only a single peak of coverages in the karyon plot and oddly somewhere in between the expected haploid and diploid coverages. I think it is possible I have messed up something, but I would like authors to demonstrate the tool on a known genome with known karyotype. I would propose to use a male of a species with XY or X0 sex determination system. Although it's not aneuploidy sensu stricto, it is most likely the most common within-genome ploidy variation among metazoans.

Response.
The karyon plot simply shows the distribution of coverages, and thus the problem must be at the underlying mapping data. We will be happy to discuss the specific dataset.

I would also propose authors to improve modularity of the pipeline. On my request authors added a lightweighted installation for users interested in the diagnostic plots after the assembly step, but the inputs are expected in a specific, but undocumented format, which makes a modular use rather hard. At least the documentation of the formats should improve, but in general I think it could be made more friendly to folks interested only in some smaller bits (I am happy to provide authors with the data I used).

Response: We have made improvements in the documentation, especially with regards to the format of the files used for calling allplots.py. It now includes a detailed description of each file and links that detail their structure.

Although I quite enjoyed reading the manuscript and the manual afterwards, I do think there is a lot of space for improvement. One major point is there is no formal description of the only truly innovative bit of this pipeline - the karyon plots. There is a nice philosophical overview, but the karyon plots are not explained in particular, which makes reading of the showcase study much harder. Perhaps a scheme showing the plot and annotating what is expected where would help. Furthermore, authors did a likelihood analysis of ploidy using nQuire, but they did not talk about it at all in the result section. I wonder, what's the fraction of the assembly the analysis found most likely to be aneuploid for the subset of strains that suspected to be aneuploids? Is 1000 basis sliding window big enough to carry enough signal to produce reliable assignments? In my experience, windows of this size are hard to assign ploidy to, but I usually do such analyses using coverage, not SNP
supports. However, I would like to appraise authors for the fungal showcases, I do think they are a nice genomics work, investigating and considering both biological and technical aspects appropriately. Finally, a bit smaller comment is that the introduction could a bit more to the point. Some of the sections felt a bit out of place, perhaps even unnecessary (see minor comments bellow).

Response. We have streamlined some parts of the introduction. We have also added a detailed explanation of the plots generated by karyonplots in the text, with an example in a new figure. With regards of the sliding window, it depends on the level of heterozygosity in the genome. For around 1% it works reasonably well, and even if the numbers of a particular window are unreliable, the overall trend of the graph should be informative. Additionally, the newly implemented report uses nQuire to analyze the overall genome.

More specific and minor comments are listed bellow.

Kamil S. Jaron

Minor manuscript comments:

I gave this manuscript a lot of thought, so I would like to share with you what I have figured out. However, I recognise that these writing comments listed bellow are largely matter of personal preference. I hope they will be useful for you, bit it is nothing I would like to insist on as a reviewer.

Response: We really appreciate the effort put by the reviewer

l56: An unnecessary book citation. It's not a primary source for that statement and if a reference was

made a "further reading", perhaps better to cite a recent review available online rather than a book.

Response. The citation has been removed.

l65 - 66: Is the "lower error rate" still a true statement? I don't think it is, error rates of HiFi reads are similar or even lower compared to short reads. (tough I do agree there is still plenty of use for short reads).

Response:. We have removed this sentence

l68 - 72: I don't think you really need this confusing statement " which are mainly influenced by the number of different k-mers", the problems of short read assembly are well explained bellow. However, I actually did not understand why the whole paragraph l76 - 88 was important. I would expect an introduction to cover approaches people use till now to overcome problems of ploidy and heterozygosity in assemblies.

Response:. We have removed this sentence. The paragraph has been edited, with references to other sequencing technologies moved to the discussion.

l176 - 177: "Ploidy can be easily estimated with cytogenetic techniques" - I don't think this statement is universally true. There are many groups where cytogenetics is extremely hard (like notoriously difficult nematodes) or species that don't cultivate in the lab. For those it's much easier to do NGS analysis. You actually contradict this "easily" right in the next sentence.

Response:.. Removed “easily”. The text now incides better in the fact that cytogenetic techniques are very difficult to apply to certain groups.

l191: the first autor of nQUire is not Weib, but Weiß. The same typo is in the reference list.
R. Corrected

l222 - 223: and l69-70 explains what is a k-mer twice.

Response:. The first reference to k-mers has been removed.

l266 - 267: This statement or the list does not contain references to publications sequencing the original genomes. I am not sure, but when possible, it is good to credit original authors for the sequencing efforts.
R. References added to table 1

l302: REF instead of a reference

Response:.. Corrected

l303: What is "important fraction"?

Response:.. Changed to “widespread bacterial contamination”

l304: How can you make such a conclusion? Did you try to remove the contamination and redo the assembly step? Did the assembly improve? Not sure if it's so important for the manuscript, but I would tone down this statement ("could be caused by" sounds more appropriate).

Response:.. Corrected

l310: "B9738 is haploid" are you talking about the genome or the assembly? How could you tell the difference between homozygous diploid and haploid genome? If there is a biological reason why homozygous diploid is unlikely, it should be mentioned.

Response:.. More often than not these fungi are haploid, but it is true that we cannot rule out an autodiploid (since they are normally haploid, when they suffer whole genome duplication they become autodiploid) from the data we have. We have added a clarification estating that a highly homozygous diploid is also possible.

l342: How fig 7 shows 3% heterozygosity? How was the heterozygosity measured? Also, karyon plot actually shows that majority of the genome is extremely homozygous and all heterozygosity is in windows with spuriously high coverage. What do you think is the haploid / diploid sequencing coverage in this case?

Response:.. This refers to the maximum of the second peak of heterozygosity according to the graph in the upper x axis. This assumes two populations, one haploid (first peak, around 1SNP/Kbp or 0.1% heterozygosity) and a diploid (highly variable heterozygosity, but the peak is roughly 30SNPs/Kbp, which is 3% heterozygosity).

l343 - 345: I don't think these statements are appropriately justified. The analysis presented did not convincingly show the genome is triploid or heterozygous diploid.

Response:.. I agree, and I don't how that interpretation came to be. The genome is a mix haploid and diploid. The text now reflects that conclusion.

l350: I think citing SRA is rather unnecessary.

Response:.. Removed reference

l358: what "model"? How could one reproduce the analysis / where could be the model found?

Response:.. Augustus is an ab initio gene predictor that uses a Hidden Markov Model to detect genes. By default the program incorporates a number of readily available models, one of which is Rhizopus oryzae, which we used. We have modified the sentence to improve clarity.

l378 - 379: Does Karyon analyse ploidy variation "during" the assembly process? Although the process is integrated in a streamlined pipeline, there are loads of approaches to detect karyotype changes in assemblies, from nQuire which is used by Karyon, through all the sex-chromosome analyses, such as https://journals.plos.org/plosbiology/article?id=10.1371/journal.pbio.1002078.

Response:.. No. All analyses are done after the assembly. We agree the sentence is misleading and we have modified it.

Method/manual comments:

Scaffold length plots have no label of the x axis. As the plots are called distributions, I would expect frequency or probability on the y axis and the scaffold length on the x. Furthermore, plotting of my own data resulted in a linnear plot with a very overscaled y-axis.

Response:.. This plot is a barplot, where we represent the scaffolds sorted by length versus the length of such scaffold. If your data contained very few scaffolds (you mentioned before you basically had chromosomes) the plot should look weird. The misleading part is "distribution" here, and we have improved the description of the plot in the manual and the manuscript.

"Scaffold versus coverage" plot also does not have axis labels either. I would also call it scaffold length vs coverage instead. I also found the position of the illustrating picture in the manual confusing a bit (probably should be before the header of the next plot).

Response:.. Same as the previous question.

Variation vs. coverage is the main plot. It does look as a useful visualisation idea. Do I understand right that it's just numbers of SNPs vs coverage? I am confused as I thought the SNP calling is done on the reference individual and in the description you talk about homozygous variants too, what are those? Missmapped reads? Misassembled references?

Response:.. Technically there is no filter for heterozygous vs homozygous, so it might be possible that some of them are homozygous. While this should be negligible in normal circumstances, it might happen if the fasta used for reference and the sequencing libraries are not from the same sample. In any case, we have modified the text to avoid confusion.

I also wonder about "3. Diffuse cloud across both X and Y axes.", I would naturally imagine that

collapsed paralogs would have a similar pattern to the plot that was shown as an example - a smear towards both higher coverage and SNP density. I guess this is a more general comment, would you expect any different signature of collapsed paralogs and higher ploidy levels? Should not paralogy be more explicitly considered as a factor?

Response:.. It would be weird since paralog regions would behave like different ploidy levels. In any case we have added a reference in the manual to this situation after the comments on contamination, suggesting to modify the identity threshold during the reduction step.

Close
