# Supplementary material for: Karyon: a computational framework for the diagnosis of hybrids, aneuploids, and other nonstandard architectures in genome assemblies
Source: Gigascience. 2022 Oct 7;11:giac088. doi: 10.1093/gigascience/giac088 (PMC9540331; doi:10.1093/gigascience/giac088)
Supplement: giac088_Response_to_Reviewer_Comments_Revision_1 [file giac088_response_to_reviewer_comments_revision_1.pdf]

Author's Response To Reviewer Comments

Close

>Reviewer reports:
>Reviewer #1: The revised manuscript by Naranjo-Ortiz et al. has been significantly improved over the original version. I was hoping they could prove the validity of their approach, Karyon, for >discovering known unusual genomic organizations by manually inspection of the plots. I think the work is still acceptable if the authors can emphasize on the fact that Karyon only generate testable >hypothesis that could potentially be validated via employing additional experiments, such as long-read sequencing. The value of Karyo lies in the automation of several labor-intensive plot >generation.

We thank the reviewer for his/her comments

>There are still numerous spelling/wording errors that should be fixed. Here are a few:
>1. p29: non-chanonical -> non-canonical
>2. p76: two or mroe -> two or more
>3. p203: for k-mers of enough size -> for k-mers of large enough size
>4. p205: the average depth of coverage -> the average depth of genome (or sequence) coverage
>5. p267-8: genomic accidents -> genomic events

we implemented all these changes, and revise the whole ms again

>Reviewer #2: The authors have addressed most of my raised comments and suggestions. I can agree with authors' reasoning on why focusing on a single fungal group to demonstrate the usability of >the here to be presented tool is sufficient and that inclusion of additional examples would be out of scope. I don't have any further comments arising from the revisions.

We thank reviewer 2 for his/her comments

Reviewer #3: In the revised manuscripts Naranjo-Ortiz et al. present Karyon, the computational pipeline to assemble and evaluate difficult genomes. The manuscript is a lot easier to read and I really appreciate the work authors made on the text. All the major concerns I raised before were fixed in the text and although I left underneath a few minor writing comments.

>I would also like to highlight that I think this pipeline will be highly relevant to many people and therefore I do think this work should be (at some point) published!
>However, one crucial aspect authors still need to address. That is the installation process of the tool. Although I spend quite some time installing, I still did not manage to run the tool even with help of >the authors. I even tried to pull the docker image, but I could not even see it online. I really think that is unacceptable in a manuscript presenting a pipeline.
>I would like authors to test the installation on a different computational node, or virtual environment (e.g. virtal box) stripped of longing credentials and their own local settings. They should make sure >their installation instructions run smoothly on generic linux settings. Preferably without using distribution-specific package managers (like apt-get). Also the repository used to build the docker image >should be available too. Furthermore, having tested and smooth installation will help authors to maintain support for the tool.

We thank the reviewer for the intensive testing of our tool, we have now completely change the installation so that dependencies are not a problem, we have answered the users in the github page and improved the manual.

>Writing:

>l.55 extra space before ., on many places within the manuscript.
>l.58 "i. e." is usually spelled without the space "i.e."
>l.69 "Heterozygosity implies existence of allelic differences within an individual." Heterozygosity is a measure of allelic differences within an individual. I find word "implies" here confusing. But also
>unnecessary sentence I think.
>l.76 typo "mroe" -> "more"
>l.76 "sexual recombination" was a quite confusing word in the sentence, is it fungi-specific term? What about "hybridisation" instead? (also to make it consistent with Figure 1)
>l.86 typo "associated to" -> "associated with"
>l.89 - 91, if we talk about per-site number of states (i.e. not phased genomes), AAB and ABB are exactly the same type of heterozygosity, as A and B labelling is usually arbitrary. However, triploid sites also can be of ABC type, which indeed makes it two possible heterozygous states.
>l.96-97, "paradigm is clearly false for many fungal" also for some nematode lineages (e.g. https://www.nature.com/articles/6800794). This is not a suggestion for the manuscript, just thought it would be interesting to point out that it happens among animals too.
>l190: "As dipSPAdes was specially designed to deal with highly polymorphic genomes (Safonova, Bankevich, and Pevzner 2015)□ ." is not a full sentence.
>l.203 - 204: "In a haploid genome, for k-mers of enough size, most k-mers will appear either one or zero times, with unique k-mers having an average coverage roughly equal to the average depth of coverage." This is a very confusing statement, partially for the chosen wording, but also because "most k-mers" with refers to all possible k-mers of certainly length (so 4^k / 2), while "unique k-mers" afterwards refers to k-mers associated with a unique genomic position. Finally, the roughly equal to coverage is a bit of oversimplification (k-mer coverage is the genomic coverage times (read length - k + 1) divided by the real length).

We have implemented all these corrections

Close
