# Supplementary material for: Karyon: a computational framework for the diagnosis of hybrids, aneuploids, and other nonstandard architectures in genome assemblies
Source: Gigascience. 2022 Oct 7;11:giac088. doi: 10.1093/gigascience/giac088 (PMC9540331; doi:10.1093/gigascience/giac088)
Supplement: giac088_Response_to_Reviewer_Comments_Revision_2 [file giac088_response_to_reviewer_comments_revision_2.pdf]

Author's Response To Reviewer Comments

Close

Dear Toni,

Your revised manuscript was again with reviewer #3, and unfortunately the reviewer still had problems installing the software. Both using the docker image and the stand-alone code failed, although the reviewer said he had spent an entire working day trying to make it work. Please see his latest report below this email.

I'm not quite sure what the problem is, and I now double checked with reviewer 1, who used your latest version of the code and the manual to successfully install the tool on an Ubuntu system.

As all reviewers like the method, and reviewer 1 confirmed that it runs on his system, we're likely going to accept the Technical Note, but before we proceed I hope you can think of ways to further improve the installation process and manual.

Response: We are sorry that some installation problems remained for reviewer 3. We have taken this issue very seriously and have performed additional efforts to ease the installation process. We have updated the installation script by creating separated conda environments for the different conflicting dependencies. We hope this will make the installation much easier. We have needed the help of one more person from my group, who is now included as a co-author. Details about the new implementations are described in the response to reviewer 3. We have also solved the minor comments from reviewer 1.

After testing the installation, reviewer 1 also mentioned two recommendations that I hope you can address:

1. The package does not include standard test datasets, and I agree with the reviewer it would be good to provide those.

Response: The manual now refers to one of the libraries used for the analyses in the text, that can be downloaded through the SRA toolkit and be used as input for test analyses. We agree that we could have been more clear about how to obtain the test data and we have also added the commands we used to the github README.

2. "One minor comment: the main script "bin/karyon.py" is executable, its first line needs to be sth. like this

#!/bin/env python "

Response: This has been added

Reviewer reports:
Reviewer #3:
In the revised work, the important visible changes are in the actual GitHub repository of the presented tool. Authors removed some of the problematic sections for installation of the tool, but not all. I have (again!) tried to 1. run the docker image, which is at least visible, but not functional. 2. I tried to install the tool locally and still faced many issues. I overcame some, but even after one whole working day and a few shorter attempts, I did not manage to run it through on their test dataset.

Therefore my first and only major comment remains unresolved. The presented tool is still not easily deployable. Not as a docker image, not via local installation.

The idea is great, I thought it's a rather creative way to attack intra-individual ploidy variation. But I simply can't recommend a non-functional pipeline for publication. I am really sorry about this!

Response: We are sorry for the remaining problems. As us and other reviewers successfully used the tool on different systems we think it may be related to some specific incompatibilities. We have therefore extensively tested the installation and performed additional changes to make it easier. We have updated the installation script by creating separated conda environments for the different conflicting dependencies. We hope this will make the installation much more intuitive and simple. For the Docker installation we have updated the docker_install.sh script to install all necessary dependencies within the docker container and the Github README to reflect this change. We have also updated the steps on how to set up the Docker environment to account for all the above changes. Moreover, in addition to all the above, we have also updated the pipeline to take advantage of the conda environments added. Users should carefully follow README instructions in the github repository or the Karyon manual, and follow the installation instructions precisely.

Close
