# Supplementary material for: Karyon: a computational framework for the diagnosis of hybrids, aneuploids, and other nonstandard architectures in genome assemblies
Source: Gigascience. 2022 Oct 7;11:giac088. doi: 10.1093/gigascience/giac088 (PMC9540331; doi:10.1093/gigascience/giac088)
Supplement: giac088_Response_to_Reviewer_Comments_Revision_3 [file giac088_response_to_reviewer_comments_revision_3.pdf]

Author's Response To Reviewer Comments

Close

We added all the editorial changes reuested.

Close
