# Supplementary material for: Karyon: a computational framework for the diagnosis of hybrids, aneuploids, and other nonstandard architectures in genome assemblies
Source: Gigascience. 2022 Oct 7;11:giac088. doi: 10.1093/gigascience/giac088 (PMC9540331; doi:10.1093/gigascience/giac088)
Supplement: giac088_Reviewer_1_Report_Original_Submission — Zhong Wang -- 6/29/2021 Reviewed [file giac088_reviewer_1_report_original_submission.pdf]

Reviewer Report

Title: Karyon: a computational framework for the diagnosis of hybrids, aneuploids, and other non-standard architectures in genome assemblies.

Version: Original Submission **Date:** 6/29/2021

Reviewer name: Zhong Wang

Reviewer Comments to Author:

In this work, Naranjo-Ortiz et al. presented a software pipeline that is capable of de novo genome assembly, variant calling, and generating diagnostic plots. Applying this software to 35 publically available, highly fragmented fugal genome assemblies revealed prevalent inconsistencies between the sequencing data and the assembly.
I really appreciate the authors' effort to make their software, Karyon, easy to use by providing multiple ways to install and a detailed software manual. I especially like the detailed explanation of how to use the diagnostic plots to infer the "nonstandard genome architectures". The manuscript is clearly written and very easy to follow. I have the following general comments:
1. It wasn't clear to me the relationships between the raw sequencing data and the assembly -- were they belong to the same isolate? If so, then the inconsistencies may reflect assembly errors in the fungal genome assembly. Have the authors rule our this possibility? The fact that these genomes are highly fragmented suggests they likely contain many errors. If they were from different isolates, then I agree with the authors that the diagnostic plots could be examined carefully to detect structural variations. For that, have the authors used any alternative method to validate at least some of their findings? To establish the validity of their approach, it would be more convincing to obtain the same findings using independent approaches, including experimental ones.
2. Given the raw WGS reads and assembled genome, another software, QUAST (http://quast.sourceforge.net/), automatically detect assembly errors and structural variations. It would be interesting to see a comparison between the findings via Karyon and via Quast.
3.This is an optional suggestion, as I realize it may not be easy to implement. The biggest limitation of Karyon is that it does not automatically detect these usual genome organization. It may be possible by comparing the de novo assemblies produced by Karyon to the reference genomes. At least such possibilities should be discussed.

Level of Interest

Please indicate how interesting you found the manuscript: Choose an item.

Quality of Written English

Please indicate the quality of language in the manuscript: Choose an item.

Declaration of Competing Interests

Please complete a declaration of competing interests, considering the following questions:

- Have you in the past five years received reimbursements, fees, funding, or salary from an organisation that may in any way gain or lose financially from the publication of this manuscript, either now or in the future?
- Do you hold any stocks or shares in an organisation that may in any way gain or lose financially from the publication of this manuscript, either now or in the future?
- Do you hold or are you currently applying for any patents relating to the content of the manuscript?
- Have you received reimbursements, fees, funding, or salary from an organization that holds or has applied for patents relating to the content of the manuscript?
- Do you have any other financial competing interests?
- Do you have any non-financial competing interests in relation to this paper?

If you can answer no to all of the above, write 'I declare that I have no competing interests' below. If your reply is yes to any, please give details below.

'I declare that I have no competing interests

I agree to the open peer review policy of the journal. I understand that my name will be included on my report to the authors and, if the manuscript is accepted for publication, my named report including any attachments I upload will be posted on the website along with the authors' responses. I agree for my report to be made available under an Open Access Creative Commons CC-BY license (http://creativecommons.org/licenses/by/4.0/). I understand that any comments which I do not wish to be included in my named report can be included as confidential comments to the editors, which will not be published.

Choose an item.

To further support our reviewers, we have joined with Publons, where you can gain additional credit to further highlight your hard work (see: https://publons.com/journal/530/gigascience). On publication of this paper, your review will be automatically added to Publons, you can then choose whether or not to claim your Publons credit. I understand this statement.

Yes Choose an item.
