# Supplementary material for: Karyon: a computational framework for the diagnosis of hybrids, aneuploids, and other nonstandard architectures in genome assemblies
Source: Gigascience. 2022 Oct 7;11:giac088. doi: 10.1093/gigascience/giac088 (PMC9540331; doi:10.1093/gigascience/giac088)
Supplement: giac088_Reviewer_2_Report_Original_Submission — Michael F. Seidl -- 7/4/2021 Reviewed [file giac088_reviewer_2_report_original_submission.pdf]

Reviewer Report

Title: Karyon: a computational framework for the diagnosis of hybrids, aneuploids, and other non-standard architectures in genome assemblies.

Version: Original Submission **Date:** 7/4/2021

Reviewer name: Michael F. Seidl

Reviewer Comments to Author:

The technical note 'Karyon: a computational framework for the diagnosis of hybrids, aneuploids, and other non-standard architectures in genome assemblies' by Naranjo-Ortiz and colleagues reports on the development and application of the Karyon framework. Karyon is a python-based toolkit that utilizes several software tools developed by the authors' and/or others with the overall aim to assess sequencing data and genome assemblies for potential assembly artefacts caused by a plethora of different features intrinsic to the analyzed species/strain. Karyon is publicly available from github and as a docker image.

Genome assemblies are nowadays important tools to develop novel biological hypotheses. However, genome assemblies are often not ideal, i.e., they are highly fragmented and/or incomplete, which can significantly hamper their full exploitation. The genome assembly quality is impacted by different biological factors that can be, at least partially, discovered directly based on the raw sequencing data and from the genome assembly (e.g., allele frequency, k-mer profiles, coverage depth, etc.). There are already plenty of established computational tools available to perform these type of analyses (to name a few: KAT, genomscope, nQuire). Karyon will ease these analyses by providing a single computation framework that combines different and complex software tool and generates diagnostic figures to support biological interpretation. Karyon thus represents a valuable contribution to the scientific community.

The Karyon toolkit is built around established software tools and the overall methodology is sound and suitable to assess genome qualities. The interpretation of the results of Karyon is on the user, which still necessitates expert knowledge to correctly interpret signals. While examples are provided in the manual, the level of experience required will likely hamper the full exploitation of the pipeline by not expert users. Furthermore, it can be anticipated that expert users already employ the separate software to study genome complexities, and thus might not be in full need for Karyon. Obviously, this is inherent to the problem at hand and cannot be easily addressed by the authors. However, I would like to encourage the authors to further improve the manual and the examples to guide the data interpretation with the aim to make this software as accessible to as many researchers as possible.

I nevertheless also have some comments related to the data presented in the manuscript that the authors need to address.

First, the introduction finishes by asserting that different biological factors are expected to impact published genome assemblies. Furthermore, the manuscript mentions that quality of fungal genomes is often sub-optimal. However, no evidence for these statements is provided. To strengthen this point and to further highlight the urgency of methods to discover and ultimately address these problems, the

authors need to provide a more systematic analyses based on publicly available genome assemblies for the occurrence of compromised genome assemblies. For example, a random subset of genome sequences for different eukaryotic phyla and / or classes, and more systematic throughout the fungi, would i) significantly substantiate the manuscript's message and ii) confirm the applicability of the authors' framework to most eukaryotes and not only to specific fungal groups (Mucorales).
Second, the table mentions the diagnosis derived from Karyon but simply mentions 'unknown' for most entries. Based on the manuscript is seem that these are supposedly haploid with very little heterozygosity (L279) but table 1 nevertheless reports for most species/strains strikingly different genome size estimates between the original and the Karyon-derived genome assemblies (Karyon is consistently smaller). The authors need to explain in much more depth the nature of these differences for the reported genomes. For instance, it could be that publicly deposited assemblies have been generated by a combination of different sequencing libraries and technologies that are not fully exploited by Karyon.
Third, one additional measure often applied to assess genome quality is genome completeness as for instance assayed by BUSCO. Karyon should include as strategy such as BUSCO to i) assess the occurrence of marker genes in the genome assemblies and ii) the duplication level of these genes as this might reveal un-collapsed alleles etc. Especially the latter is important to interpret genome size differences between original and Karyon-derived genome assemblies.
Further detailed comments and suggestions to improve the manuscript:
L21: could the authors please specify what 'groups' they refer to?
L22: there seems to be an extra space
L59: could the authors please specify what they mean with a 'poor assembly'. What is poor in terms of genome assembly? Contiguity or completeness, or unresolved haplotypes, or …, or a combination of thereof?
L63-: the authors only once refer explicitly to Fig 1 in this section. the manuscript would be clearer if they would refer to specific panels as they describe factors impacting genome assembly quality
L66: could the authors please further substantiate their notion that most genome assemblies publicly available are formed by short-read sequencing data. This information should be readily available at NCBI and/or GOLD
L119: the manuscript mentions pan-genomics, but the relevance of aneuploidy in these studies is not explain. The manuscript should provide a brief explanation for the importance of aneuploidy (or any form of ploidy shift) for pan-genomics
L147: 'From' -> 'from'
L148: 'Symbiotic' -> 'symbiotic'
L232: the reference to nQuire should read WeiÃŸ et al. 2018.
L302: the reference to blobtools is missing
L349: To initiate the pipeline, was a single sequencing library or a combination of multiple libraries used?
Table 1: The table formatting, at least in the combined pdf, seems to be broken.

Level of Interest

Please indicate how interesting you found the manuscript: Choose an item.

Quality of Written English

Please indicate the quality of language in the manuscript: Choose an item.

Declaration of Competing Interests

Please complete a declaration of competing interests, considering the following questions:

- Have you in the past five years received reimbursements, fees, funding, or salary from an organisation that may in any way gain or lose financially from the publication of this manuscript, either now or in the future?
- Do you hold any stocks or shares in an organisation that may in any way gain or lose financially from the publication of this manuscript, either now or in the future?
- Do you hold or are you currently applying for any patents relating to the content of the manuscript?
- Have you received reimbursements, fees, funding, or salary from an organization that holds or has applied for patents relating to the content of the manuscript?
- Do you have any other financial competing interests?
- Do you have any non-financial competing interests in relation to this paper?

If you can answer no to all of the above, write 'I declare that I have no competing interests' below. If your reply is yes to any, please give details below.

I declare that I have no competing interests

I agree to the open peer review policy of the journal. I understand that my name will be included on my report to the authors and, if the manuscript is accepted for publication, my named report including any attachments I upload will be posted on the website along with the authors' responses. I agree for my report to be made available under an Open Access Creative Commons CC-BY license (http://creativecommons.org/licenses/by/4.0/). I understand that any comments which I do not wish to be included in my named report can be included as confidential comments to the editors, which will not be published.

Choose an item.

To further support our reviewers, we have joined with Publons, where you can gain additional credit to further highlight your hard work (see: https://publons.com/journal/530/gigascience). On publication of this paper, your review will be automatically added to Publons, you can then choose whether or not to claim your Publons credit. I understand this statement.

Yes Choose an item.
