# Supplementary material for: Karyon: a computational framework for the diagnosis of hybrids, aneuploids, and other nonstandard architectures in genome assemblies
Source: Gigascience. 2022 Oct 7;11:giac088. doi: 10.1093/gigascience/giac088 (PMC9540331; doi:10.1093/gigascience/giac088)
Supplement: giac088_Reviewer_3_Report_Original_Submission — Kamil S. Jaron -- 7/10/2021 Reviewed [file giac088_reviewer_3_report_original_submission.pdf]

Reviewer Report

Title: Karyon: a computational framework for the diagnosis of hybrids, aneuploids, and other non-standard architectures in genome assemblies.

Version: Original Submission **Date:** 7/10/2021

Reviewer name: Kamil S. Jaron

Reviewer Comments to Author:

Assembling a genome using short reads quite often cause a mixed bag of scaffolds representing uncollapsed haplotypes, collapsed haplotypes (i.e. the desired haploid genome representation) and collapsed duplicates. While there are individual software for collapsing uncollapsed haplotypes (e.g. HaploMerger2, or Redundans), there is no established workflow or standards for quality control of finished assemblies.
Naranjo-Ortiz et al. describes a pipeline attempting to make one. The Karyon pipeline is a workflow for assembling haploid reference genomes, while evaluating the ploidy levels on all scaffolds using GATK for variant calling and nQuire for a statistical method for estimating of ploidy from allelic coverage supports. I appreciated the pipeline promotes some of good habits - such as comparing k-mer spectra with the genome assembly (by KAT) or treatment of contamination (using Blobtools). Nearly all components of the pipeline are established tools, but authors also propose karyon plots - diagnostic plots for quality control of assemblies. The most interesting and novel one I have seen is a plot of SNP density vs coverage. Such plot might be helpful in identifying various changes to ploidy levels specific to subset of chromosome, as authors demonstrated on the example of several fungal genomes (Mucorales).
I attempted to run the pipeline and run in several technical issues. Authors, helped me overcoming the major ones (documented here: https://github.com/Gabaldonlab/karyon/issues/1) and I managed to generate a karyon plot for the genome of a male hexapod with X0 sex determination system. I did that, because we know well the karyotype and I suspected, the X chromosome will nicely pop-up in the karyon plot. To my surprise, although I know the scaffold coverages are very much bi-modal, I got only a single peak of coverages in the karyon plot and oddly somewhere in between the expected haploid and diploid coverages. I think it is possible I have messed up something, but I would like authors to demonstrate the tool on a known genome with known karyotype. I would propose to use a male of a species with XY or X0 sex determination system. Although it's not aneuploidy sensu stricto, it is most likely the most common within-genome ploidy variation among metazoans.
I would also propose authors to improve modularity of the pipeline. On my request authors added a lightweighted installation for users interested in the diagnostic plots after the assembly step, but the inputs are expected in a specific, but undocumented format, which makes a modular use rather hard. At least the documentation of the formats should improve, but in general I think it could be made more friendly to folks interested only in some smaller bits (I am happy to provide authors with the data I used).
Although I quite enjoyed reading the manuscript and the manual afterwards, I do think there is a lot of space for improvement. One major point is there is no formal description of the only truly innovative bit

of this pipeline - the karyon plots. There is a nice philosophical overview, but the karyon plots are not explained in particular, which makes reading of the showcase study much harder. Perhaps a scheme showing the plot and annotating what is expected where would help. Furthermore, authors did a likelihood analysis of ploidy using nQuire, but they did not talk about it at all in the result section. I wonder, what's the fraction of the assembly the analysis found most likely to be aneuploid for the subset of strains that suspected to be aneuploids? Is 1000 basis sliding window big enough to carry enough signal to produce reliable assignments? In my experience, windows of this size are hard to assign ploidy to, but I usually do such analyses using coverage, not SNP supports. However, I would like to appraise authors for the fungal showcases, I do think they are a nice genomics work, investigating and considering both biological and technical aspects appropriately. Finally, a bit smaller comment is that the introduction could a bit more to the point. Some of the sections felt a bit out of place, perhaps even unnecessary (see minor comments bellow).
More specific and minor comments are listed bellow.
Kamil S. Jaron
Minor manuscript comments:
I gave this manuscript a lot of thought, so I would like to share with you what I have figured out. However, I recognise that these writing comments listed bellow are largely matter of personal preference. I hope they will be useful for you, bit it is nothing I would like to insist on as a reviewer.
l56: An unnecessary book citation. It's not a primary source for that statement and if a reference was made a "further reading", perhaps better to cite a recent review available online rather than a book.
l65 - 66: Is the "lower error rate" still a true statement? I don't think it is, error rates of HiFi reads are similar or even lower compared to short reads. (tough I do agree there is still plenty of use for short reads).
l68 - 72: I don't think you really need this confusing statement " which are mainly influenced by the number of different k-mers", the problems of short read assembly are well explained bellow. However, I actually did not understand why the whole paragraph l76 - 88 was important. I would expect an introduction to cover approaches people use till now to overcome problems of ploidy and heterozygosity in assemblies.
l176 - 177: "Ploidy can be easily estimated with cytogenetic techniques" - I don't think this statement is universally true. There are many groups where cytogenetics is extremely hard (like notoriously difficult nematodes) or species that don't cultivate in the lab. For those it's much easier to do NGS analysis. You actually contradict this "easily" right in the next sentence.
l191: the first autor of nQUire is not Weib, but WeiÃŸ. The same typo is in the reference list.
l222 - 223: and l69-70 explains what is a k-mer twice.
l266 - 267: This statement or the list does not contain references to publications sequencing the original genomes. I am not sure, but when possible, it is good to credit original authors for the sequencing efforts.
l302: REF instead of a reference
l303: What is "important fraction"?
l304: How can you make such a conclusion? Did you try to remove the contamination and redo the assembly step? Did the assembly improve? Not sure if it's so important for the manuscript, but I would tone down this statement ("could be caused by" sounds more appropriate).

l310: "B9738 is haploid" are you talking about the genome or the assembly? How could you tell the difference between homozygous diploid and haploid genome? If there is a biological reason why homozygous diploid is unlikely, it should be mentioned.
l342: How fig 7 shows 3% heterozygosity? How was the heterozygosity measured? Also, karyon plot actually shows that majority of the genome is extremely homozygous and all heterozygosity is in windows with spuriously high coverage. What do you think is the haploid / diploid sequencing coverage in this case?
l343 - 345: I don't think these statements are appropriately justified. The analysis presented did not convincingly show the genome is triploid or heterozygous diploid.
l350: I think citing SRA is rather unnecessary.
l358: what "model"? How could one reproduce the analysis / where could be the model found?
l378 - 379: Does Karyon analyse ploidy variation "during" the assembly process? Although the process is integrated in a streamlined pipeline, there are loads of approaches to detect karyotype changes in assemblies, from nQuire which is used by Karyon, through all the sex-chromosome analyses, such as https://journals.plos.org/plosbiology/article?id=10.1371/journal.pbio.1002078.
Method/manual comments:
Scaffold length plots have no label of the x axis. As the plots are called distributions, I would expect frequency or probability on the y axis and the scaffold length on the x. Furthermore, plotting of my own data resulted in a linnear plot with a very overscaled y-axis.
"Scaffold versus coverage" plot also does not have axis labels either. I would also call it scaffold length vs coverage instead. I also found the position of the illustrating picture in the manual confusing a bit (probably should be before the header of the next plot).
Variation vs. coverage is the main plot. It does look as a useful visualisation idea. Do I understand right that it's just numbers of SNPs vs coverage? I am confused as I thought the SNP calling is done on the reference individual and in the description you talk about homozygous variants too, what are those? Missmapped reads? Misassembled references?
I also wonder about "3. Diffuse cloud across both X and Y axes.", I would naturally imagine that collapsed paralogs would have a similar pattern to the plot that was shown as an example - a smear towards both higher coverage and SNP density. I guess this is a more general comment, would you expect any different signature of collapsed paralogs and higher ploidy levels? Should not paralogy be more explicitly considered as a factor?

Level of Interest

Please indicate how interesting you found the manuscript: Choose an item.

Quality of Written English

Please indicate the quality of language in the manuscript: Choose an item.

Declaration of Competing Interests

Please complete a declaration of competing interests, considering the following questions:

- Have you in the past five years received reimbursements, fees, funding, or salary from an organisation that may in any way gain or lose financially from the publication of this manuscript, either now or in the future?
- Do you hold any stocks or shares in an organisation that may in any way gain or lose financially from the publication of this manuscript, either now or in the future?
- Do you hold or are you currently applying for any patents relating to the content of the manuscript?
- Have you received reimbursements, fees, funding, or salary from an organization that holds or has applied for patents relating to the content of the manuscript?
- Do you have any other financial competing interests?
- Do you have any non-financial competing interests in relation to this paper?

If you can answer no to all of the above, write 'I declare that I have no competing interests' below. If your reply is yes to any, please give details below.

1. - 5. I declare that I have no competing interests. 6. I published a different method for analysis of ploidy in ngs data called Smudgeplot.

I agree to the open peer review policy of the journal. I understand that my name will be included on my report to the authors and, if the manuscript is accepted for publication, my named report including any attachments I upload will be posted on the website along with the authors' responses. I agree for my report to be made available under an Open Access Creative Commons CC-BY license (http://creativecommons.org/licenses/by/4.0/). I understand that any comments which I do not wish to be included in my named report can be included as confidential comments to the editors, which will not be published.

Choose an item.

To further support our reviewers, we have joined with Publons, where you can gain additional credit to further highlight your hard work (see: https://publons.com/journal/530/gigascience). On publication of this paper, your review will be automatically added to Publons, you can then choose whether or not to claim your Publons credit. I understand this statement.

Yes Choose an item.
