# Supplementary material for: Karyon: a computational framework for the diagnosis of hybrids, aneuploids, and other nonstandard architectures in genome assemblies
Source: Gigascience. 2022 Oct 7;11:giac088. doi: 10.1093/gigascience/giac088 (PMC9540331; doi:10.1093/gigascience/giac088)
Supplement: giac088_Reviewer_3_Report_Revision_1 — Kamil S. Jaron -- 1/10/202 Reviewed [file giac088_reviewer_3_report_revision_1.pdf]

Reviewer Report

Title: Karyon: a computational framework for the diagnosis of hybrids, aneuploids, and other non-standard architectures in genome assemblies.

Version: Revision 1 **Date:** 1/10/2022

Reviewer name: Kamil S. Jaron

Reviewer Comments to Author:

In the revised manuscripts Naranjo-Ortiz et al. present Karyon, the computational pipeline to assemble and evaluate difficult genomes. The manuscript is a lot easier to read and I really appreciate the work authors made on the text. All the major concerns I raised before were fixed in the text and although I left underneath a few minor writing comments.
I would also like to highlight that I think this pipeline will be highly relevant to many people and therefore I do think this work should be (at some point) published!
However, one crucial aspect authors still need to address. That is the installation process of the tool. Although I spend quite some time installing, I still did not manage to run the tool even with help of the authors. I even tried to pull the docker image, but I could not even see it online. I really think that is unacceptable in a manuscript presenting a pipeline.
I would like authors to test the installation on a different computational node, or virtual environment (e.g. virtal box) stripped of longing credentials and their own local settings. They should make sure their installation instructions run smoothly on generic linux settings. Preferably without using distribution-specific package managers (like apt-get). Also the repository used to build the docker image should be available too. Furthermore, having tested and smooth installation will help authors to maintain support for the tool.
Writing:
l.55 extra space before ., on many places within the manuscript.
l.58 "i. e." is usually spelled without the space "i.e."
l.69 "Heterozygosity implies existence of allelic differences within an individual." Heterozygosity is a measure of allelic differences within an individual. I find word "implies" here confusing. But also unnecessary sentence I think.
l.76 typo "mroe" -> "more"
l.76 "sexual recombination" was a quite confusing word in the sentence, is it fungi-specific term? What about "hybridisation" instead? (also to make it consistent with Figure 1)
l.86 typo "associated to" -> "associated with"
l.89 - 91, if we talk about per-site number of states (i.e. not phased genomes), AAB and ABB are exactly the same type of heterozygosity, as A and B labelling is usually arbitrary. However, triploid sites also can be of ABC type, which indeed makes it two possible heterozygous states.
l.96-97, "paradigm is clearly false for many fungal" also for some nematode lineages (e.g. https://www.nature.com/articles/6800794). This is not a suggestion for the manuscript, just thought it would be interesting to point out that it happens among animals too.

l.190: "As dipSPAdes was specially designed to deal with highly polymorphic genomes (Safonova, Bankevich, and Pevzner 2015)â .” is not a full sentence.
l.203 - 204: "In a haploid genome, for k-mers of enough size, most k-mers will appear either one or zero times, with unique k-mers having an average coverage roughly equal to the average depth of coverage." This is a very confusing statement, partially for the chosen wording, but also because "most k-mers" with refers to all possible k-mers of certainly length (so 4^k / 2), while "unique k-mers" afterwards refers to k-mers associated with a unique genomic position. Finally, the roughly equal to coverage is a bit of oversimplification (k-mer coverage is the genomic coverage times (read length - k + 1) divided by the real length).

Level of Interest

Please indicate how interesting you found the manuscript: Choose an item.

Quality of Written English

Please indicate the quality of language in the manuscript: Choose an item.

Declaration of Competing Interests

Please complete a declaration of competing interests, considering the following questions:

- Have you in the past five years received reimbursements, fees, funding, or salary from an organisation that may in any way gain or lose financially from the publication of this manuscript, either now or in the future?
- Do you hold any stocks or shares in an organisation that may in any way gain or lose financially from the publication of this manuscript, either now or in the future?
- Do you hold or are you currently applying for any patents relating to the content of the manuscript?
- Have you received reimbursements, fees, funding, or salary from an organization that holds or has applied for patents relating to the content of the manuscript?
- Do you have any other financial competing interests?
- Do you have any non-financial competing interests in relation to this paper?

If you can answer no to all of the above, write 'I declare that I have no competing interests' below. If your reply is yes to any, please give details below.

I declare that I have no competing interests

I agree to the open peer review policy of the journal. I understand that my name will be included on my report to the authors and, if the manuscript is accepted for publication, my named report including any

attachments I upload will be posted on the website along with the authors' responses. I agree for my report to be made available under an Open Access Creative Commons CC-BY license (http://creativecommons.org/licenses/by/4.0/). I understand that any comments which I do not wish to be included in my named report can be included as confidential comments to the editors, which will not be published.

Choose an item.

To further support our reviewers, we have joined with Publons, where you can gain additional credit to further highlight your hard work (see: https://publons.com/journal/530/gigascience). On publication of this paper, your review will be automatically added to Publons, you can then choose whether or not to claim your Publons credit. I understand this statement.

Yes Choose an item.
